# Tetrameric self-assembling of water-lean solvents enables carbamate anhydride-based CO_2_ capture chemistry

**DOI:** 10.1038/s41557-024-01495-z

**Published:** 2024-04-08

**Authors:** Julien Leclaire, David J. Heldebrant, Katarzyna Grubel, Jean Septavaux, Marc Hennebelle, Eric Walter, Ying Chen, Jose Leobardo Bañuelos, Difan Zhang, Manh-Thuong Nguyen, Debmalya Ray, Sarah I. Allec, Deepika Malhotra, Wontae Joo, Jaelynne King

**Affiliations:** 1 CNRS ICBMS UMR 5246, Universite Claude Bernard Lyon 1, Villeurbanne, France; 2 Pacific Northwest National Laboratory, Richland, WA USA; 3 Washington State University Pullman, Pullman, WA USA; 4 University of Texas El Paso, El Paso, TX USA; 5 Present Address: Secoya Technologies, Ottignies-Louvain-la-Neuve, Belgium; 6 Present Address: Oak Ridge National Laboratory, Oak Ridge, TN USA

**Keywords:** Self-assembly, Sustainability

## Abstract

Carbon capture, utilization and storage is a key yet cost-intensive technology for the fight against climate change. Single-component water-lean solvents have emerged as promising materials for post-combustion CO_2_ capture, but little is known regarding their mechanism of action. Here we present a combined experimental and modelling study of single-component water-lean solvents, and we find that CO_2_ capture is accompanied by the self-assembly of reverse-micelle-like tetrameric clusters in solution. This spontaneous aggregation leads to stepwise cooperative capture phenomena with highly contrasting mechanistic and thermodynamic features. The emergence of well-defined supramolecular architectures displaying a hydrogen-bonded internal core, reminiscent of enzymatic active sites, enables the formation of CO_2_-containing molecular species such as carbamic acid, carbamic anhydride and alkoxy carbamic anhydrides. This system extends the scope of adducts and mechanisms observed during carbon capture. It opens the way to materials with a higher CO_2_ storage capacity and provides a means for carbamates to potentially act as initiators for future oligomerization or polymerization of CO_2_.

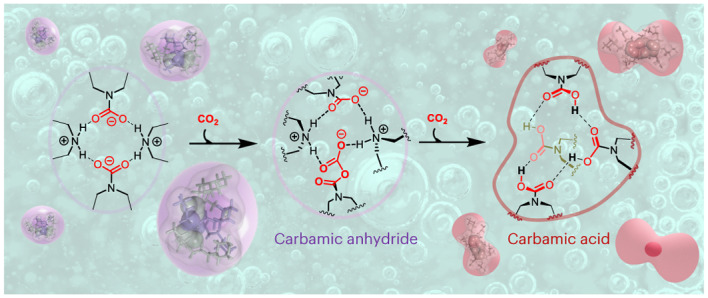

## Abstract (web summary)

Carbon capture, utilization and storage is key for climate change mitigation and developing more environmentally friendly technologies. Now it has been shown that CO_2_ capture in single-component water-lean solvents is accompanied by the self-assembly of reverse-micelle-like tetrameric clusters in solution that enable the formation of various CO_2_-containing compounds.

## Main

**Fig. 1:**
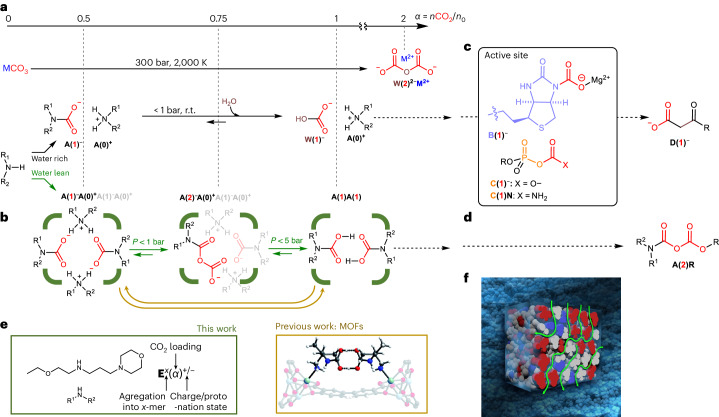
Conventional versus unconventional CO_2_-binding adducts with increasing CO_2_/amine-A stoichiometry. **a**,**b**, Conventional adducts from aqueous CO_2_ capture (ammonium carbamate **A(0)**^**+**^**A(1)**, ammonium carbonate **A(0)**^**+**^**W(1)**^**–**^ and metal percarbonate **M**^**2+**^**W(2)**^**2–**^ (**a**)) versus adducts observed in a water-lean medium such as a MOF and in a water-lean solvent (green, this work) including anhydride **A(2)**^**–**^ and carbamic acid **A(1)** (**b**). r.t., room temperature; *n*CO_2_, molar amount of CO_2_; *n*_0_ molar amount of absorbant. **c**,**d**, Biological (urea-based **B(1)**^**–**^ and phosphate-based **C(1)**^**–**^ and **C(1)N**) capture intermediates (**c**) and their final transformation products **D**(**1**)^–^ versus synthetic alkoxy carbamic anhydride **A(2)R** (**d**). **e**,**f**, Structure of EEMPA, associated notation, X-ray structure of **A(2)** dimer within MOF^9^ (**e**; reproduced from ref. ^4^ with permission of the Royal Society of Chemistry) and aggregation domains within CO_2_-loaded water-lean solvents^34^ (**f**). MOF, metal organic framework.

In its latest report, the International Energy Agency confirmed that CO_2_ capture has a critical role in greenhouse gas mitigation and the clean energy transition^1^. Solvent-based technologies are the most mature option for point source CO_2_ capture, with many commercial offerings available^2^. Yet their substantial cost hampers deployment, and the thermal regeneration of solvent would consume energy at the gigatonne scale. This high energy penalty is inherent to the CO_2_ absorption thermodynamics of aqueous amines **A**. Despite decades of research and calls for change^3,4^, scrubbing is unlikely to advance with current half-loaded ammonium carbamate **A(0)**^**+**^**A**(**1)**^**1–**^ and ammonium bicarbonate **A(0)**^**+**^**W(1)**^**–**^ adduct pairs (Fig. 1a,e for notation).

Confining amines within nanoscopic sites in a solid material (either pre-synthesized^5,6^ or assembled during capture^7^) has been the only strategy to achieve cooperative and full loading^8–10^ (into carbamic acid **A(1)**; Fig. 1b). Although carbamic acid was postulated to exist in moderately polar aprotic liquid media^11–14^, unambiguous evidence of its formation in solution is scarce^10,15,16^. The recent observation of pyrocarbonates **M**^**2+**^**W(2)**^**2–**^ (M = Pb, Sr) proved that the theoretical limit of one CO_2_ per binding site can be overcome at extreme temperatures and pressures^17^.

Biological systems cooperatively manage oxygen uptake using haemoglobin^18^, though no equivalent molecule exists to absorb CO_2_. Instead, CO_2_ is transported as a water-bound species (bicarbonate) or a dissolved gas and converted into original adducts (Fig. 1c) such as *N*-carboxybiotin **B(1)**^**–**^ (ref. ^19^) and carboxyphosphate **C(1)**^**–**^ (ref. ^20^), or carbamoylphosphate **C(1)N** (ref. ^21^). These activated species, key intermediates for the biosynthesis of the building blocks of life **D(1)**^**–**^, are stabilized by a non-covalent hydrogen bonding network within the shielded active sites of enzymes. Through this confinement strategy, nature has extended the portfolio of capture reactions and products far beyond humanity’s current achievements.

## Results and discussion

In this work, we explore the ability of a neat water-lean solvent to self-assemble into clusters with a shielded reactive site that enables atypical CO_2_ capture adduct (Fig. 1d) formation under mild conditions (temperature, *T* ≤ 313 K; CO_2_ equilibrium pressure, *P* < 15 bar; Fig. 1b). Single-component water-lean solvents, such as *N*-(2-ethoxyethyl)-3-morpholinopropan-1-amine (EEMPA, **E**), have emerged as promising for post-combustion CO_2_ capture. EEMPA has a higher solvent energy efficiency^22^ and lower capture costs^23,24^ than aqueous amines^25^ or two-component formulations in the peer-reviewed literature^26–32^. EEMPA and its diamine analogues were initially designed to form a stable intramolecularly hydrogen-bonded carbamic acid and adopt a folded hairpin structure^33^. Until now, experimental data could not support the formation of a carbamic acid under processing conditions (partial pressure p*(CO_2_) < 0.15 bar). Here we posit an alternative hypothesis, where the properties of EEMPA are directly related to its intermolecular self-assembly rather than intramolecular folding (Fig. 1b) after chemically fixing CO_2_. This is supported by the recent observation that water-lean alkoxyguanidines^34–37^ display a heterogeneous structure of aggregated ions upon loading (Fig. 1f). This spontaneous nanostructure formation may provide confined (re)active sites, likely explaining how **E** successfully performs the integrated capture and conversion of CO_2_ into fuels and chemicals including methanol^38^ and methane^39^.

### NMR-based identification of molecular and supramolecular speciation

**Fig. 2:**
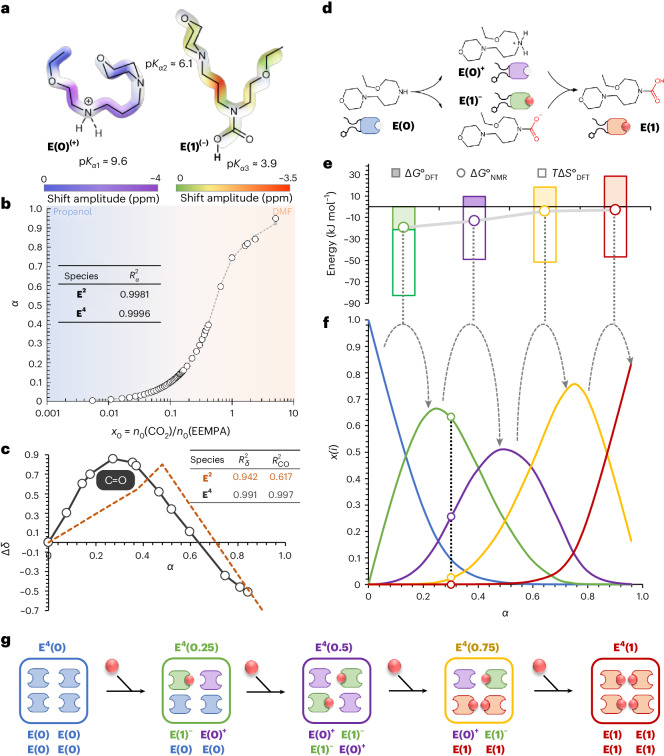
Tetrameric model based on EEMPA–CO_2_ constituents. **a**, Chemical shift perturbation mapping from q^13^C NMR analyses on **E(0)**^**(+)**^ and **E(1)**^**(−)**^ (colour code reflects chemical shift perturbation amplitude; p*K*_a1_, p*K*_a_ of the secondary amine; p*K*_a2_: p*K*_a_ of the tertiary amine; p*K*_a3_: p*K*_a_ of the carbamic acid). **b**,**c**, Fitting of the *α* and Δ*δ*(*i*) values with increasing CO_2_/EEMPA initial molar ratio *x*_0_ by the dimeric **E**^**2**^ and tetrameric **E**^**4**^ models (dot, experimental; dashed line, model; *R*^2^_*α*_, *R*^2^_*δ*_ and *R*^2^_CO_ are root mean squared deviation values for *α*, *δ*(aliphatic) and *δ*(CO), respectively; DMF, N,N-dimethylformamide). **d**, The two-stage loading process of **E(0)** by CO_2_ yielding adducts **E(0)**^**+**^, **E(1)**^**–**^ and **E(1)**. **e**, CO_2_-binding Gibbs free energies (Δ*G*°_DFT_; filled bars), entropies (*T*Δ*S*°_DFT_; empty bars) and enthalpies (sum) obtained from DFT and the thermodynamic model (Δ*G*°_NMR_; circles). **f**, The resulting speciation (*x*(*i*), the molar fraction versus *α*, the CO_2_ loading; vertical dotted line, loading selected for classical molecular dynamics modelling). **g**, Notation and schematic representation of oligomers and monomeric constituents. Source data

To elucidate the molecular features of CO_2_ capture by **E** and identify the supramolecular interactions during self-aggregation, quantitative ^1^H and ^13^C NMR spectra were recorded on a series of samples of neat **E** mixed with increasing molar ratios, *x*_0_, of CO_2_. Two sets of signals in slow exchange at the NMR timescale were observed. The signals correspond to free ammonium/amine and carbamic acid/carbamate pairs (respectively notated as **E(0)**^**(+)**^ and **E(1)**^**(–)**^; Supplementary Tables 1 and 2 for nomenclature), the former converting to the latter throughout CO_2_ absorption (Fig. 2a and Supplementary Figs. 12–17). Accurately quantifying both species produced the loading, *α*, that is, the molar fraction of CO_2_ effectively bound by **E**. Complemented at low *x*_0_ values by vapour–liquid equilibrium measurements (Supplementary Fig. 2)^22^, these data (Supplementary Fig. 5 and Supplementary Table 6) allowed us to plot the CO_2_ binding isotherm with its sigmoidal profile, typical of cooperative systems (Fig. 2b and Supplementary Figs. 10 and 11)^7,8^. Initial partial pressures of up to 70 bar CO_2_ (5 equiv. CO_2_ versus **E**) were required to reach a final *α* of almost 1, well above the 0.5 CO_2_ per EEMPA in unpressurized flue gas^14^. Under the studied experimental conditions, slow CO_2_ absorption by **E**, whose viscosity increases slightly, eventually leads to equilibrium pressure values no higher than 15 bar (Supplementary Figs. 6 and 47) and water content no higher than 700 ppm (an H_2_O:EEMPA molar ratio of <1:120). During loading, NMR peaks of both **E(0)**^**(+)**^ and **E(1)**^**(–)**^ underwent noticeable chemical shift perturbations (Supplementary Figs. 22–25) due to fast protonation and self-aggregation phenomena. Chemical shift perturbation mapping^40^ (Fig. 2a) and monitoring^41^ (Fig. 2c) confirmed that protonation occurred first on the most basic site (the secondary nitrogen atom of **E(0)**; Supplementary Figs. 1 and 4**)**, as expected, and then unexpectedly on the least basic site (the carbamic oxygen of **E(1)**; Fig. 2c, inset). Together, these observations can be translated into a two-stage molecular scenario (Fig. 2d). Below partial pressures in CO_2_ of 1 bar, neat **E(0)** yields an equimolecular mixture of charged ammonium **E(0)**^**+**^ and carbamate **E(1)**^**–**^, which convert into pure carbamic acid **E(1)** upon gentle pressurization (equilibrium partial pressure p*_f_(CO_2_) < 20 bar; Supplementary Fig. 7). A convergent set of evidence (strong shielding of the carbonyl carbon^42^ and strong deshielding of the hydroxyl proton^11^ peaks in ^13^C and ^1^H NMR, respectively; Supplementary Figs. 15 and 17) supports the presence of this elusive adduct. The data indicate that carbamic acid formation begins at unexpectedly low loading values (around 0.3; Fig. 2c, inset). Experimental data, which encompass chemical shift perturbations Δ*δ*(*i*), chemical loading values *α* and molar ratios *x*_0_, were fitted with a MATLAB in-house script (see Supplementary Fig. 3 for numbering *i* of each proton and carbon). Data processing provided the equilibrium binding constants of the two-stage covalent process and values of the chemical shifts *δ*(*i*) of the individual **E(0)**, **E(0)**^+^, **E(1)**^**–**^ and **E(1)** adducts (Supplementary Figs. 26 and 27). Interestingly, a conventional dimeric model (Fig. 2d) could not be reliably fitted to the experimental data (in particular the ^13^C chemical shift perturbation of the carbonyl group of **E(1)**^**(–)**^; Fig. 2c, inset) in the absence or presence of an additional **E(1)**–**E(1)** dimerization equilibrium. Of all scenarios involving higher aggregates, the tetrameric model (Fig. 2g) provided the best match with the full set of experimental data (Fig. 2b,c, Supplementary Figs. 10, 11 and 22–25 and Supplementary Table 7).

### Thermodynamic analysis of self-assembly and CO_2_ absorption

The full picture of the covalent adduct and non-covalent cluster populations, or chemical speciation, could be simulated after parameter adjustment through fitting the NMR data. Figure 2f,e shows the speciation and Gibbs free energies of CO_2_ absorption for each successive tetramer **E**^**4**^**(*α*)**, respectively (Δ*G*°_DFT_, DFT-computed Gibbs free energy; Δ*G*°_NMR_, Gibbs free energy computed from NMR data; *T*Δ*S*°_NMR_, entropic contribution from NMR data). This information, derived from a thermodynamic model, is fully consistent with the experimental observations. Simulations (Fig. 2e, yellow curve), in agreement with NMR data (Fig. 2c), show that carbamic acid is a key component of the cluster **E**^**4**^**(0.75)** that emerges as loading reaches 0.3. Although EEMPA displays clustering similarly to haemoglobin, its stepwise CO_2_ capture is negatively cooperative (Fig. 2e), with a drop in Gibbs free energy of ~7 kJ mol^–1^ between the first, second and third binding steps. The free energy reduction explains why pressurization is needed, despite the thermodynamic stabilization provided by tetrameric self-assembly. Energies and structures of each covalent species, **E(0)**, **E(0)**^**+**^, **E(1)**^**–**^ and **E(1)**, as well as of their dimeric and tetrameric clusters were assessed by density functional theory (DFT) calculations with an implicit model of the EEMPA–CO_2_ medium^43^. This approach provides a good estimation of energies, which can be further refined by explicitly taking into account the molecules surrounding the computed structures. This preliminary campaign was complemented with classical molecular dynamics simulations. Thermodynamic data of the non-covalent (dimerization and tetramerization) and covalent processes (CO_2_ capture) were obtained from the DFT-computed energies and compared to experimental measurements to assess the modelling results. Although sizeable differences between computed and experimental enthalpies and entropies are expected, leading to large deviations between Gibbs free energies, good agreement was found for the covalent capture of CO_2_ by the tetramers (Fig. 2e and Supplementary Fig. 44).

Computed enthalpies of CO_2_ absorption by tetramers at low loading (*α* = 0.07–0.22) perfectly match values derived from prior vapour–liquid equilibrium measurements on EEMPA (–79 versus –75 kJ mol^–1^)^22^, further supporting our tetrameric model (Supplementary Figs. 32 and 41). The computed entropies of CO_2_ capture by the tetramers (Supplementary Figs. 33 and 42) match the order of magnitude of values of aqueous amines^44,45^. With the exception of the first capture step (–200 J mol^–1^ K^–1^), these entropies are relatively constant along the loading process (~150 J mol^–1^ K^–1^). This agrees with the first capture reaction of the gaseous reactant being accompanied by the tetramerization of **E**, while subsequent absorption steps involve only the loss of translational and rotational freedom of the gaseous reactant. We experienced the limits of the DFT method while assessing the entropy of all non-covalent pairing processes (2**E** → **E**^**2**^ and 2**E**^**2**^ → **E**^**4**^; Supplementary Figs. 33, 36 and 39). Though the calculations confirmed that higher aggregates are systematically enthalpically favoured (Supplementary Figs. 32, 35 and 38) regardless of loading, enthalpy values were compensated for by overestimating computed entropies, leading to low positive Gibbs free energies of tetramerization^46^. This predicted endergonicity for dimerization and tetramerization (Supplementary Figs. 34, 37 and 40) is imputed to the limitation inherent to the solvent model. In fact, classical molecular dynamics simulations (1 μS trajectory) of the **E**/CO_2_ system at 298 K and 0.25 loading showed the coexistence of the **E**^**4**^**(0.25)** and **E**^**4**^**(0.50)** tetramers (defined by dominant hydrogen bonds; Supplementary Fig. 46) in proportions (18% and 22% of the whole system, respectively) that qualitatively agree with the NMR-data-derived MATLAB model. Complementary evidence supporting the existence of CO_2_-rich tetramers such as **E**^**4**^**(0.75)** and **E**^**4**^**(1)** was provided by Fourier transform infrared and wide-angle X-ray scattering (WAXS) spectroscopies (vide supra). Static DFT calculations confirmed that non-covalent clustering acts as a genuine thermodynamic driving force, stabilizing covalent adducts enthalpically by around 15 kJ mol^–1^ compared to the isolated species (Supplementary Figs. 32 and 35). Calculations revealed that combining unloaded **E(0)**, partially loaded **E(0)**^**+**^**E(1)**^**–**^ and fully loaded **E(1)** into clusters opens the door to a broad range of absorption enthalpies during CO_2_ capture (with values decreasing between from –80 to –20 kJ mol^–1^ along the **E**^**4**^**(0)**–**E**^**4**^**(0.75)** series; Fig. 2e), far beyond those observed on solid absorbents^9^. Consequently, clustering empowered by water-lean solvents may allow chemists to choose the thermodynamic features of the capture and release cycle on demand by setting the loading range, and thus selecting the active tetrameric species.

### Structural analysis of the E^4^ clusters

**Fig. 3:**
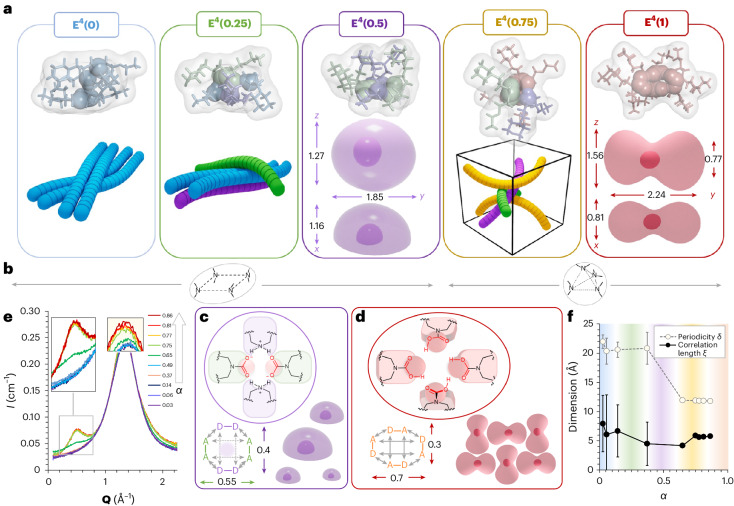
Structural analysis of the E^4^ tetrameric clusters and of their packing in solution. **a**, DFT-derived van der Waals surface of dominant tetramers and schematic tertiary structure (distances in nm). **b**–**d**, Simplified developed representation of the internal nitrogen-based tetrads (**b**) and of their hydrogen-bond network and tetramer packing modes (**c** and **d**; distances in nm; A, hydrogen-bond acceptor; D, hydrogen-bond donor). **e**, Stack of WAXS spectra recorded at increasing loading. **Q**, scattering vector; *I*, differential scattering cross-section per unit volume. Insets show zoomed-in views. **f**, Evolution of periodicity *δ* and correlation length *ξ* with loading. Data are presented as mean values. Details on error bar calculation are in the Supplementary Methods. Source data

**Fig. 4:**
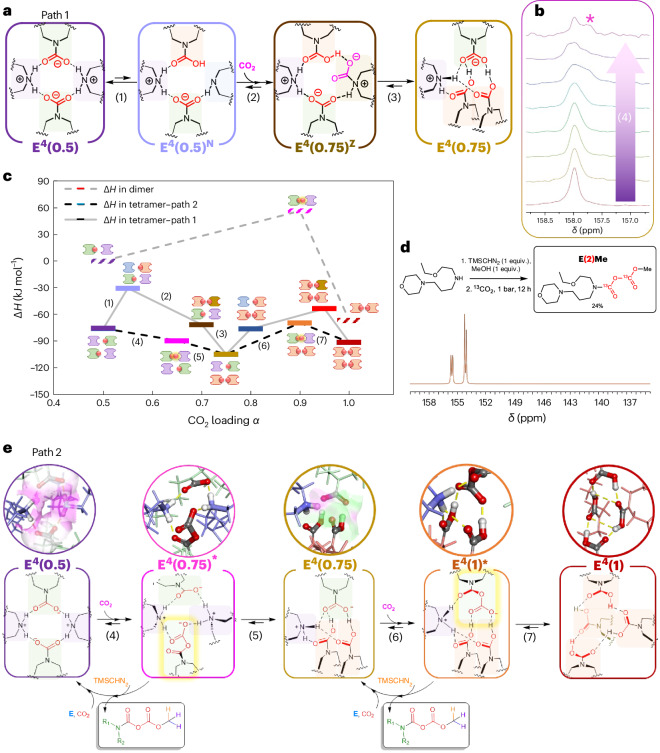
Mechanism of carbamic acid formation involving anhydride intermediate within E^4^ clusters. **a**, Zwitterion-mediated hypothetical carbamate to carbamic acid conversion pathway (path 1, steps 1–3; **E**^**4**^**(0.5)**^**N**^ and **E**^**4**^**(0.75)**^**Z**^ are neutral and zwitterionic intermediates, respectively). **b**, The q^13^C NMR carbonyl signal evolution upon CO_2_ loading above ambient pressure (step 4, bottom to top; * marks the additional CO signal from the anhydride). **c**, DFT-derived enthalpies (Δ*H*) of **E**^**2**^ and **E**^**4**^ clusters involved in the zwitterion-mediated (path 1, steps 1–3) and anhydride-mediated (path 2, steps 4–5 and 6–7) pathways. **d**, TMSCHN_2_ derivatization of ^13^C-labelled **E(2)**^**–**^ into **E(2)Me** (schematic on top and snapshot of the carbonyl region of the ^13^C NMR spectrum on the bottom). **e**, Proposed mechanistic pathway. The asterisk marks a carbamic anhydride-containing intermediate.

Given the level of agreement with experimental data, DFT modelling could be exploited to gain insights about the non-covalent interactions (Figs. 3c,d and 4c) that govern self-assembly and the structural features of the tetrameric clusters (Figs. 3a and 4a and Supplementary Fig. 45). The self-assembly of subunits **E(0)**, **E(0)**^**+**^, **E(1)**^**–**^ and **E(1)** is driven by a network of hydrogen bonds between the amine, ammonium, carbamate and carbamic acid polar head groups that form the tetrad at the core of the re(active) site. Low-loading tetramers **E**^**4**^**(0)** and **E**^**4**^**(0.25)** result from packing unfolded **E(0)** (**E(0)**^**+**^ and **E(1)**^**–**^) into cylindrical bundle-like tertiary structures, where the basic moieties of the (re)active site are buried and poorly accessible. In **E**^**4**^**(0.5)**, the **E(0)**^**+**^ and **E(1)**^**–**^ subunit chains individually fold into turns, with the polyether moieties gathered in one hemisphere (Fig. 3a). As a result, the tertiary structure of **E**^**4**^**(0.5)** is a half ovoid, exposing the square planar hydrogen-bonded (re)active site (Fig. 3b,c). Incorporation of an additional CO_2_ molecule leads to dramatic structural changes, both locally and globally (Fig. 3a,d). While carbamic acid formation is accompanied by hydrogen bonding reorganization, the polyether chains remain folded into turns as in **E**^**4**^**(0.5)**. This induces a conformational change of the polar group tetrad from square planar to tetrahedral (Fig. 3b), affecting the orientation of the side chains. Consequently, **E**^**4**^**(0.75)** adopts a star-shaped tertiary structure with spaced side chains roughly pointing towards the vertices of a cube, shielding the active site from the solution. In **E**^**4**^**(1)**, gathered pairs of ether chains (experimentally confirmed by NMR; Supplementary Fig. 17) yield a flattened figure-eight global structure (Fig. 3a,d).

Fourier transform infrared spectroscopy experimentally confirmed the presence of EEMPA clusters at high loading. While the conversion of ammonium carbamates to carbamic acid dimers is generally accompanied by a marked increase in the stretching frequency of the carbonyl signal^47,48^, the observed decrease (Supplementary Fig. 28) agrees with reports about higher carboxylic aggregates (Supplementary Fig. 29)^13^. WAXS analysis provided a second set of experimental evidence for the tetrameric clusters and information about their morphological features in pressurized and unpressurized conditions (Fig. 3e). The **Q** region (where **Q** is the scattering vector) exhibits a CO_2_ loading-dependent structure factor fit using the Teubner–Strey model^49–51^. The model qualitatively describes the segregation between the polar reactive moieties and ethoxyethyl and morpholinopropane arms encountered in the tetramers particularly well. The presence of this polar core provides the required electron density contrast and enables the observation of molecular-scale phase segregation at a CO_2_ loading above 0.6. The lack of phase segregation at lower CO_2_ loading values may be ascribed to the solution composition, which includes several tetramers (**E**^**4**^**(0.25)** to **E**^**4**^**(0.75)**) with differing morphologies poorly adapted to regular and dense packing. WAXS was used to probe the dimensions of these micelle-like clusters and their polar cores. The measured cluster dimensions, including a tetrad correlation length (*ξ*) of 5.8 Å and periodicity between adjacent tetrads (*δ*) of ~11.8 Å, quantitatively match with the DFT results (Fig. 3f and Supplementary Figs. 30, 31 and 45). The WAXS analysis also shows a cubic bicontinuous phase based on the head-to-hip packing of CO_2_-saturated tetramers (Fig. 3c,d). The WAXS data provide additional experimental evidence for the formation of tetramers, agreeing with the NMR-based speciation and DFT modelling.

### Anhydride-based mechanism of CO_2_ capture

The molecular mechanism leading to the formation of carbamic acid-containing clusters was explored by a coupled experimental (NMR of neat pressurized samples) and theoretical (DFT calculations) approach. In the classical zwitterion model, the first CO_2_ addition proceeds via a carbamic acid intermediate, which converts into ammonium carbamate upon deprotonation by a second amine^52^. In this framework, carbamic acid can be produced only from a neutral amine precursor. In our system, carbamic acid-containing species **E**^**4**^**(0.75)** and **E**^**4**^**(1)** arise from **E**^**4**^**(0.5)** via a stepwise CO_2_ absorption. We expected that forming carbamic acid from the **E**^**4**^**(0.5)** ammonium carbamate tetrad would first require an energetically uphill proton transfer from the nitrogen atom of one ammonium group to a neighbouring carbamate oxygen (Fig. 4a, step 1). The free amine centre of the unstable intermediate **E**^**4**^(0.5)* would then bind to a third CO_2_ molecule (Fig. 4a, step 2), generating the zwitterion **E**^**4**^**(0.75)**^**z**^ that would relax into **E**^**4**^**(0.75)** (Fig. 4a, step 3).

We exploited the viscosity increase that accompanies CO_2_ uptake by **E**^**4**^**(0.5)** to slow the decay of the elusive intermediates involved in the formation of **E**^**4**^**(0.75)** and **E**^**4**^**(1)**. In practice, an EEMPA sample was overpressurized with CO_2_ (*α* = 0.8–1 range) until equilibrium was attained and then transferred into an NMR tube with a headspace under 1 bar of CO_2_. The quantitative ^13^C (q^13^C) NMR spectra were immediately recorded over time once the transfer was complete. The monitored phenomenon is governed by slow mass transfer within the viscous medium. It corresponds to the stripping of **E**^**4**^**(0.75)** back into **E**^**4**^**(0.5)** and gaseous CO_2_ through the intermediate(s) species. Examining the stack of spectra recorded over time in reverse order (from the end to the beginning of the experiment; Fig. 4b, bottom to top) provides a sequence of snapshots of the intermediate states encountered during the **E**^**4**^**(0.5)** + CO_2_ → **E**^**4**^**(0.75)** absorption step (step 4; Fig. 4c,e). While the NMR signals of the aliphatic backbone for both **E(0)**^**(+)**^ and **E(1)**^**(–)**^ match those recorded at the same loadings at equilibrium (Supplementary Figs. 47 and 48 versus Supplementary Figs. 12–15), the carbamate signal displays a shouldered peak at high loadings (Fig. 4b and Supplementary Fig. 49). Relative integration of the signals corresponding to CO_2_-bearing versus CO_2_-free species in this loading range indicated that the CO_2_-bearing species is bound to more than one CO_2_ molecule on average.

To identify this carbamate-like intermediate species, we attempted in situ trapping via alkylation with trimethylsilyldiazomethane (TMSCHN_2_), previously employed for carbamate to urethane conversions^53^. To our surprise, traces of the methyl carbamic anhydride of EEMPA, notated as **E(2)Me**, could be directly detected by ^13^C NMR in the crude mixture (with 0.5 equiv. TMSCHN_2_; Supplementary Figs. 52 and 53). To confirm this observation and isolate the intermediate, CO_2_-free EEMPA was premixed with 1 equiv. TMSCHN_2_ and pressurized with CO_2_. Remarkably, **E(2)Me** formed at up to 37% conversion from the crude mixture. An analytically pure (99.5%) sample was recovered in a 24% isolated yield after column chromatography and characterized by mass spectrometry (Supplementary Figs. 63–65) and NMR spectroscopy (Fig. 4c and Supplementary Figs. 54–57). To further confirm the structure of this intermediate, the analogue **E(2)**^**t**^**Bu** (^*t*^Bu, *tert*-butyl) was synthesized ex situ from **E** with di-*tert*-butyldicarbonate^54–57^. Thus, we could compare the ^13^C NMR pattern of the intermediate trapped in situ with the spectra of these derivatives and unambiguously confirm the identity of **E(2)Me**. The most characteristic feature is the set of two doublets around 150 ppm in ^13^C NMR observed for both **E(2)Me** and **E(2)**^**t**^**Bu** (Supplementary Figs. 55, 58, 61 and 66). This pattern is indicative of a non-symmetrical bis(carbonyl) system split given the bulkiness of the capping end group (^*t*^Bu versus Me), explaining the slight difference in splitting patterns observed between the adducts (Fig. 4d).

DFT calculations confirmed that the anhydride intermediate is strongly stabilized within the reactive site of the tetrameric reverse-micelle-like clusters and favoured over the conventional zwitterion intermediate (Fig. 4a,c,e; Supplementary Figs. 50 and 51 for more detail). The enthalpic cost of proton transfer from the ammonium carbamate to the amine carbamic acid is rather high (Fig. 4a,c, step 1; more than 45 kJ mol^–1^), whereas the anhydride pathway follows a downhill energetic trajectory (Fig. 4c,e, step 4; –14 kJ mol^–1^) from **E**^**4**^**(0.5)**. The unique stereoelectronic features of the tetrameric cluster obviously favour this alternative reaction pathway, as carbamic anhydride formation from a traditional ammonium carbamate dimer such a **E**^**2**^**(0.5)** is thermodynamically strongly disfavoured (by +56 kJ mol^–1^). Electrostatic potential surface mapping (Fig. 4e) of the internal cavity of **E**^**4**^**(0.5)** additionally reveals an electron-poor cleft reminiscent of hydrolases’s oxyanion hole^58,59^. In these confined sites, poorly reactive moieties (alcohols and amides) are activated by a network of hydrogen bonds, which also stabilizes the electron-rich intermediates formed. The same phenomenon is believed to be at work here, to both activate the nucleophilicity of the carbamate and the electrophilicity of CO_2_ and stabilize the carbamic anhydride. Experimentally, this intermediate species instantaneously forms at room temperature. The rate-limiting step is its conversion into the carbamic acid-containing tetrad **E**^**4**^**(0.75)**. Hydrogen-bond pairing between the members of the **E(1)E(1)**^**–**^**E(1)E(0)**^**+**^ tetrad of **E**^**4**^**(0.75)** seems particularly efficient, as the resulting electrostatic potential surface is much less electrodeficient than in **E**^**4**^**(0.5)**. This complementarity is perturbed in the carbamic anhydride-containing intermediate tetrad **E**^**4**^(1)*. As a result, the fourth CO_2_ uptake is both kinetically and thermodynamically less favourable than the third (–14 versus –9 kJ mol^–1^). Remarkably, TMSCHN_2_ can selectively methylate and abstract the anhydride from this highly complex system as well as displace the cascade of reversible CO_2_ absorption events (Fig. 4c,e). This assertion is supported by the fact that premixing TMSCHN_2_ and unloaded EEMPA leads to the formation of substantial amounts of **E(2)Me** upon exposure to CO_2_ in standard temperature and pressure conditions. With this rather simple reactive system, two CO_2_ molecules can be linearly bound to a single nitrogen centre, doubling the amount of greenhouse gas that may be captured or sequestered into a carbon-based material. In agreement with recent reports^60,61^, the two urethane analogues **E(2)Me** and **E(2)**^**t**^**Bu** display moderate stability (conversion into **E(1)R** within days at room temperature and pressure). Continued studies of structure–reactivity relationships and stability are underway, where we anticipate making the next generation of CO_2_-rich urethanes with increased robustness. Ultimately, the reactive system identified here may have unlocked the principles needed to oligomerize and store several carbon dioxide molecules on the same backbone. If the recently discovered pyrocarbonate is the strict carbon analogue of pyrophosphate, anhydride **E(2)**^**–**^ can be viewed as an analogue of ADP, one of the universal molecules used to transport and store energy by nature. Capture within a clustered water-lean solvent may provide a conceptual framework to build a sustainable carbon value chain inspired by cellular metabolism, an intriguing approach to effectively mitigating CO_2_ emissions.

## Conclusion

The quest for an ideal CO_2_ capture solvent has spanned almost a century, beginning with patenting the amine scrubbing process and intensifying with the threat of global warming^62^. Single-component water-lean solvents have recently emerged as promising, displaying high energy efficiencies and low operational costs. However, it has been challenging to determine what molecular and mechanistic features give rise to the advantageous properties and performance of EEMPA and its analogues. It appears that combining a central basic secondary amine site with two flexible, mildly polar side chains enables EEMPA to behave as a proto-surfactant, forming micelle-like ionic clusters after CO_2_ binding. These stable supramolecular aggregates not only explain EEMPA’s unusual physical properties (low viscosity and high conductivity) but also enable a different covalent capture chemistry to take place within shielded and well-structured nano-environments. These cavities are highly reminiscent of enzymatic active sites, with structural and physical features that stabilize intermediates and catalyse numerous reactions in exceptionally mild conditions. A ‘double-tailed surfactant’-like secondary amine backbone seems to be a general prerequisite for self-assembly into reverse micelle clusters among water-lean solvents and opens the door to exciting reactivity, such as carbamic anhydride formation. We have gathered preliminary evidence of self-assembly and anhydride formation for a series of secondary–tertiary diamine analogues of EEMPA. The reactivity of EEMPA and CO_2_ can be conceptually depicted using a dynamic combinatorial framework^63^. Simple constituents such as **E**, CO_2_ and protons reversibly and covalently connect to yield a collection of capture products (**E(0)**, **E(0)**^**+**^, **E(1)**, **E(1)**^**–**^), themselves the components of higher tetrameric aggregates. These original proto-protein architectures may enable a shift beyond the canonical mono- and bimolecular capture adducts, in terms of both thermodynamics and kinetics. Tetramerization opens the door for stepwise CO_2_ capture, with each step displaying highly contrasting binding constants and absorption enthalpies beyond the values obtained with aqueous and metal–organic framework-appended absorbents. While EEMPA displays negative cooperativity, we believe it is just an example in a series towards positively cooperative liquid absorbents. Kinetically speaking, the shielded and hydrogen-bonded reactive core enables the formation of intriguing intermediates such as the CO_2_-enriched anhydride **E(2)**^**–**^. The way these pseudo-active sites modulate reactivity is illustrated by the alkylation process, which selectively proceeds on the unusual anhydride with respect to the carbamate. The spontaneous clustering of water-lean solvents dramatically expands the scope of thermodynamic and kinetic capture features but may serve as an original medium for the integrated transformation of unexpected capture intermediates such as **E(2)**^**–**^ into different end products.

CO_2_ anhydrides represent an emerging class of CO_2_ storage products, with higher mass content and similarities to the phosphate-rich biomolecules that control cellular energy storage. A next step is to extend the proof of feasibility to higher CO_2_ content and obtain an adenosine triphosphate analogue. This would pave the way towards a CO_2_-only oligomerization process, with solvent acting as an initiator. Ultimately, these structures provide a wealth of valuable information on CO_2_ reactivity in an unconventional, yet simple, medium. This may be the base for the next generation of impactful carbon capture utilization and storage technologies.

## Methods

All reagents were purchased from commercial sources and used as received. Regular liquid-state NMR spectra were recorded on either a 500 MHz Varian iNOVA, 500 MHz Bruker Avance NEO or 600 MHz Bruker NEO equipped with the Cryo Probe using standard pulse sequences. High-pressure liquid-state NMR spectra were recorded on a 500 MHz Varian iNOVA spectrometer, using in-house manufactured PEEK NMR tubes connected to a commercial Parr reactor and Teledyne ISCO pump. The ^13^C magic angle spinning NMR spectra were collected on a 600 MHz Bruker Avance III spectrometer using 5 mm zirconia rotors spinning at 3–5 kHz, a home-built custom HX probe and an in-house-developed WHiMS rotor system. Mass spectrometry analysis was performed using a Q Exactive Plus mass spectrometer (Thermo Scientific) outfitted with a heated electrospray ionization source and on a time-of-flight secondary ion mass spectrometer (ToF-SIMS 5, IONTOF). High- and low-pressure infrared spectra were collected on Nicolet iS10 Thermo Scientific instrument using a high-pressure demountable transmission liquid Harrick cell and OMNIC 9 software. WAXS experiments were carried out on a Xenocs Xeuss 2.0 small-angle X-ray scattering (SAXS)/WAXS system employing a monochromated Cu Kα (average wavelength *λ*_avg_ = 1.54189 Å) source and an effective *Q* range of 0.1–2.3 Å^–1^. Electron paramagnetic resonance measurements were performed on a Bruker ELEXSYS E580 spectrometer equipped with an SHQE resonator.

The Supplementary Information contains details of syntheses, spectroscopic analyses and computational studies.

## Online content

Any methods, additional references, Nature Portfolio reporting summaries, source data, extended data, supplementary information, acknowledgements, peer review information; details of author contributions and competing interests; and statements of data and code availability are available at 10.1038/s41557-024-01495-z.

### Supplementary information

Supplementary Information Supplementary Figs. 1–71, Tables 1–11 and Methods.

Supplementary Code 1 MATLAB code for dimeric model.

Supplementary Code 2 MATLAB code for tetrameric model.

Supplementary Data 1 Raw data for MATLAB fitting.

Supplementary Data 2 DFT optimized structures of all compounds.

Supplementary Data 3 Computed energies of all compounds studied, used as source data for Fig. 4c.

Supplementary Data 4 Animation showing the 1 μs classical molecular dynamics trajectory of **E**^**4**^**(0.25)** and **E**^**4**^**(0.5)** clusters in a 25 mol% CO_2_ loading system.

### Source data

Source Data Fig. 2 Source data for Fig. 2.

Source Data Fig. 3 Source data for Fig. 3.

## Data Availability

The data supporting the findings of this study are available within the paper and its Supplementary Information and data files. Should any raw data files be needed in another format, they are available from the corresponding authors upon reasonable request. Source data are provided with this paper.

## Code availability

The MATLAB script as well as the dataset used for the parametric fitting are available as supplementary data files. They have been uploaded to and are accessible from Zenodo at 10.5281/zenodo.10649310 (ref. ^64^).
