# Supplementary material for: Tetrameric self-assembling of water-lean solvents enables carbamate anhydride-based CO2 capture chemistry
Source: Nat Chem. 2024 Apr 8;16(7):1160–8. doi: 10.1038/s41557-024-01495-z (PMC11230897; doi:10.1038/s41557-024-01495-z)
Supplement: Supplementary file 1 — Supplementary Figs. 1–71, Tables 1–11 and Methods. [file 41557_2024_1495_MOESM1_ESM.pdf]

nature chemistry

Article https://doi.org/10.1038/s41557-024-01495-z

Tetrameric self-assembling of water-lean solvents enables carbamate anhydride-based CO_2 capture chemistry

In the format provided by the authors and unedited

Nano-Clustering in Water-Lean Solvents Establishes Novel CO_2 Chemistry

Table of content

Materials and Methods 2
Materials 2
Instruments 2
Synthetic Procedures and characterization 4
Analytical procedures 7
PVT Measurements 7
Polarity measurement 8
Loading assessment and EEMPA-CO_2 covalent adduct detection/quantification 9
Matlab data Analysis 12
Computational procedures 15
DFT Calculations of the stability of the different clusters 15
Static DFT calculations of the rotamer energy landscape on **E(2)R** species 15
cMD modelling of Tetrameric Structures as a function of time at 25% CO_2 loading 15
WAXS procedures and data analysis 17
Supplementary figures 19
pKa determination 19
Gas-liquid partition 20
Polarity measurements 24
Binding isotherms 25
NMR speciation 27
^{13}C-NMR spectra of the carbamic acid **E(1)** 33
Fitted NMR data 37
Speciation models 41
FT-IR data 43
DFT-computed clustering and absorption energies 47
Structural data from DFT calculations 60
cMD data for Tetrameric Structures as a function of time at 25% CO_2 loading 61
^{13}C NMR monitoring of E(2) decay 63
DFT analysis of CO_2 absorption mechanisms from 50% loading state 66
NMR and MS data of in situ and ex situ **E(2)R** formation 68
DFT-computed conformational energies on **E(2)R** derivatives 84
Supplementary Tables 86
Abbreviations and schematic representations 86
Binding isotherms and speciation data 89
WAXS parameters 91
References 93

Materials and Methods

Materials

Trimethylsilyldiazomethane in hexanes was purchased from Thermo Fisher and used without further purification. $^{13}CO_2$ (99%) was purchased from Cambridge isotopes. Boc_2O and DMAP were purchased from Sigma-Aldrich and used without purification.

Instruments

Mass Spectrometry analysis was performed using an Q Exactive Plus mass spectrometer (Thermo Scientific, San Jose, CA) outfitted with a Heated ESI source. The source temperature, ion transfer tube temperature and spray voltage were 110ºC, 300ºC and 3.9 kV, respectively. Data weas collected for 2.5 min, with data acquisition being initiated immediately a stable signal is observed. FT-MS spectra was acquired from 50 to 650 m/z at a resolution of 140k (AGC target 3e6).

Negative mode Cryo ToF-SIMS analysis was performed on ToF-SIMS 5 (IONTOF, Műnster, Germany) using 10 kHz 100 µs cycle time and flood gun of 2.10 Å. Temperature of the experiment was set to -128 °C and raised to -115 °C and then to -80 °C. Time of the experiment was 30 min for the first raise and 45 min. for the second temperature raise. During first step, a 2 mm spacer was used in the cell, the during second step no spacer was inserted. Prior to analysis, sample was frozen in liquid nitrogen and mounted on a cooling stage.

High and low pressure IR spectra were collected on Nicolet iS10 Thermo Scientific instrument using high pressure demountable transmission liquid Harrick cell, and OMNIC 9 software.

Regular liquid state NMR spectra were recorded on either 500 MHz Varian iNOVA, 500 MHz Bruker Avance NEO or 600 MHz Bruker NEO equipped with the Cryo Probe using standard pulse sequences. Spectra in Supplementary 24-61 were collected using $CDCl_3$ and referenced to residual solvent signal ($CHCl_3$ at 7.26 for ^1H and 77.16 ppm for ^{13}C NMR). Spectra in Supplementary Fig.52-53 were collected neat and were referenced to methyl signal of 2-EEMPA set to 16.1 ppm.

High-pressure liquid state NMR. All spectra were recorded on 500 MHz Varian iNOVA spectrometer, using in-house manufactured PEEK NMR tubes connected to commercial Parr reactor and Teledyne ISCO pump. All experiments were recorded using standard pulse sequences with delay time d_1 = 5 or 10 seconds. Spectra were recorded without use of additional solvent and referenced to acetonitrile as an external reference

Solid-state NMR ^{13}C MAS-NMR spectra were collected on a 600 MHz Bruker Avance III spectrometer using 5 mm zirconia rotors spinning at 3–5 kHz. We utilized a home-built custom HX probe as described previously where the design is compatible with our in-house-developed WHiMS rotor system, additionally, (1)magnetic susceptibility matched wire for the RF coil further enhances the spectral resolution of the mixed-phase samples. The 90° pulse width calibrated for the probe was 5 µs for both ^1H and ^{13}C, and the relaxation delay was optimized for full relaxation before each scan. 1H and 13C spin-lattice relaxation time (T_1) measurements utilized the inversion-recovery (180° - t_{delay} - 90° - acquisition) sequence. ^1H-^1H and ^{13}C-^{13}C exchange spectroscopy (EXSY) experiments using a standard pulse sequence were carried out with several mixing times (5, 50, and 100 ms).

WAXS Measurements. Wide-angle x-ray scattering experiments were carried out on a Xenocs Xeuss 2.0 SAXS/WAXS system employing a monochromated Cu K_{α} (λ_{avg}= 1.54189 Å) source and an effective Q range of 0.1- 2.3 $Å^{-1}$. The sample pressure was controlled using a manual piston pump and in-house high pressure tubing manifold connected to a 1 mm inner diameter polyimide tubing. Each scattering run was collected for 5 minutes using beam dimensions of 0.6 mm x 1.0 mm (horizontal × vertical) at the sample. The two-dimensional scattering pattern was collected on a Dectris Pilatus 200k detector with 0.172 mm pixel size, and the azimuthally symmetric pattern was averaged into one-dimensional curves for further background subtraction and scaling to absolute units of differential scattering cross section per unit volume.

Potentiometric Titration was performed using a Mettler Toledo T5 instrument with a DGi102-Mini glass electrode calibrated with standard calibration solution at pH = 4.01; 7.01 and 9.00 purchased from Mettler Toledo.

Solvatochromatic Analysis. Solvatochromic measurements were performed using Nile red (Sigma-Aldrich). Nile red dye was dissolved in the solvent to produce a concentration of 10^{-4} M. Absorbance was measured with an Agilent Cary 60 UV-Vis spectrophotometer at room temperature.

EPR analyses. All EPR measurements were performed on a Bruker ELEXSYS E580 spectrometer equipped with an SHQE resonator. A quartz capillary, ID 0.8 mm and OD 1 mm, was used to hold the solution in the EPR cavity with both ends sealed by Critoseal. Instrument settings used to record the spectra were microwave frequency = 9.32 GHz, sweep width = 50 G, sweep time = 42s, microwave power = 2 mW, field modulation amplitude = 0.1 G, with four scans averaged. Spin Hamiltonian parameters for the spectra were obtained by fitting with EasySpin.[1]

Synthetic Procedures and characterization

EEMPA $\boldsymbol{E_0}$

Synthesis. N-(2-ethoxyethyl)-3-morpholinopropan-1-amine (2-EEMPA, **E**) was synthesized using the synthetic methodology developed in our previous work[2] and was successfully characterized using proton ^{1}H and ^{13}C carbon NMR spectroscopy. The reaction was carried out under nitrogen atmosphere. All the reagents were purchased from Sigma Aldrich, TCI and Fisher scientific and were used without purification.

Assessment of Water content in EEMPA. Coulometric Karl Fischer titrator C30S was used to measure the water content in synthetic batches of 2-EEMPA. Each batch had water content analysis performed in triplicate, with contents of all batches ranging between 309 ppm to 580 ppm H_2O.

pKa Measurement by potentiometric titration. A 0.37 M solution of EEMPA was prepared by diluting 1,47 g of EEMPA in 20 mL of triply distilled water. A 0.5 M HCl solution was freshly prepared by dilution of 37% HCl solution with triply distilled water. 10 mL of the 0.37 M EEMPA sample was added into a 50 mL glass-jacketed titration cell containing a Teflon-coated stirrer. Aliquots (0.2 mL) of 0.5 M HCl solution were then added to the sample and the pH was monitored. The $[H^+]$ of the solutions was determined by the measurement of the electromotive force of the cell, $E=E_0 + Q \log[H^+]$. The term pH is defined as $-\log[H^+]$. E_0 and Q were determined by calibration of the electrode using a solution of known hydrogen-ion concentration at pH= 4.00; 7.01 and 9.00. Two equivalent points were detected during the titration (**Fig S1a**) of **E(0)** by HCl, corresponding to the sequential formation of $\mathbf{E(0)^+}$and $\mathbf{E(0)^{2+}}$ (**Scheme S1**).

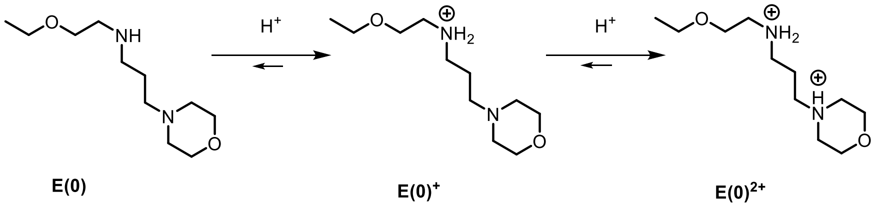

Supplementary Fig 1. sequential protonation reactions on EEMPA.

The Bjerrum function ($\bar{n}$) detailed in (eq 1) was used for accurate pK_a determination. This function quantifies the average protonation state per unit of analyte concentration [3], as :

$$\bar{n} = \frac{Total\ bound\ proton}{Total\ analyte} = \frac{c_{H+} - [H^+]}{c_t} \quad (1)$$

Where c_{H+} and c_t are the total concentrations of the proton and analyte, respectively. The difference between the total and free hydrogen ion concentration corresponds to the concentration in bound hydrogen ions, and it can be obtained by measuring the pH of the solution and computing the total

hydrogen ion concentration. pK_a values were extracted from the inflexion points on the corresponding curve (**Supplementary Fig.1b**).

pK_a(**E(0)/E(0)**$^+$) = 9.25 and pK_a(**E(0)**$^+$/ **E(0)**$^{2+}$) = 6.01

In situ Synthesis of ***E(2)Me.***

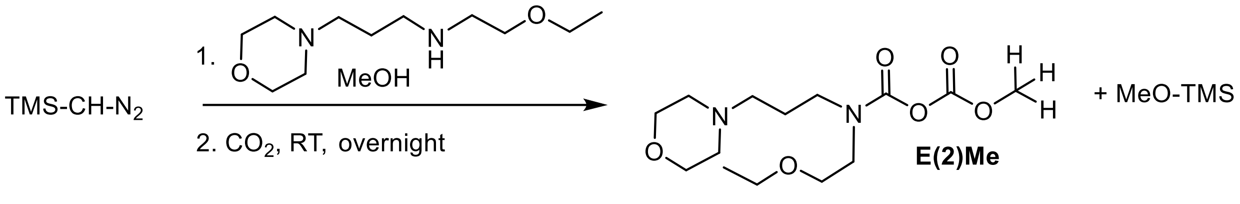

10 mL of ~0.6 M trimethylsilyldiazomethane in hexanes (corresponding to 0.685 g, $6\cdot10^{-3}$ mol) was transferred into an Ace pressure tube with a plunger and a front seal and put under vacuum until more than 90% of hexanes has been removed (at this point, TMS-diazomethane started to evaporate as well, as evidenced by yellow color of the condensate in the trap). Next, 1.3 g ($6\cdot10^{-3}$ mol) of 2-EEMPA (and, in some cases, 0.24 mL (0.192 g, $6\cdot10^{-3}$ mol) of MeOH) were added. This mixture was freeze-pump-thawed twice to remove any residual air. Next, $^{13}CO_2$ (or $^{12}CO_2$) was introduced to the mixture, which was briefly cooled down to condense as much gas as possible. Reaction was left stirring overnight. After this time, reaction was concentrated and when specified, an aliquot was taken out and analyzed by NMR (Supplementary Fig.52 and 53) and ToF-SIMS mass spectrometry (Supplementary Fig.64).

Crude material was purified on column eluting with Hex:AcOEt 1:1 and then 4:1. 456 mg (23.7 % yield) of a pale yellow liquid of 99.5% purity were obtained.

Characterization : NMR

^{1}H NMR ($CDCl_3$, 298 K, 500 MHz): δ 3.67 (t, J = 4.9 Hz, 4H); 3.64 (t, J=3.5 Hz 3H); 3.52-3.48 (br, 2H); 3.44 (dd, J = 6.5 Hz, J = 7.0 Hz, 2H); 3.40-3.32 (br, 2H); 3.29 (br, 2H); 2.39 (br, 4H); 2.29 (br, 2H); 1.14 (t, 3 J = 7.0 Hz, 3H). ^{13}C NMR ($CDCl_3$, 298 K, 125 MHz): δ 157.5 (1C, N$\underline{C}$(O)O); 156.8 (1C, O$\underline{C}$(O)O); 69.0 (1C, $\underline{C}OCH_2CH_3$); 67.0 (2C, O$\underline{C}H_2$ morpholine); 66.5 (1C, O$\underline{C}H_2CH_3$); 56.1 (1C, $N_{morpholine}\underline{C}H_2$); 53.7 (2C, N$\underline{C}H_2$ morpholine); 52.5 (O$\underline{C}H_3$); 47.5 (1C, N$\underline{C}H_2CH_2OCH_3$); 46.7 (1C, $N_{morpholine}CH_2CH_2\underline{C}H_2$); 25.4 (1C, $N_{morpholine}CH_2\underline{C}H_2$); 15.2 (1C, $OCH_2\underline{C}H_3$). BR-SIMS m/z calcd for $C_{14}H_{24}N_2O_6$ [M-H]- 317.2, found 317.2. Calcd for for $^{13}C_2C_{12}H_{24}N_2O_6$ [M-H]- 319.18, found 319.18ESI-FTMS m/z calcd for $C_{14}H_{26}N_2O_6$ [M+H]+ 319.1869, found 319.1840. Calcd for $^{13}C_2C_{11}H_{24}N_2O_5$ [M-OCH_3+H]$^+$ 290.18, found 290.18

Stage	CO_2	MeOH (molar eq)	NMR	MS

Purified	^{12}C	0	Fig S58-59	Fig S63
Crude	^{12}C	0	N.A.	Fig S62
Crude	^{13}C	0	Fig 52-53	Fig S64
Purified	^{13}C	0	Fig S54-57 & Fig S60-61	N.A.
Purified	^{13}C	1	Fig S60-61	N.A.

Ex situ Synthesis of reference compound ***E(2)tBu***

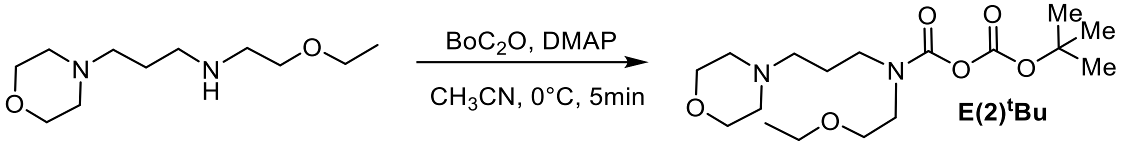

The procedure followed was adapted from previous report.[4] In an ice bath cooled round bottom flask under argon atmosphere, Boc_2O (2 equiv) was dissolved in 3 mL of MeCN, and DMAP (0.5 equiv) was added. After 5 min, a solution of EEMPA (0.5 mmol) in 2 mL of MeCN was added slowly during 2 min, and after an additional 1-5 min, chloroform (10 mL) was added and the solution was washed immediately with water (2 × 50 mL), dried with MgSO4, and evaporated to give a colorless oil.

^{1}H NMR (CD_3CN, 298 K, 500 MHz): δ 3.55 (t, br, 4H); 3.42-3.37 (m, 4H); 3.29 (br, 2H); 3.21 (t, 3J = 6.5 Hz, 2H); 2.31 (br, 4H); 2.23 (t, 3J = 6.75 Hz, 2H); 1.66 (m, br, 2H); 1.38 (s, 9H); 1.10 (t, 3 J = 7 Hz, 3H). ^{13}C NMR (CD_3CN, 298 K, 125 MHz): δ 155.7 (1C, N$\underline{C}$(O)O); 141.2 (1C, O$\underline{C}$(O)O); 78.3 (1C, O$\underline{C}(CH_3)_3$); 69.2 (1C, O$\underline{C}H_2CH_2$); 66.6 (2C, O$\underline{C}H_2$ morpholine); 65.8 (1C, $N_{morpholine}\underline{C}H_2$); 55.8 (1C, O$\underline{C}H_2CH_3$); 53.7 (2C, $\underline{C}H_2N_{morpholine}$); 47.3 (1C, $OCH_2\underline{C}H_2$); 46.1 (1C, N$\underline{C}H_2CH_2CH_2$); 28.2 (3C, OC($\underline{C}H_3)_3$); 25.3 (1C, $NCH_2\underline{C}H_2CH_2$); 15.1 (1C, $OCH_2\underline{C}H_3$). ESI-FTMS m/z calcd for $C_{17}H_{32}N_2O_6$ [M+H]+ 361.2333, found 361.2336.

Analytical procedures

PVT Measurements

To support and complement the NMR-based speciation, CO_2-binding isotherm was measured using a custom "Pressure Volume Temperature (PVT) cell." This apparatus was designed for simultaneous measurement of vapor liquid equilibrium, sorption kinetics and solvent viscosity[5] (**Scheme S2**) as described in our previous published work on EEMPA.[6] Vapor-Liquid Equilibrium measurements on CO_2 sorption were collected on the static synthetic method[7] which known amounts of CO_2 are injected into the cell of known volume and allowed to come to equilibrium pressure at a fixed temperature. The PVT apparatus provides direct measurement of total pressure in the cell, however the equilibrium partial pressure of CO_2 (P*) is calculated by subtracting the vapor pressure of EEMPA from the total pressure equilibrated at a given temperature of the measurement. EEMPA's vapor pressure is ~1 mbar at 25 ˚C, resulting in the total equilibrium pressure P_f to be equivalent to P_f* of CO_2 (**Supplementary Fig.2**).

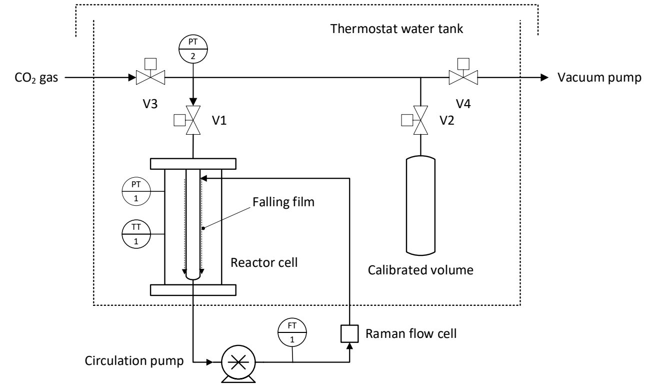

Supplementary Fig. 2. Schematic of the PVT apparatus used for isotherm measurements.

Polarity measurement

Solvatochromic Measurements and Analysis. Kamlet-Taft polarity parameters provide insight into a solvent's hydrogen bond donating ability or acidity (α), hydrogen bond accepting or basicity (β) and polarity-polarizability (π^*), based on the absorbance maximum exhibited by the solvatochromic dye. The polarity-polarizability scale of π^* is an indicator of a solvent's ability to stabilize charge or dipole moment.[8] Linear relationships between the dielectric constant with π^* include a contribution from the refractive index[8,9] which introduces an inherent difficulty to calculate the dielectric constant of an unknown solvent from π^* when the refractive index is also unknown. Similar attempts to correlate the Reichardt's parameter, $E_T(30)$, with the solvent dielectric constant have also been proposed with contradicting results.[10] More importantly, Reichardt's dye bleaches in amine solvents, limiting the ability to apply the $E_T(30)$ scale to CO_2 capture solvents like EEMPA. Alternatively, Nile Red dye was used to determine the polarity of the EEMPA solvent based on the λ_{max-}. Nile red dye was dissolved to produce a concentration of 10^{-4} M.

EPR Measurements and Spectroscopy. TEMPOL (4-Hydroxy-TEMPO, Sigma-Aldrich) was dissolved in all solvents to produce stock solutions of 10 mM which were subsequently diluted to produce 100 μM solutions. The samples were purged with helium to remove dissolved oxygen. All EPR measurements were performed on a Bruker ELEXSYS E580 spectrometer equipped with an SHQE resonator. A quartz capillary, ID 0.8 mm and OD 1 mm, was used to hold the solution in the EPR cavity with both ends sealed by Critoseal. Instrument settings used to record the spectra were microwave frequency = 9.32 GHz, sweep width = 50 G, sweep time = 42s, microwave power = 2 mW, field modulation amplitude = 0.1 G, with four scans averaged. Spin Hamiltonian parameters for the spectra were obtained by fitting with EasySpin [1].

Loading assessment and EEMPA-CO_2 covalent adduct detection/quantification

NMR samples preparation at 1 atm for EEMPA-CO_2 isotherms and speciation (Supplementary Fig. 5 and **12-15)**

All spectra were run neat and referenced to external capillary (^{1}H Proton to $C_3H_3(CF_3)_3$ at 8.5 ppm and ^{13}C Carbon to CD_3CN at 118.26 ppm). EEMPA samples containing varying EEMPA/ CO_2 ratios x_0 were obtained as previously described in the literature,[11,12] by combining pressure and gravimetric monitoring on tubes containing a known amount of EEMPA. The following parameters were used for spectra recording : D1 = 75 sec; # scans 512 except for free 2-EEMPA, which was 128.

NMR sample preparation above 1 atm for EEMPA-CO_2 isotherms and speciation (Supplementary Fig. 5 and **16-17).** The general methodology followed correspond to procedures validated in the literature.[12] For pressurized experiments, rotors of the WHiMS design were used.[13] This material allows for gas to be added via a one way check valve. The liquid portion of the sample was pipetted into a tared rotor which was then sealed with the o-ring containing bushing. After this reference mass was recorded, the rotor was placed in a pressure vessel. After removal of the surrounding air with a vacuum pump, the vessel (and therefore the rotor) was pressurized with CO_2 using an Isco syringe pump. After equilibration (~15 minutes), the rotor was removed and the new mass recorded. We utilized a home-built custom HX probe as described previously[14] where the design is compatible with our in-house-developed WHiMS rotor system, additionally, magnetic susceptibility matched wire for the RF coil further enhances the spectral resolution of the mixed-phase samples. The 90° pulse width calibrated for the probe was 5 μs for both ^{1}H and ^{13}C, and the relaxation delay was optimized for full relaxation before each scan. 1H and 13C spin-lattice relaxation time (T_1) measurements utilized the inversion-recovery (180° - t_{delay} - 90° - acquisition) sequence. ^{1}H-^{1}H and ^{13}C-^{13}C exchange spectroscopy (EXSY) experiments using a standard pulse sequence were carried out with several mixing times (5, 50, and 100 ms).

Qualitative monitoring of the equilibrium between dissolved CO_2 and EEMPA-bound CO_2 ($E(1)^{(-)}$) by MAS ^{13}C NMR. EEMPA was sparged with CO_2 gas at ambient pressure for 3 minutes and then pressurized for 1 minute around 20 bars (corresponding to an initial CO_2/EEMPA stoichiometry x_0 of 0.99). The sample was subjected to static and MAS ^{13}C and ^{1}H NMR (**Supplementary Fig. 20**). At low loading (α = 0.47), mixing of CO_2 and EEMPA happens quickly, so the spinning of the rotor simply offers higher resolution for the spectra. At higher loading (α = 0.75), the complete mixing and reaction of CO_2 and EEMPA takes more than 30 minutes, therefore the rotor spinning provides a fast complete mixing between CO_2 and EEMPA, as seen in the sudden conversion of **$E(1)^-$** (carbamate) into **E(1)** (carbamic acid) corresponding to the shielding of the ^{13}C CO signal by more than 1 ppm (see also **Supplementary Fig. 17**)

Qualitative monitoring of the anhydride E(2)$^-$ conversion into carbamic acid E(1) (Supplementary Fig. 47-49) 3 mL of 2-EEMPA was put in a 25 mL Parr reactor, which was closed and evacuated. Next, Parr reactor overhead stirrer was switched on and 2-EEMPA was pressurized with P^*_0 = 20 bars (x_0 = 0.99) of $^{12}CO_2$ delivered by high pressure Teledyne ISCO pump and kept at constant pressure by continuous CO_2 delivery. During pressurization, temperature increased from ambient to ~49 °C as CO_2 was being consumed. After temperature decreased back down and stabilized at 26 °C, stirring was switched off and the mixture was pushed with P^*_0 = 35 bars of CO_2 (x_0 = 1.6) through the PEEK tube into the PEEK NMR cell without opening it to air. Spectra were recorded immediately over a period of 19h.

Direct observation of E(2)$^-$ and of its capping by $TMCHN_2$ by ^{13}C NMR (Supplementary Fig. 52-53)

2.6 g (0.011 mol) of 2-EEMPA was mixed with 10 mL of ~0.6 M trimethylsilyldiazomethane in hexanes. This mixture was placed in an Ace pressure tube with a plunger and a front seal and put -26 inHg vacuum for 5 min.). Next, $^{13}CO_2$ was added to the mixture, which was immersed in liquid N_2 for 30 seconds to condense as much $^{13}CO_2$ as possible. Reaction was left stirring for 2 h. Upon warming up of the solution, it bubbles and releases excess of condensed $^{13}CO_2$. At this stage, the pressure flask was opened, a 0.5 mL aliquot was drawn and analyzed by ^{13}C NMR, confirming initial formation of the carbamate. Small doublet of doublets assigned to **E(2)Me** starts to be already visible as well (**Supplementary Fig.52**). After the NMR spectra were collected, the faint yellow sample of moderate viscosity (due to the presence of hexanes) was put back in the reaction vessel, which was briefly evacuated to to -26 inHg to remove headspace gas. The vessel was then back-filled with CO_2 and allowed to stand overnight. ^{13}C NMR analysis confirmed full disappearance of signal @ 161.2 ppm, which was attributed to the carbamate species. This disappearance is concurrent with growth of doublet of doublets, which we attribute to the **E(2)Me** species

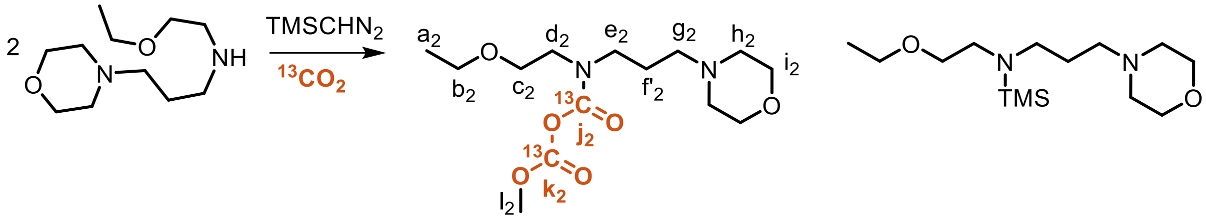

Pressurized Sample preparation and FT-IR spectra acquisition. A Nicolet is10 FT-IR equipped with a DTGS KBr detector coupled with a high-pressure demountable liquid cell was used to record transmission spectra. The spectrum of the empty transmission cell was used as background to remove residual signals of atmospheric water and CO_2. EEMPA was loaded into the transmission cell using a syringe to create a thin layer on the KBr windows. The cell was connected to a Parr reactor that was pressurized with CO_2. The transmission cell was initially evacuated before a two-way valve was opened pressurize the cell to 1328 psi. Pressure was

monitored using a pressure transducer provided by a Parr reactor controller that was connected to the Parr reactor. The pressure was held constant for 20 minutes before the pressure was released by opening a two-way valve to a second Parr reactor that was under vacuum. Spectra were recorded throughout the isobaric process and during the stepwise decrease in pressure. All spectra were recorded using 32 scans at a resolution of 4 cm^{-1}. Initial pressure P_0 to stoichiometry x_0 to loading a conversions were conducted using reference curves displayed at **Supplementary Fig. 5**.

Matlab data Analysis

Dimeric EEPMA model

$$xE_{0.5}^{2} = K_{0.5}^{2} \cdot xE_{0}^{2} \cdot xCO2 \quad (1)$$

$$xE_{1}^{2} = K_{1}^{2} \cdot xE_{0.5}^{2} \cdot xCO2 \quad (2)$$

$$xE_{0}^{2} + xE_{0.5}^{2} + xE_{1}^{2} = 1 \quad (3)$$

$$xCO2 = x_{0} - \alpha^{2} \quad (4)$$

With $xCO2$ being the molar fraction of unbound CO_2 (in the headspace and as a dissolved gas) and xE_i^2 the molar fraction of i-loaded dimer (i = 0, unloaded, i = 0.5 half-loaded, i= 1, fully loaded).

The chemical loading a of the sample can be expressed as :

$$\alpha^{2} = 0{,}5\, xE_{0.5}^{2} + xE_{1}^{2} \quad (5)$$

Tetramolecular EEPMA system:

$$xE_{0.25}^{4} = K_{0.25}^{4} \cdot xE_{0}^{4} \cdot xCO2 \quad (6)$$

$$xE_{0.5}^{4} = K_{0.5}^{4} \cdot xE_{0.25}^{4} \cdot xCO2 \quad (7)$$

$$xE_{0.75}^{4} = K_{0.75}^{4} \cdot xE_{0.5}^{4} \cdot xCO2 \quad (8)$$

$$xE_{1}^{4} = K_{1}^{4} \cdot xE_{0.75}^{4} \cdot xCO2 \quad (9)$$

$$xE_{0}^{4} + xE_{0.25}^{4} + xE_{0.5}^{4} + xE_{0.75}^{4} + xE_{1}^{4} = 1 \quad (10)$$

$$xCO2 = X_{0} - \alpha^{4} \quad (11)$$

With $xCO2$ the amount of unbound CO_2 and

$$\alpha^{4} = 0{,}25\, xE_{0.25}^{4} + 0{,}5\, xE_{0.5}^{4} + 0{,}75\, xE_{0.75}^{4} + xE_{1}^{4} \quad (12)$$

Equilibrium constant determination by parametric fitting.

The association constants for both dimeric and tetrameric EEMPA clusters are calculated through the concomitant fitting of the speciation (molar fraction of **E(0)**$^{(+)}$ and **E(1)**$^{(-)}$ species quantified by q^{13}C NMR as a function of X_0) using a MATLAB script with:

The CO_2 loading α, determined by q^{13}C NMR or PVT (2 x 49 dataset),

And,

The CO_2 loading α, determined by q^{13}C NMR and the chemical shift variation of each of the 18 ^{13}C nucleus observed, with respect to its value at $x_0 = 0$ (20 x 14 dataset),

For any given set of equilibrium constants, the molar fraction of each species of the system is determined by solving the system of equation composed of the CO_2 absorption equations and CO_2 / EEMPA mass balances (equations (1) to (5) for the dimeric system or equations (6) to (12) for the tetrameric system). During the fitting process, the predicted molar fractions are determined for any set of association constants and total quantity of CO_2 X_0 using MATLAB's build-in *fsolve* function with Levenberg-Marquart algorithm.

The predicted CO_2 loading α is obtained through the equation (5) or (12).

When appropriate, for each ^{13}C nucleus, the predicted chemical shift perturbation $\Delta\delta_i^n$ of nucleus i in the n-mer model is obtained through the equations (13) or (14), after parameter adjustments of the chemical shift perturbations $d\delta_i^n$ through curve fitting with chemical shift variation data, using MATLAB's build-in *lsqcurvefit* function with trust region reflective algorithm.

$$\Delta\delta_i^2 = d\delta_{0.5,i}^2 \cdot xE_{0.5}^2 + d\delta_{1,i}^2 \cdot xE_1^2 \quad (13)$$

$$\Delta\delta_i^4 = d\delta_{0.25,i}^4 \cdot xE_{0.25}^4 + d\delta_{0.5,i}^4 \cdot xE_{0.5}^4 + d\delta_{0.75,i}^4 \cdot xE_{0.75}^4 + d\delta_{1,i}^4 \cdot xE_1^4 \quad (14)$$

Comparison between predicted and observed data permits the parameter adjustment of the association constants using the *lsqcurvefit* function with trust region reflective algorithm. The fittings are performed on non-normalized datasets. CO_2 loading data receives a higher ponderation than chemical shift variation during the fitting to account for their higher reliability (coherent q^{13}C NMR and PVT data) and lower variability. The ponderation of α data was set at 10 against 1 for each ^{13}C nucleus chemical shift variation. Other ponderations were tested between 1 and 20 without significant change to the result To avoid local optimums, initial constant values are randomly generated within the design space over multiple attempts (>30) and fittings displaying the lower residual are conserved.

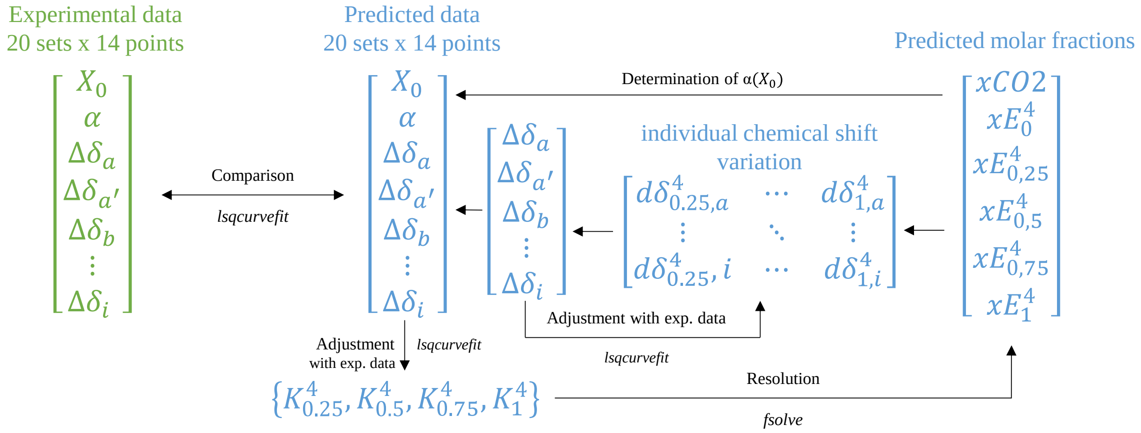

Supplementary Fig. 3.

Schematic representation of the algorithm used for the determination of association constants (here for fitting over CO_2 loading α and chemical shift variations, with the tetramolecular EEMPA system).

Computational procedures

DFT Calculations of the stability of the different clusters

Static DFT calculations were performed using ORCA software package[15,16] using B3LYP[17,18] functional and Grimme-D3 dispersion correction.[19] Def2-TZVP basis sets were used for all the atoms and SMD implicit solvent model[20] with DMF, t-butanol and 1-propanol solvents were used for structural optimizations. Numerical frequencies were computed to confirm that the structures obtained were local minima on the energy landscape and to assess their individual entropy. Based on standard thermochemistry, harmonic vibration frequencies[21,22] were computed numerically as implemented in ORCA to estimate the vibrational contributions to entropy. The enthalpy of each structure (H) was computed by summing electronic energy, zero-point energy, including thermal correction (vibrational, rotational, translational), and enthalpy correction (k_BT, where k_B is Boltzmann's constant and T is the general temperature for experiments, 298.15K). The entropy term (TS) was computed by summing the electronic, vibrational, rotational, and translational entropy. The Gibbs free energy (G) was obtained as the difference of both computed values H-TS. The energy landscapes followed by the rotamers of each alkylated anhydride were also assessed using this computing methodology. It should be noted that in these DFT calculations, the approximation of cluster models in the implicit solvent may overestimate the real condensed-phase free energy, as discussed in previous work.[21,23,24] However, such an approach is still widely employed based on the fair trade-off between accuracy and efficiency,[25–30] particularly in the self-assembly solvent systems.[31–35] Therefore, we chose in this work to focus on the qualitative insights into the free energy changes of our different structures. Additional quantification of free energies of these solvents using explicit solvent approaches is currently underway.

Static DFT calculations of the rotamer energy landscape on **E(2)R** species

The DFT calculations were carried out on **E(2)R** (R = H, Li, Me, ^{t}Bu) using SMD implicit solvent model. Several solvents were tests (among which those in which the NMR spectra were recorded) to probe impact of the dielectrics of the medium on energy barriers between **E(2)R** rotamers and on their stability. Several R substituents were explored (including those corresponding top compounds observed and/or isolated (R = H, Me, tBu) to similarly assess their impact on energy barriers between **E(2)R** rotamers and on their stability.

cMD modelling of Tetrameric Structures as a function of time at 25% CO_2 loading

We carried out classical molecular dynamics simulations, based on the all-atom OPLS force fields (OPLS-AA),[36] as implemented in the GROMACS 2022 program.[37] All the necessary force field parameters were generated using the LigParGen web server (http://zarbi.chem.yale.edu/ligpargen/),[38,39] except for the partial atomic charges. The charged were calculated using a quantum mechanical electrostatic potential fitting approach at the B3LYP/6-31G** level of theory as implemented in NWChem.[40] We adopted the particle-mesh Ewald (PME) scheme for electrostatics. The cut-offs for van der Waals and electrostatic interactions were set to 9.5 Å. The isothermal-isobaric (NPT) ensemble was used to obtain the liquid density at the ambient pressure and 298K. The canonical ensemble (NVT) was used to equilibrate the systems and obtain their statistics. In NPT simulations, the pressure and temperature

were controlled with a barostat (stochastic cell rescaling scheme [41] (5-ps time constant) and thermostat (v-rescale, 1-ps time constant), respectively. In NVT simulations, the temperature is maintained with the Nosé–Hoover thermostat (1-ps time constant) [42] . The leap-frog integrator was employed with a 1 fs time step. In this work, we considered a system of 80 **E(0)**, 40 **E(0)**$^{+}$, and 40 **E(1)**$^{-}$ molecules (i.e., a 25 mol% CO_2 loading system). The molecular system (simulation box), initially generated at random in a 5.0×5.0×5.0 nm^3 box, was first statically optimized. This was followed by a 100 ns NVT run (298 K). Next, an NPT simulation (1 bar, 298 K) was performed, resulting in a box of 3.89×3.89×3.89 nm^3. Finally, a 1-microsecond production run was performed.

WAXS procedures and data analysis

WAXS monitoring of the clustering process

WAXS methods measurements and data acquisition at STP. The equilibration of EEMPA samples upon CO_2 exposure at each pressure was monitored using WAXS for duration ranging from 1 to 4 hr, the highest initial pressures/stoichiometries requiring the longest equilibration times. Sample scattering data was corrected for sample container background and sample geometry, and normalized by time, transmission coefficient, and the intensity scaled to absolute units of differential scattering cross section per unit volume. Each scattering run was collected for 5 minutes using beam dimensions of 0.6 mm x 1.0 mm (horizontal × vertical) at the sample. The two-dimensional scattering pattern was collected on a Dectris Pilatus 200k detector with 0.172 mm pixel size, and the azimuthally symmetric pattern was averaged into one-dimensional curves for further background subtraction and scaling to absolute units of differential scattering cross section per unit volume. The one-dimensional data files consist of 3 columns: Q, I(Q), and statistical error of I(Q) (see Supporting Data) avec provides the mean values ad error bars displayed in **Fig. 3E** and **Supplementary Fig. 20**.

WAXS data processing methodology and fitting. The WAXS curves were fit (SASview version 5.0.4, http://www.sasview.org/) over the measured range using a Teubner-Strey model[43,44] for the low-Q region of the spectra and three Gaussian peaks for the higher angle high-intensity peak that consists of various short nearest molecular neighbor correlations. The Teubner-Strey (TS) model applied at low Q describes the periodicity and correlation length due to regions of alternating electron density distribution induced by CO_2 absorption. The TS+3G model was fit to the fully reduced data using SASview (version 5.0.4, http://www.sasview.org/). The following default fit algorithm and tolerances were used: Levenberg-Marquardt with 200 steps, f(x) tolerance of 1.5E-8, and x tolerance of 1.5E-8. The resulting goodness of fit is provided as reduced chi squared weighed by the experimental counting statistics (χ^2) and a reported as follows (in order of increasing CO2 loading) as χ^2 = 1.02, 1.11, 1.20, 0.98, 1.00, 1.54, 1.27, 1.33, and 1.68.This provided the mean values and error bars displayed at **Fig. 3F.**

The full two-phase Teubner-Strey model with three Gaussian peaks (TS+3G) is presented below:

$$I(q) = \frac{8\pi\varphi_a(1-\varphi_a)(\Delta\rho)^2 c_2/\xi}{a_2+c_1q^2+c_2q^4} + \sum_{i=1}^{3} A_i e^{-\frac{1}{2}\left(q-q_{0,i}\right)^2/\sigma_i^2} + bgd \quad (15)$$

In this model, φ_a is volume fraction of phase a, ξ is the correlation length, and $\Delta\rho$ is the x-ray scattering length density difference between the two phases. Because the term $(1-\varphi_a)(\Delta\rho)^2$ depends on precise knowledge of the density of electron rich core in comparison to its surroundings, this term was held constant and a single scaling parameter. Instead, *A_scale* represents the growth of the CO_2-dependent core phase, presented in **Supplementary Table 8** below. The parameters a_2, c_1, c_2 are related to the periodicity d and correlation length ξ as follows:

Periodicity d is given by:

$$d = 2\pi \left[\frac{1}{2}\left(\frac{a_2}{c_2}\right)^{1/2} - \frac{1}{4}\frac{c_1}{c_2}\right]^{-1/2} \quad (16)$$

And the correlation length is given by:

$$\xi = \left[\frac{1}{2}\left(\frac{a_2}{c_2}\right)^{1/2} + \frac{1}{4}\frac{c_1}{c_2}\right]^{-1/2} \quad (17)$$

The three Gaussian peaks are used to fit the primary broad peak signal between 1.32 and 1.40 $Å^{-1}$, which is due to a number of nearest neighbor intermolecular correlations. The list of obtained fitting parameters for the TS+3G model are presented in Supplementary **Tables 8-11**.

The amphiphilicity factor, f_a, is useful in describing the phase stability of the system and is related to the Teubner-Strey model parameters as: $f_a = \frac{c_1}{(4a_2c_2)^{1/2}}$. The original definition of the amphiphilicity factor is in relation to the water-water structure function. **Supplementary Table 8** describes the physical properties corresponding to different values of f_a (adapted from reference[47]). The α dependence of the amphiphilicity factor for EEMPA is shown in **Supplementary Fig. 31.**

Supplementary figures

pKa determination

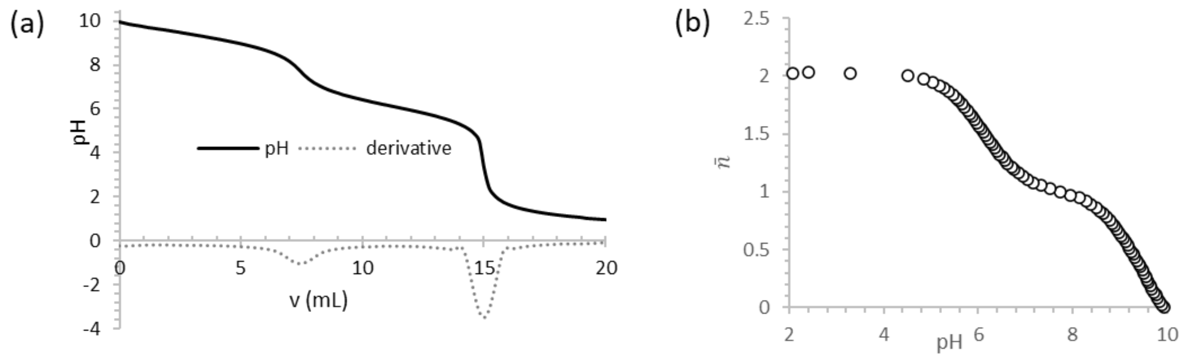

Supplementary Fig. 4.
Titration curve of aqueous EEMPA 0.37 M with aqueous HCl 0,5 M (a) and plot the protonation state of **E(0)** vs pH in aqueous medium (Inflection points correspond to) the pK_a value (b).

Gas-liquid partition

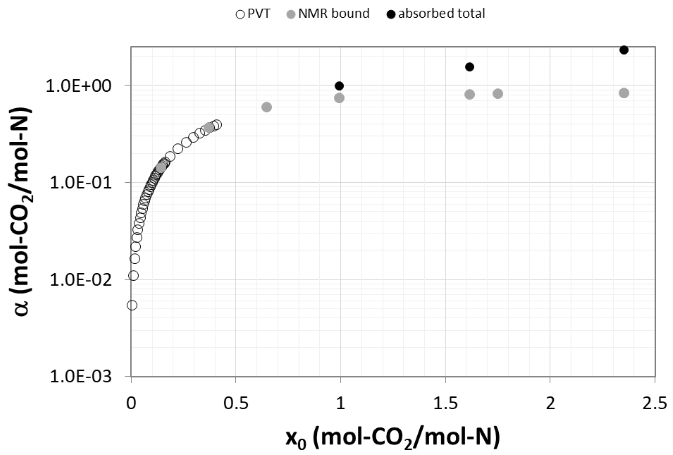

Supplementary Fig. 5.
EEMPA CO_2 binding isotherm. Plot of the chemical loading α vs initial CO_2/EEMPA stoichiometry x_0. Empty circles : PVT measurements. Full dots : q^{13}C NMR data (black : including dissolved CO_2; grey : without dissolved CO_2). Perfect match between PVT (white dots) and q^{13}C NMR measurements (grey dots) cross-validates both quantification methods.

According to IUPAC recommendations, the binding isotherms is defined by the relation, at constant temperature, between α, the fraction of ligand (herein CO_2) bound to a receptor (herein EEMPA) vs the amount of unabsorbed ligand (herein free CO_2 in the headspace) noted x(unbound). In most set-ups (in particular those devoted to adsorption of CO_2 on solid material), the gas is supplied in large excess so that the amount bound can be neglected with respect to the global reservoir. This lead most authors to approximate the amount of unbound gas to the total amount of gas introduced, i.e. the equilibrium partial pressure P^*_f and the initial partial pressure P^*_0. Equilibrium pressure measurements confirmed the linear relationship in our set up between x(unbound) and x_0 (**Supplementary Fig. 6**). This validates our systematic use of x_0 as the x-axis of binding isotherms.

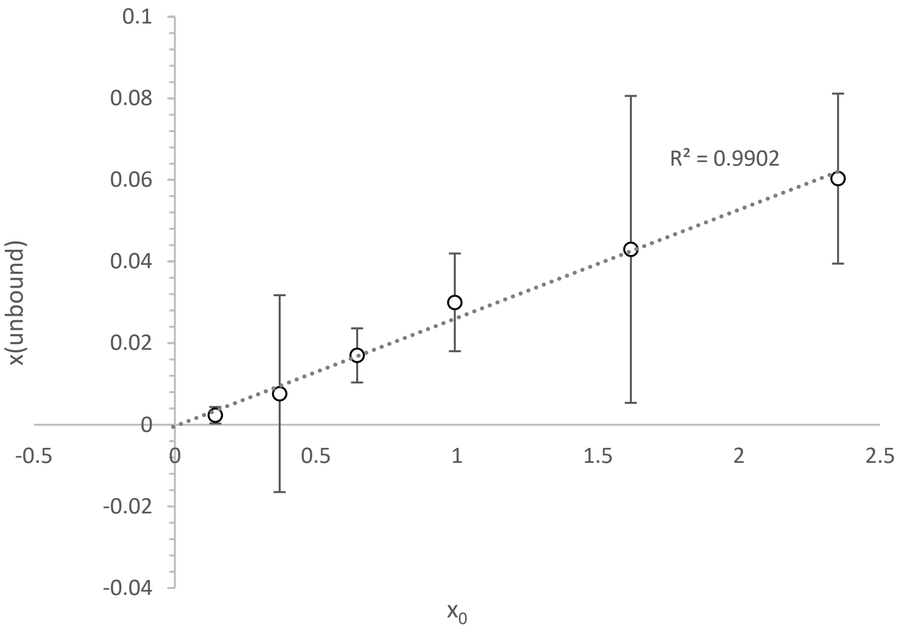

Supplementary Fig. 6.
Gas/liquid CO_2 partition upon EEMPA loading. molar fraction of CO_2 in the headspace x(headspace) derived from pressure measurements vs initial CO_2 to EEMPA molar fraction x_0.

P^*_f can extrapolated from $x_{unbound}$, the molar fraction of CO_2 which was neither dissolved in EEMPA (physically captured) nor reacted with EEMPA (chemically bound). This fraction was calculated by subtracting the total amount of CO_2 initially introduced measured by gravimetry to the molar amount CO_2 bound into the liquid phase measured by q^{13}C NMR. NMR (as the mean value from the integration of at least four different signals from **E(1)** in q^{13}C NMR. Error bars were calculated as the standard deviation from these four integral values). P^*_0 can be measured independently (see PVT measurement section) and was found to correlate with x_0 through the perfect gas law within less than 2% deviation. Therefore, the same law could reasonably be applied to evaluate P_f from n_f.

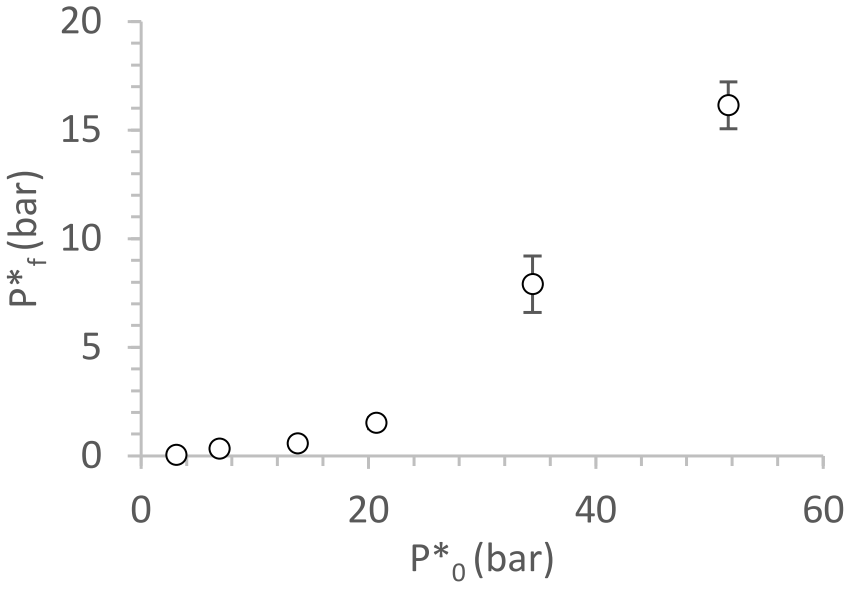

Supplementary Fig. 7. Relationship between initial partial pressure in CO_2 P_0* and equilibrium/final partial P_f* pressure in CO_2 in our setup monitored experimentally. Mean values and error bars originate from the same measurement as for **supplementary Fig. 6.**

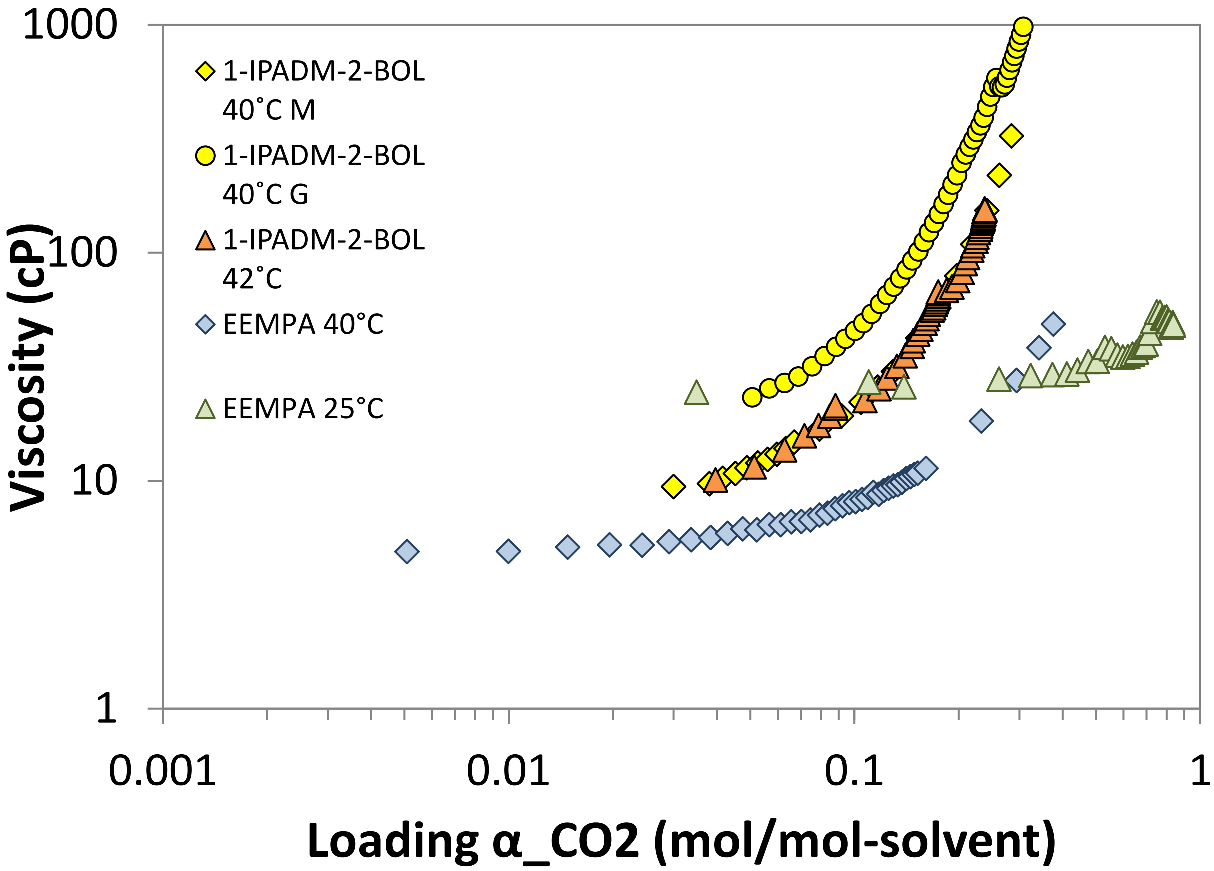

Supplementary Fig. 8. Viscosity change of samples upon CO_2 loading a in the reaction cell for alkanolguanidine water-lean solvents (yellow and orange marks, from ref.[48]) and EEMPA (blue mark) at 40°C and for EEMPA at 25°C (green mark). The viscosity was measured at shear rate of 100 1/s.

Polarity measurements

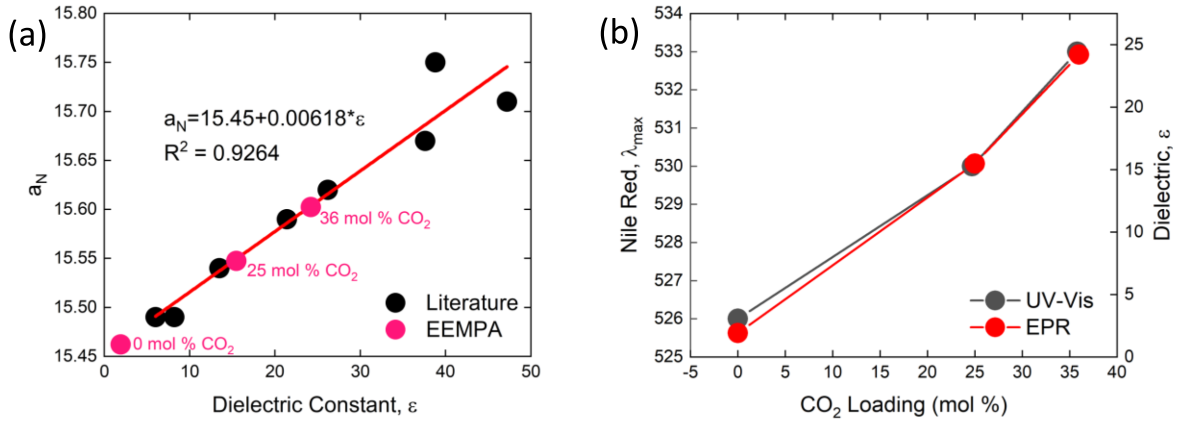

Supplementary Fig. 9. Polarity measurement on neat EEMPA samples upon gradual CO_2 loading. (a) Nitrogen isotropic hyperfine coupling constant a_N as a function of dielectric constant for solutions of Tempol in various polar non-hydroxylic solvents including EEMPA at α = 0, 25 and 36 mol% CO_2 loading. (b) Comparison of the Nile Red dye λ_{max} and the dielectric constant calculated through EPR as a function of CO_2 loading.

Free EEMPA has a polarity like PPG-1200 with an absorption maximum of 526 nm. CO_2 loaded EEMPA exhibited a red shift in the absorbance spectrum. At 25 mol% CO_2 and 36 mol % CO_2 loading, the absorbance maximum are located at 530 and 533 nm, respectively, which indicates an increase in polarity of the medium. Based on the nile red λ_{max} values, the EEMPA solvent polarity shifts from a nile red similar to PPG-1200 to t-butanol (5.0 Mpa CO_2, 25 °C) in the loading range mentioned above.

The nitrogen isotropic hyperfine coupling constant, a_N, was measured for EEMPA, 25 mol % CO_2-loaded EEMPA, and 36 mol % CO_2-loaded EEMPA. As the CO_2 loading increased, a_N increased from an initial value of 15.46 to 15.54 and 15.60 for 25 mol% and 36 mol% CO_2 respectively. The increase of a_N with CO_2 loading was expected from Nile Red experiments as a_N is known to increase with increasing solvent polarity due to the stabilization of the nitroxide radical. a_N was found to linearly correlate with the various solvent polarity parameters: Wertheim parameter ($\mu\rho$ ln ε/MW), dielectric constant (ε) and the Dimroth-Reichardt parameter (E_T-30) [47]. The dielectric constant of EEMPA, 25 mol % CO_2-loaded EEMPA and 36 mol % CO_2-loaded EEMPA was extrapolated from the linear relationship between the dielectric constant of polar non-hydroxylic solvents that include ethyl acetate, acetonitrile and dimethyl sulfoxide (DMSO) and a_N shown in **Supplementary Fig. 9a**. The linear regression presented in literature gave a better fitting with r = 0.963 with an intercept of 15.45 and a slope of 0.0062.[47] The dielectric constant of EEMPA increases from 1.92 to 15.47 and 24.2 with 25 mol% and 36 mol% CO_2 loading, respectively, due to the formation of charged species.

Comparison of the dielectric constant calculated through the EPR technique to the solvatochromic technique using Nile red dye show the same trend with increasing CO_2 loading, shown in **Supplementary Fig. 9b**. The trend confirms the increasing polarity of EEMPA as CO_2 loading increased.

Binding isotherms

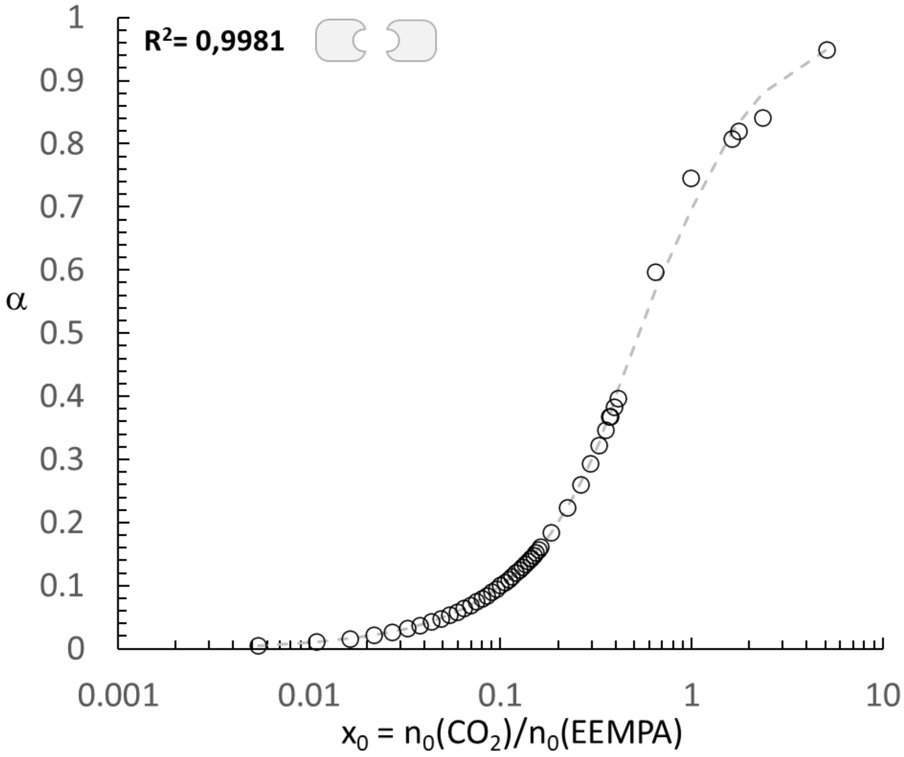

Supplementary Fig. 10. Fitting of the experimental chemical loading α values (circles) with increasing CO_2/EEMPA stoichiometries x_0 (logarithmic scale)with **the dimeric model** and resulting RMSD value.

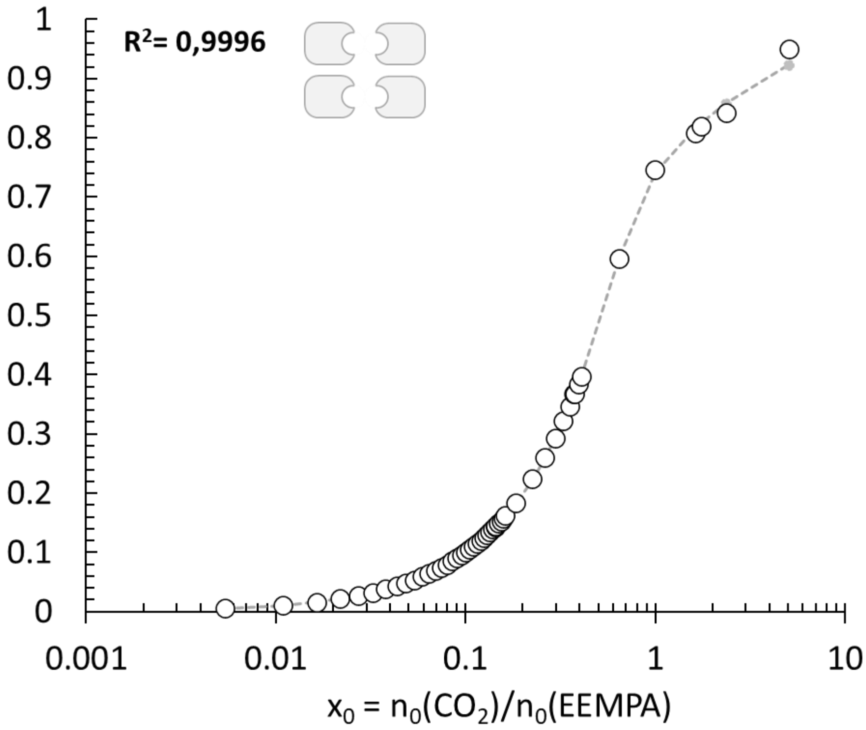

Supplementary Fig. 11. Fitting of the experimental chemical loading α values (circles) with increasing CO_2/EEMPA stoichiometries x_0 (logarithmic scale) with **the tetrameric model** and resulting RMSD value.

NMR speciation

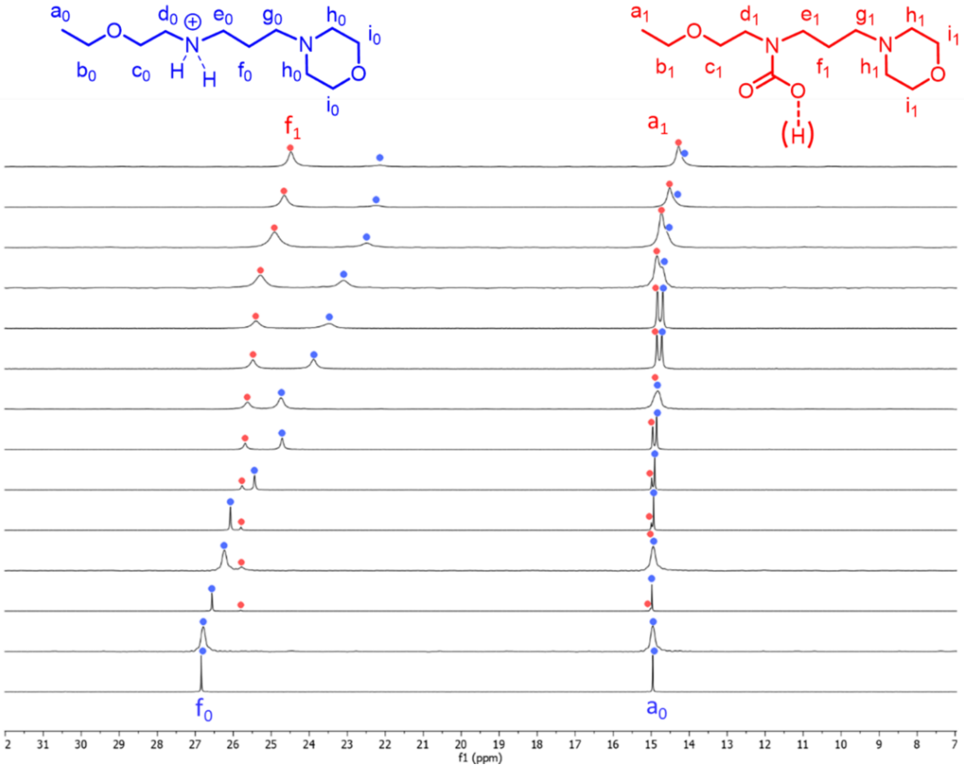

Supplementary Fig. 12. q^{13}C NMR stacked spectra of EEMPA upon gradual loading with CO_2 (bottom to top, see **Supplementary Table 6** for values) focused on nuclei C_a and C_f. Blue : **E(0)**$^{(+)}$, Red : **E(1)**$^{(-)}$

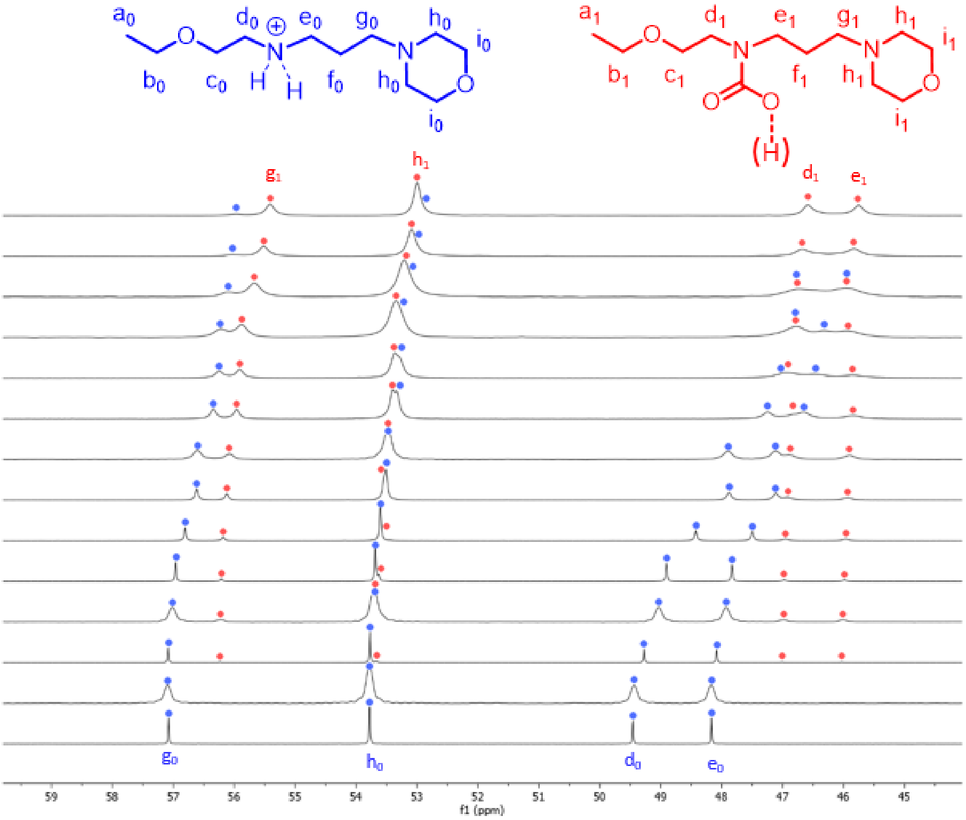

Supplementary Fig. 13. q^{13}C NMR stacked spectra of EEMPA upon gradual loading with CO_2 (bottom to top, see **Supplementary Table 6** for values) focused on nuclei C_d, C_d, C_g and C_h. Blue : **E(0)**$^{(+)}$, Red : **E(1)**$^{(-)}$

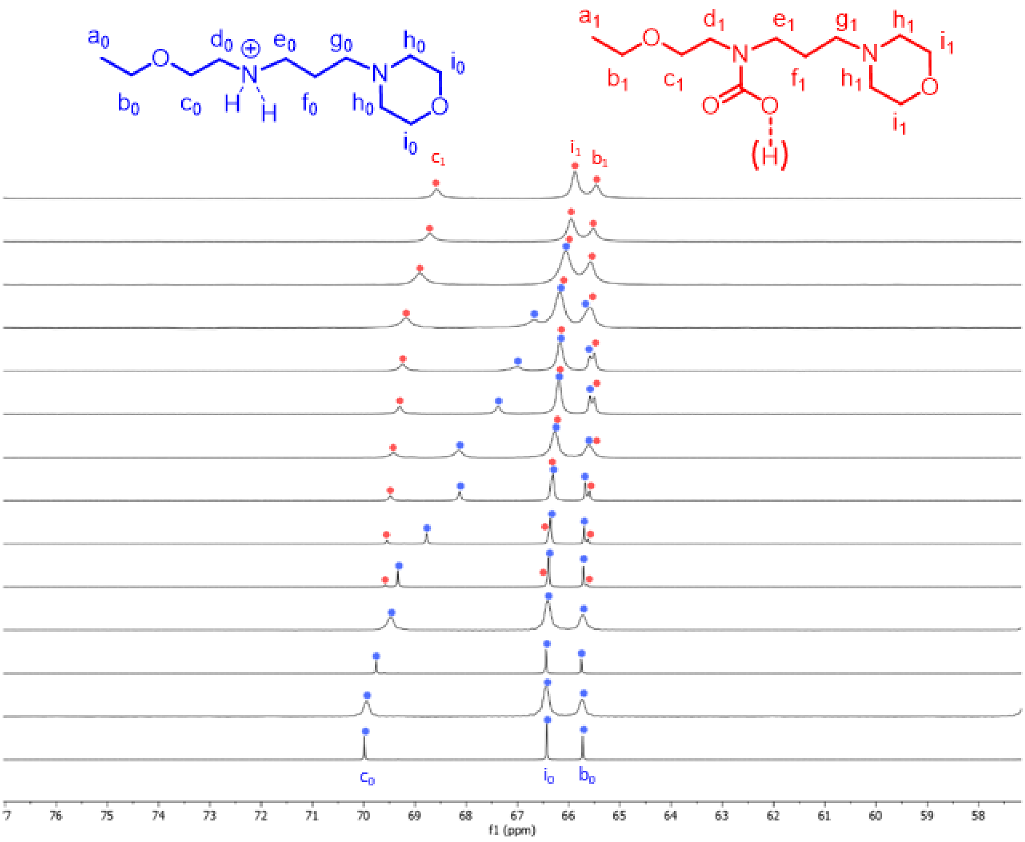

Supplementary Fig. 14. q^{13}C NMR stacked spectra of EEMPA upon gradual loading with CO_2 (bottom to top, see **Supplementary Table 6** for values) focused on nuclei C_b, C_c and C_i. Blue : **E(0)**$^{(+)}$, Red : **E(1)**$^{(-)}$

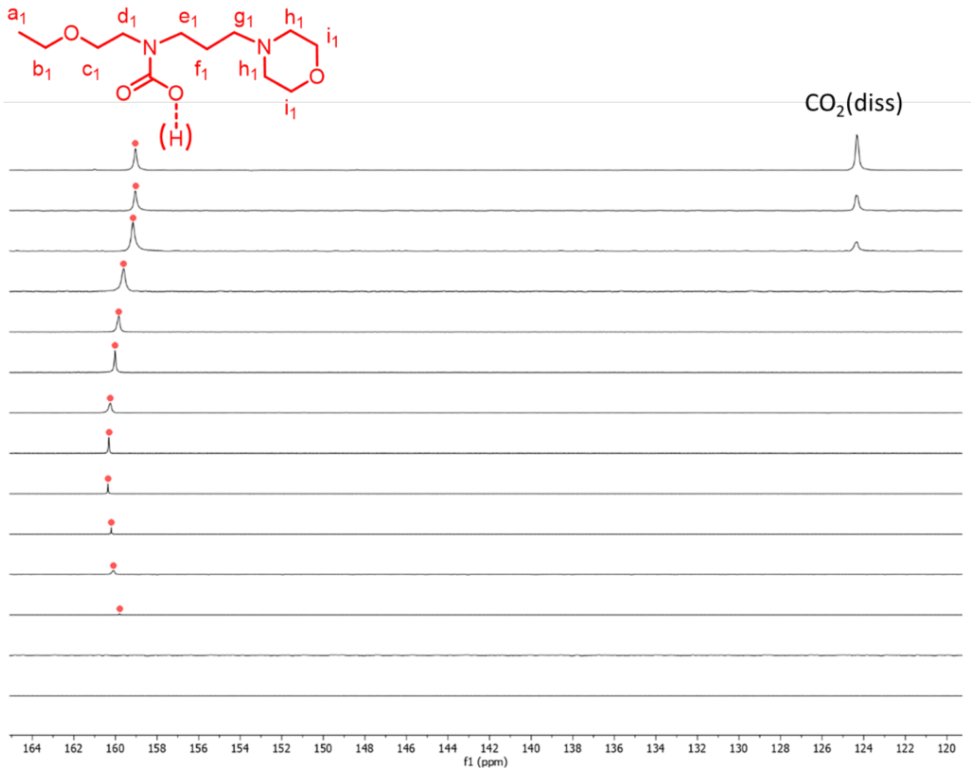

Supplementary Fig. 15. q^{13}C NMR stacked spectra of EEMPA upon gradual loading with CO_2 (bottom to top, see **Supplementary Table 6** for values) focused on carbonyl and dissolved CO_2 signals. Blue : **E(0)**$^{(+)}$, Red : **E(1)**$^{(-)}$

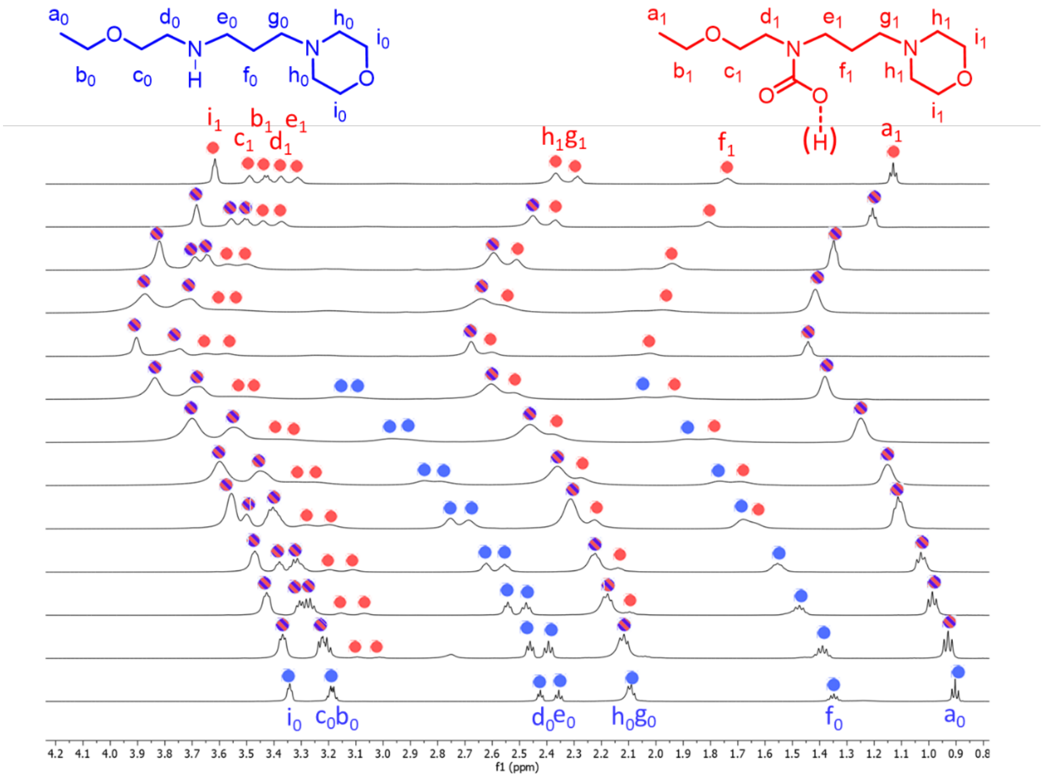

Supplementary Fig. 16. q^{1}H NMR stacked spectra of EEMPA upon gradual loading with CO_2 (bottom to top, see **Supplementary Table 6** for values) focused on CH signals (0.8 – 4.2 ppm).

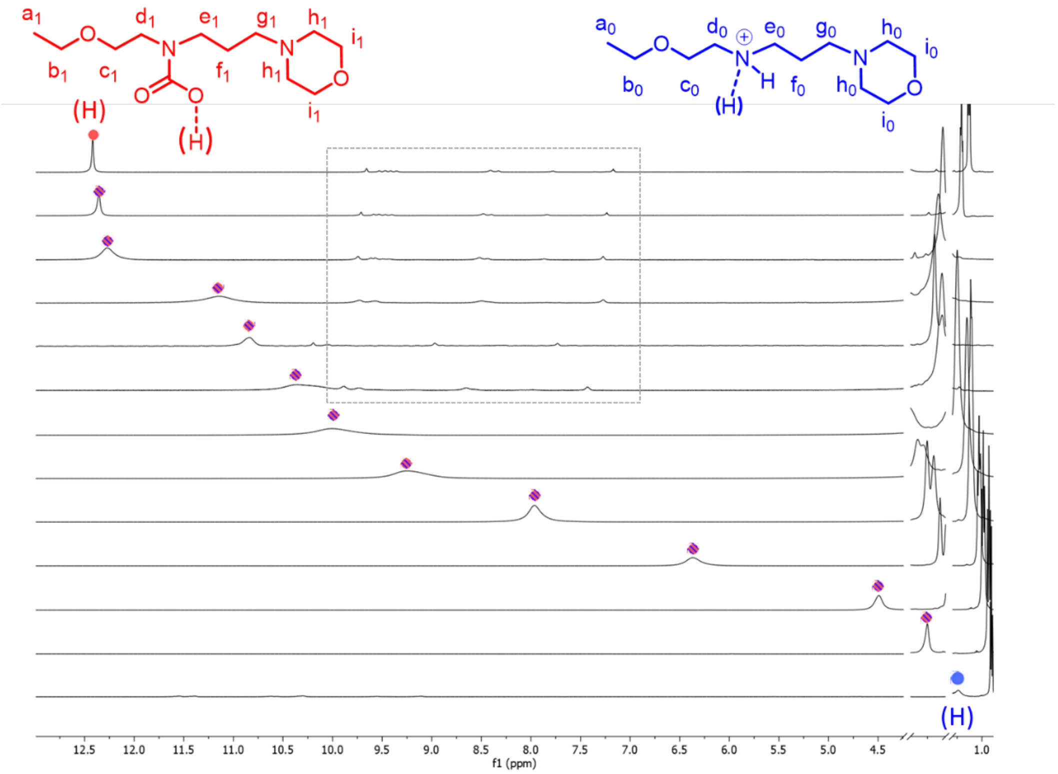

Supplementary Fig. 17. q^{1}H NMR stacked spectra of EEMPA upon gradual loading with CO_2 (bottom to top, see **Supplementary Table 6** for values) focused on XH signals (X = N, O; 2.5 – 13 ppm).

^{13}C-NMR spectra of the carbamic acid **E(1)**

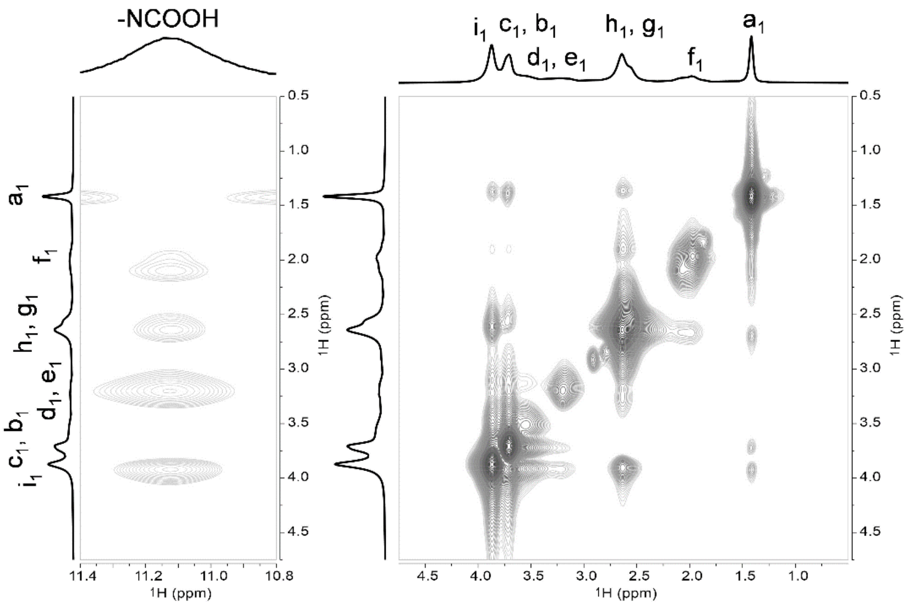

Supplementary Fig. 18. ^{1}H-^{1}H NOESY NMR spectra of EEMPA and CO_2 (α =0.95) at a mixing time of 10 ms.

This experiment reveals that the carbamic acid proton is closer to a morpholine ring (the correlation between NCOOH and f, e, g, h, i, j, k, as well as the lack of correlation between NCOOH and a, b, c, d). These correlations between a and j, k are consistent with the tetrameric clustering into reverse micelles-like architectures.

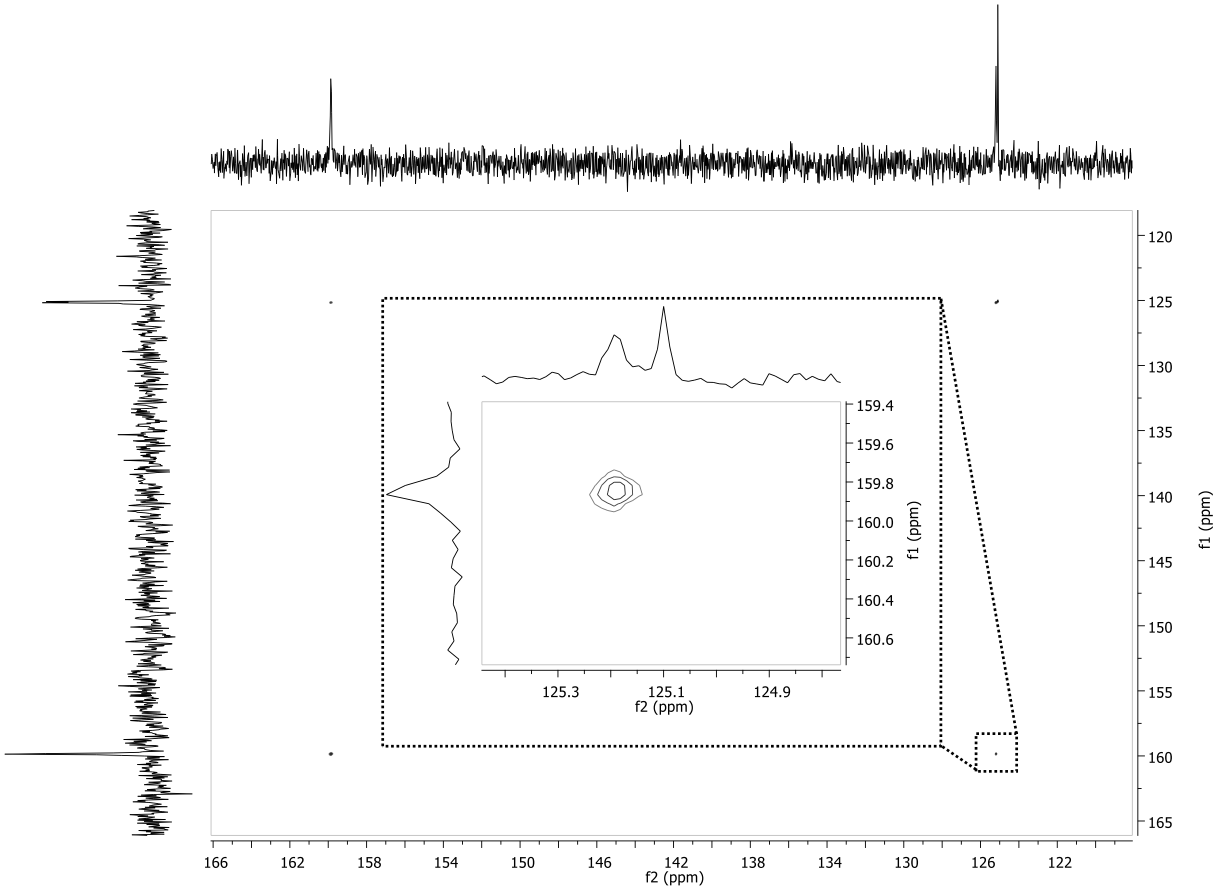

Supplementary Fig. 19. ^{13}C-^{13}C 2D EXSY spectra of CO_2-EEMPA ($\alpha = 0.95$) at a mixing time of 50 ms. cross-peak correlation indicate an equilibrium process between carbamic acid **E(1)** (159 ppm) and dissolved CO_2 (125.2 ppm) but not between carbamic acid **E(1)** and CO_2 gas (124.8 ppm).

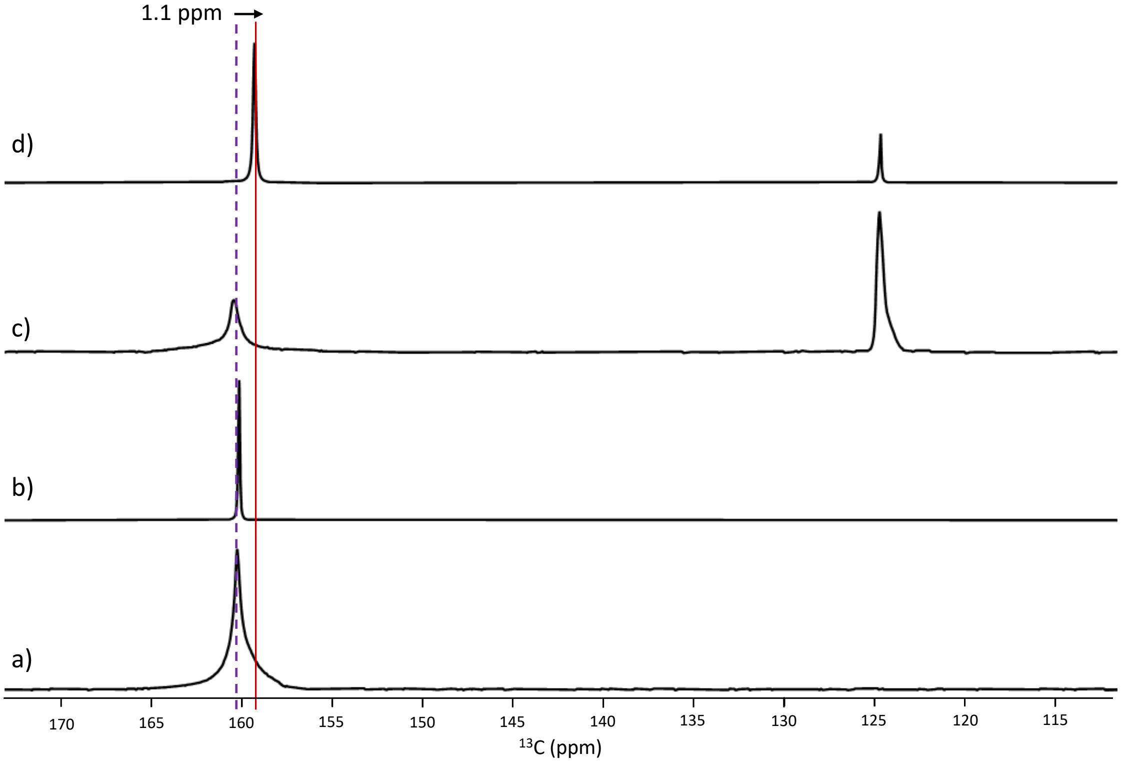

Supplementary Fig. 20. Effect of spinning on the carbamate $E(1)^-$ + dissolved CO_2 to carbamic acid E(1) conversion. ^{13}C of EEMPA and ^{13}C-enriched CO_2, at P^*_0 = 1 bar ($x_0 = \alpha$ = 0.47) in static (a) and MAS conditions (b) and at P^*_0 = 20 bars (x_0 = 0.99, α = 0.75) in static (c) and MAS conditions (d).

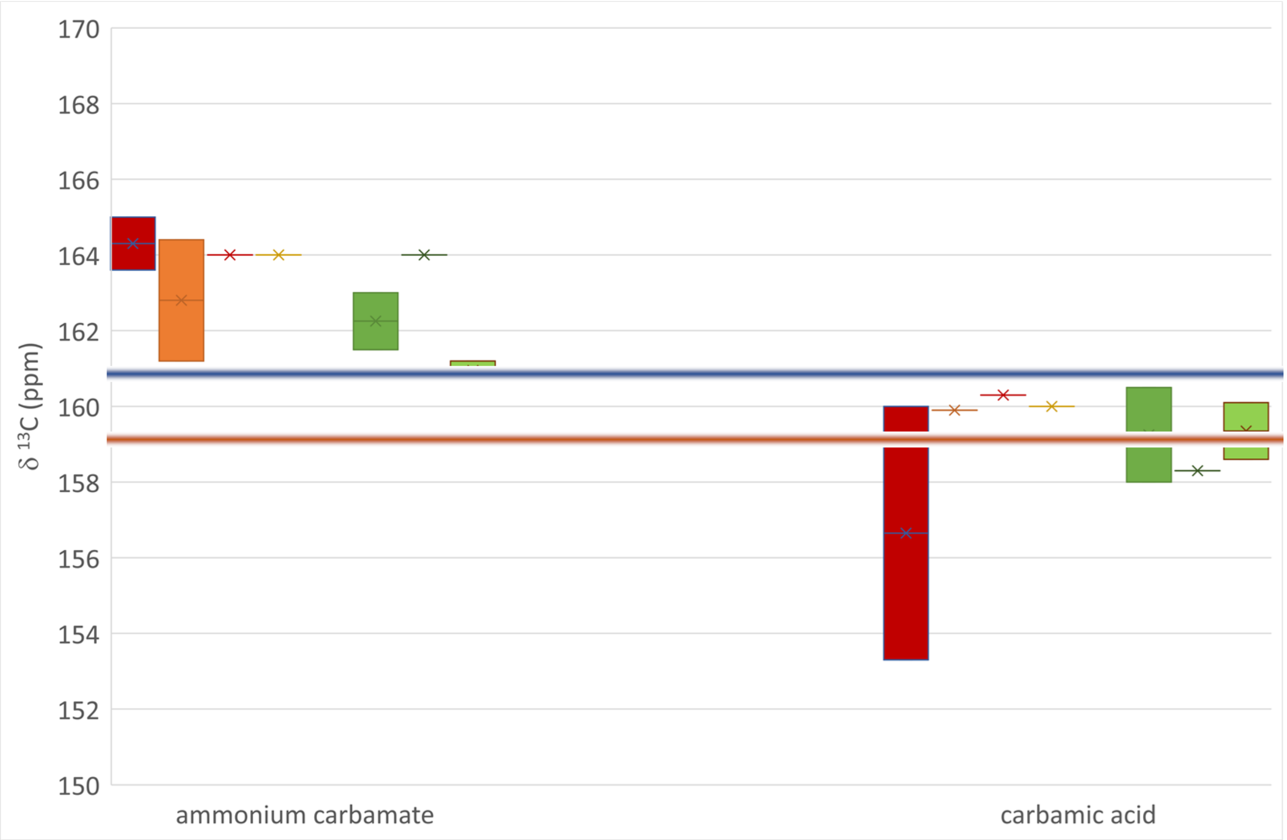

Supplementary Fig. 21. Literature survey (histogram) of ^{13}C NMR shift of CO signals of amine-CO_2 adducts in porous solids[48] (red-orange) and organic solutions[49,50] (green) by category of adduct : ammonium carbamate (left) and carbamic acid (right) and comparison with measured values in the present study (horizontal lines : values obtained in this work ; blue : ammonium carbamate; red : carbamic acid; size of the bar reflect the dispersity of the reported values).

Fitted NMR data

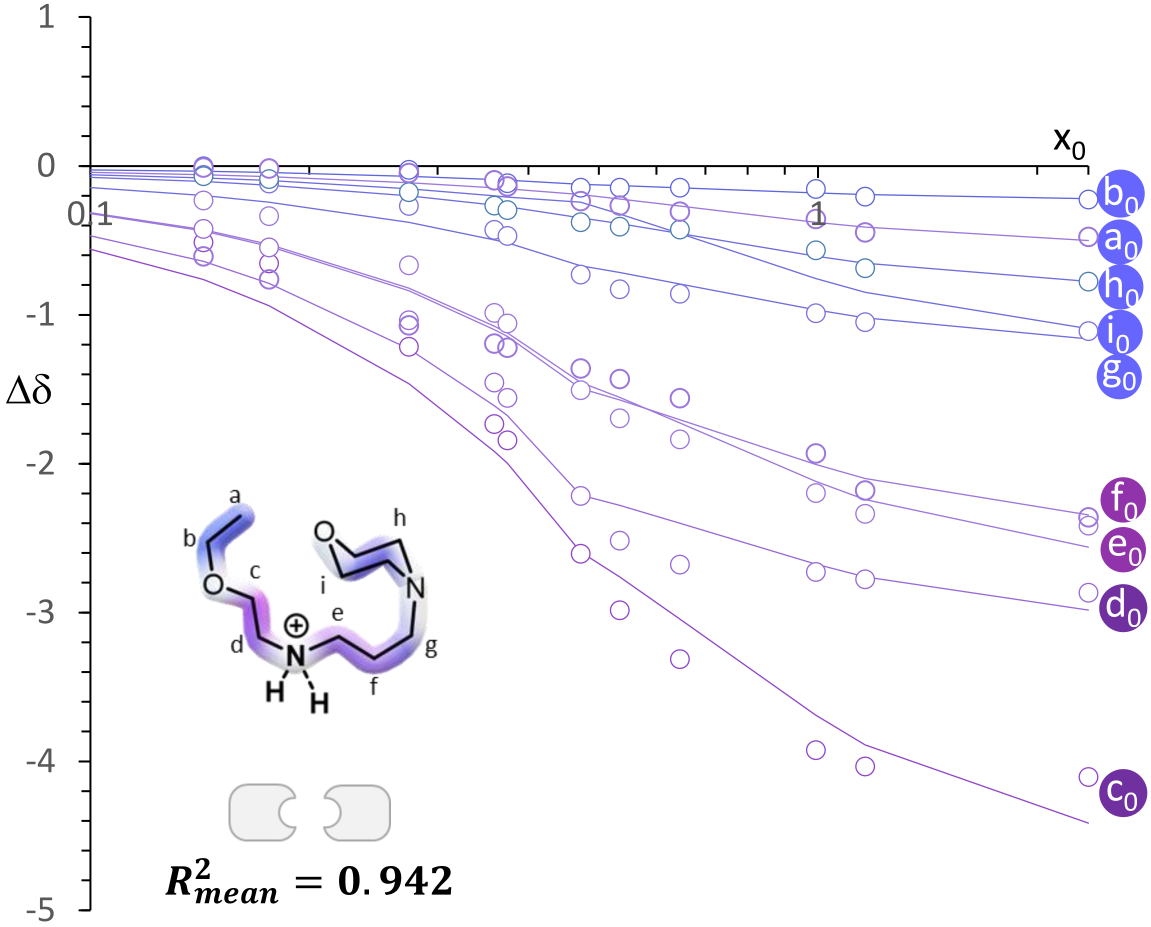

Supplementary Fig. 22. Plot of the Chemical shift perturbation (CSP) Δδ(i) of the ^{13}C nuclei of unloaded **E(0)$^{(+)}$** upon loading with increasing CO_2/EEMPA stoichiometries x_0. Color code reflects the amplitude of perturbation. Dots : experimental data. Line : thermodynamic **dimer model** from Matlab fitting. R^2mean = 0.942 is calculated as the average standard deviation of the full fit series displayed in **Supplementary Figs.22+23**.

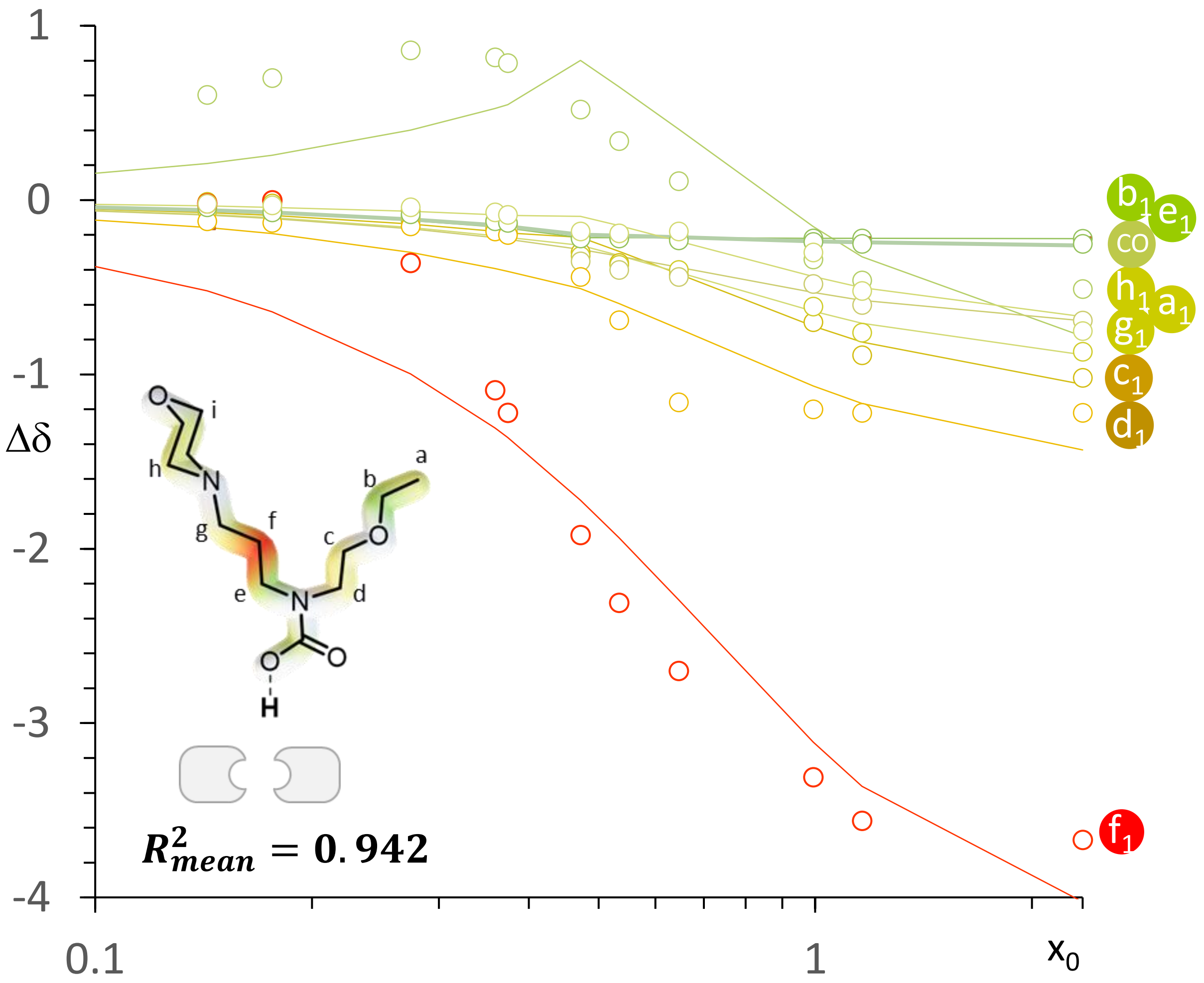

Supplementary Fig. 23. Plot of the Chemical shift perturbation (CSP) Δδ(i) of the ^{13}C nuclei of loaded EEMPA **E(1)**$^{(-)}$ upon loading with increasing CO_2/EEMPA stoichiometries x_0. Color code reflects the amplitude of perturbation. Dots : experimental data. Line : thermodynamic **dimer model** from Matlab fitting. R^2mean = 0.942 is calculated as the average standard deviation of the full fit series displayed in **Supplementary Figs. 22+23**.

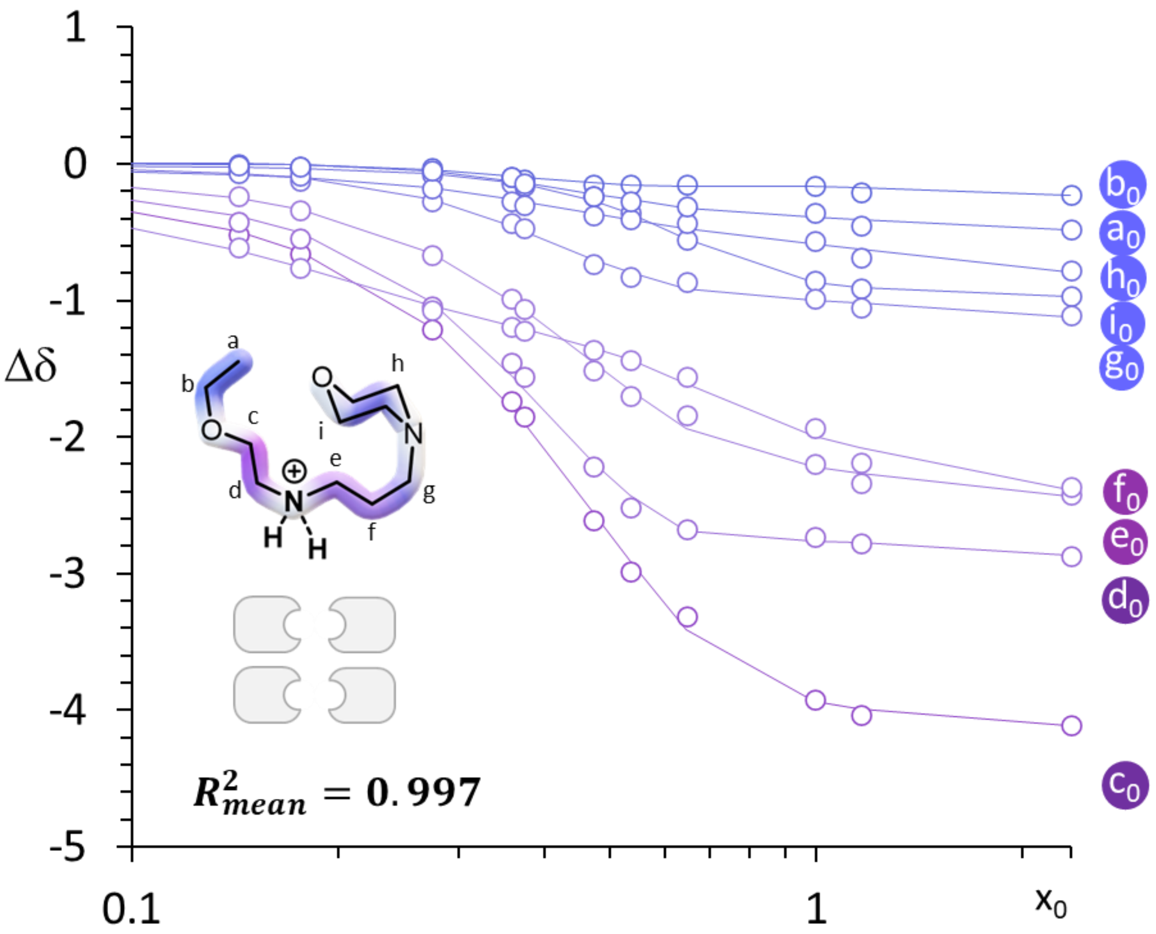

Supplementary Fig. 24. Plot of the Chemical shift perturbation (CSP) Δδ(i) of the ^{13}C nuclei of unloaded **E(0)**$^{(+)}$ upon loading with increasing CO_2/EEMPA stoichiometries x_0. Color code reflects the amplitude of perturbation. Dots : experimental data. Line : thermodynamic **tetramer model** from Matlab fitting. R^2mean = 0.997 is calculated as the average standard deviation of the full fit series displayed in **Supplementary Figs. 24+25**.

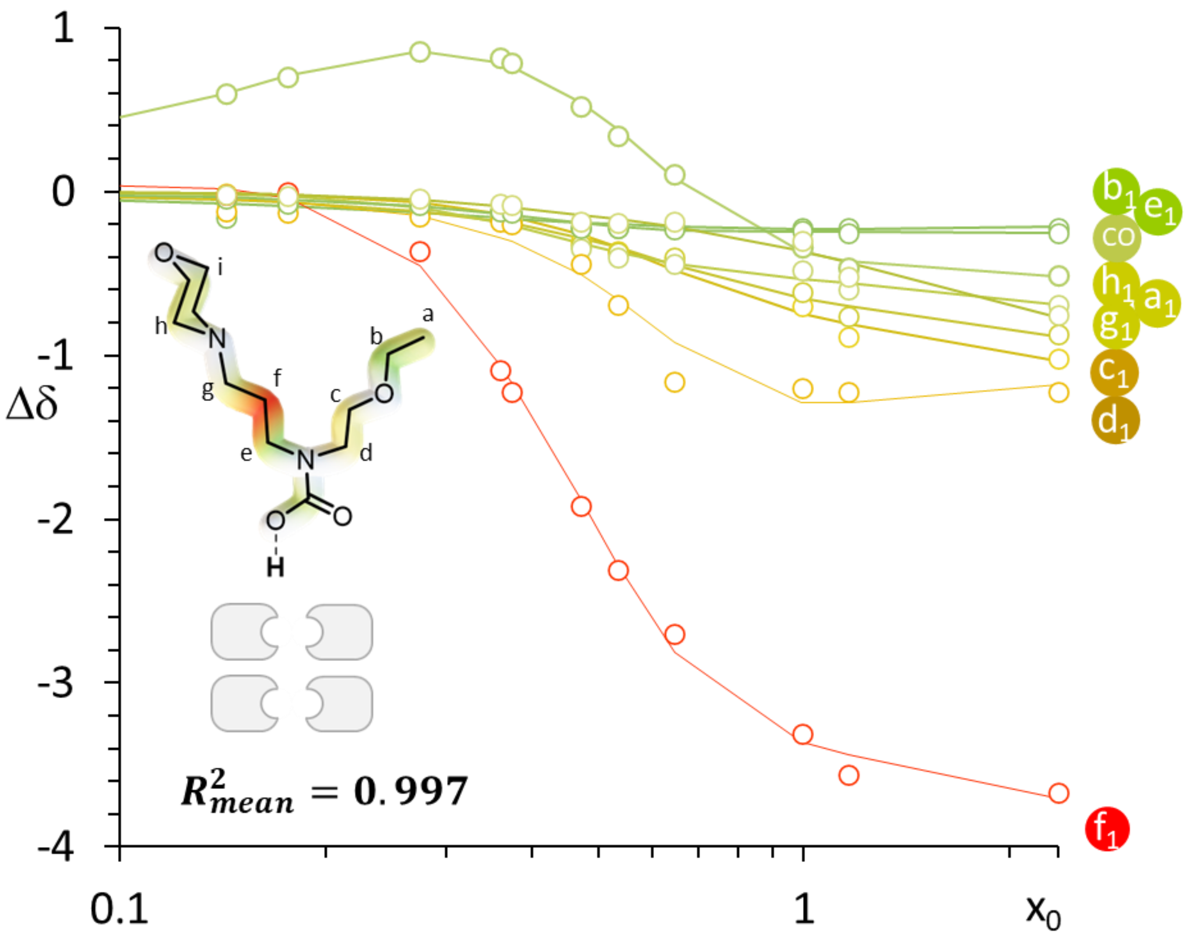

Supplementary Fig. 25. Plot of the Chemical shift perturbation (CSP) Δδ(i) of the ^{13}C nuclei of loaded EEMPA **E(1)**$^{(-)}$ upon loading with increasing CO_2/EEMPA stoichiometries x_0. Color code reflects the amplitude of perturbation. Dots : experimental data. Line : thermodynamic **tetrameric model** from Matlab fitting. R^2mean = 0.997 is calculated as the average standard deviation of the full fit series displayed in **Supplementary Figs.24+25**.

Speciation models

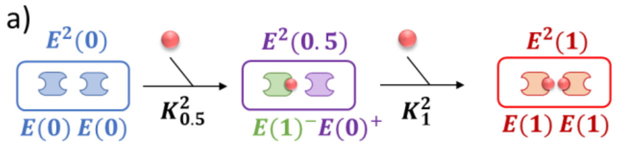

b)

K	value	c
$K^2_{0.5}$	8.9 10^4	-
K^2_1	4.08	4.5 10^{-5}

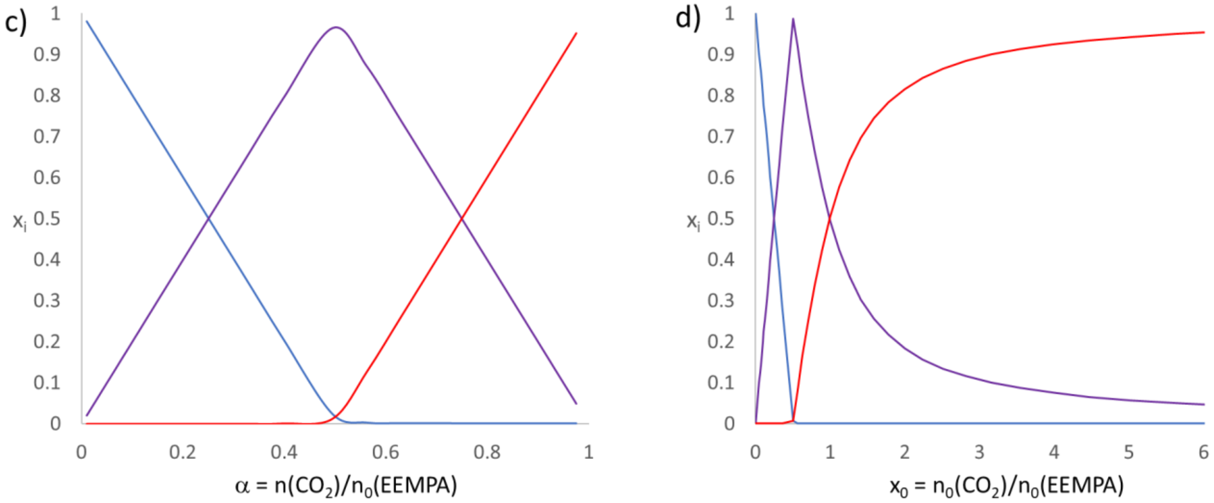

Supplementary Fig. 26. Dimeric model used to fit the NMR data displayed at **Supplementary Figs. 22** and **23** (a) and equilibrium constant obtained (b) (c indicates the cooperativity factor, see ref 51 and speciation resulting from the model plotted as a function of the chemical loading α (c) or initial stoichiometry x_0 (d). This model predicts that the carbamic acid **E(1)** is only present from α = 0.5.

a)

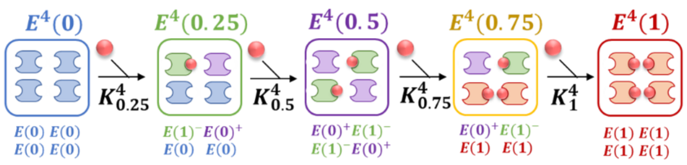

b)

K	value	C
$K^4_{0.25}$	1680	-
$K^4_{0.5}$	107	0.064
$K^4_{0.75}$	32.2	**0.30**
K^4_{1}	0.569	0.018

c) d)

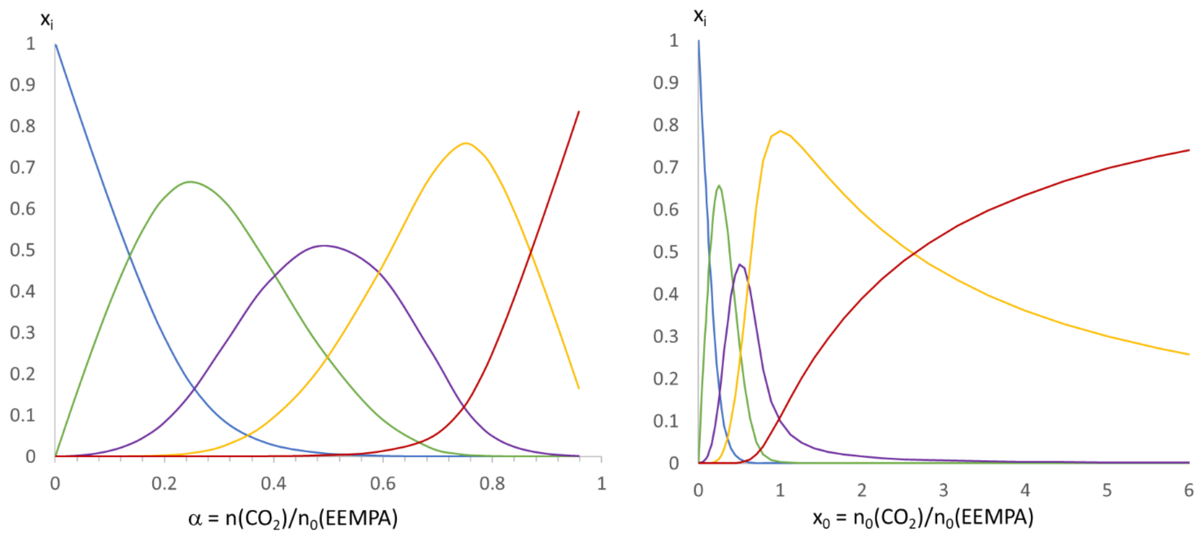

Supplementary Fig. 27. Tetrameric model used to fit the NMR data displayed at **Supplementary Figs. 24** and **25** (a) and equilibrium constant obtained (b) (c indicates the cooperativity factor, see ref 51) and speciation resulting from the model plotted as a function of the chemical loading α or initial stoichiometry x_0. This model predicts that the carbamic acid **E(1)** is present from α = 0.2.

FT-IR data

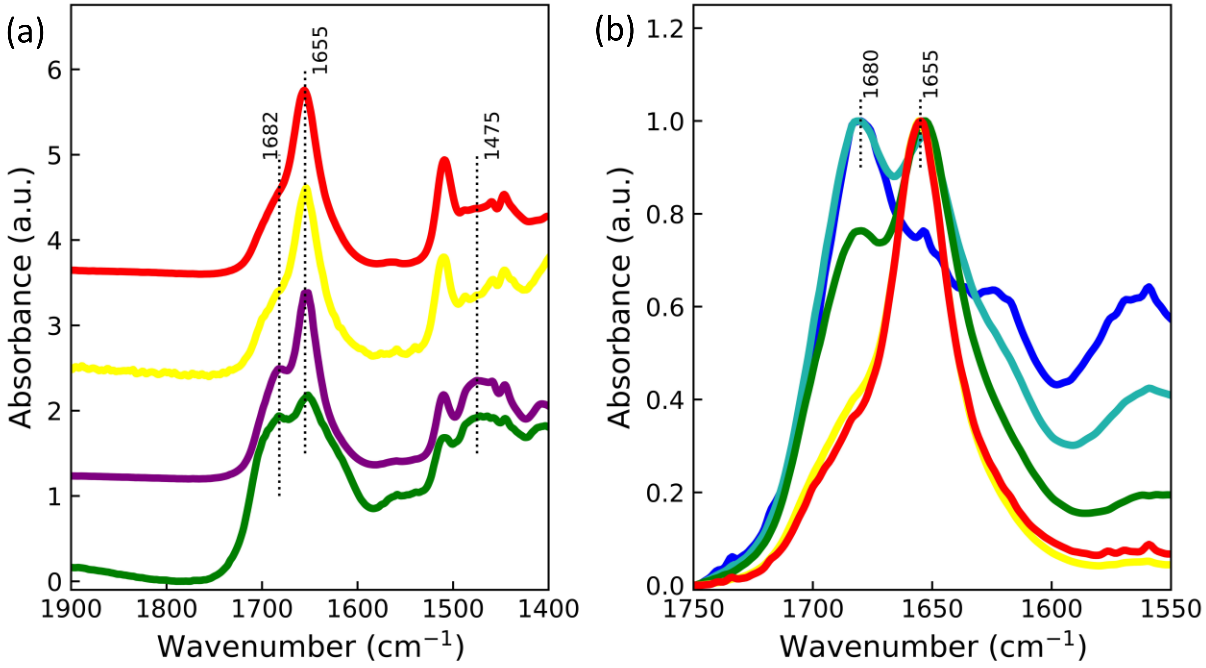

Supplementary Fig. 28. FT-IR spectra of EEMPA during pressurized loading CO_2 and depressurization. Absorbance spectra of EEMPA during increasing CO_2 loading (green : 0.27, purple : 0.58, yellow: 0.89 and red : 0.92; colour code correspond to speciation displayed at **Supplementary Fig. 27**). (b) Absorbance spectra of EEMPA during depressurization from a CO_2 loading of 0.92 (red) to 0.27 upon progressive depressurization

The pattern of signals obtained in FT-IR with EEMPA at low CO_2 loading is constituted of two overlaying peaks in the carbonyl region (around 1655 cm^{-1} and 1682, for α = 027, wherein the singly occupied (green) tetramer **E^4(0.25)** is the main species). This band becomes narrower as the CO_2 pressure in the Parr reactor and the loading increased (up to α = 0.92, from green to red, **Supplementary Fig. 28a**). Depressurization of the Parr reactor (**Supplementary Fig. 28b**) to release CO_2 causes the band at 1682 cm^{-1} to reform as the band at 1655 cm^{-1} to decrease until it fully disappears. Interpretation of this phenomenon is proposed at **Supplementary Fig. 29.**

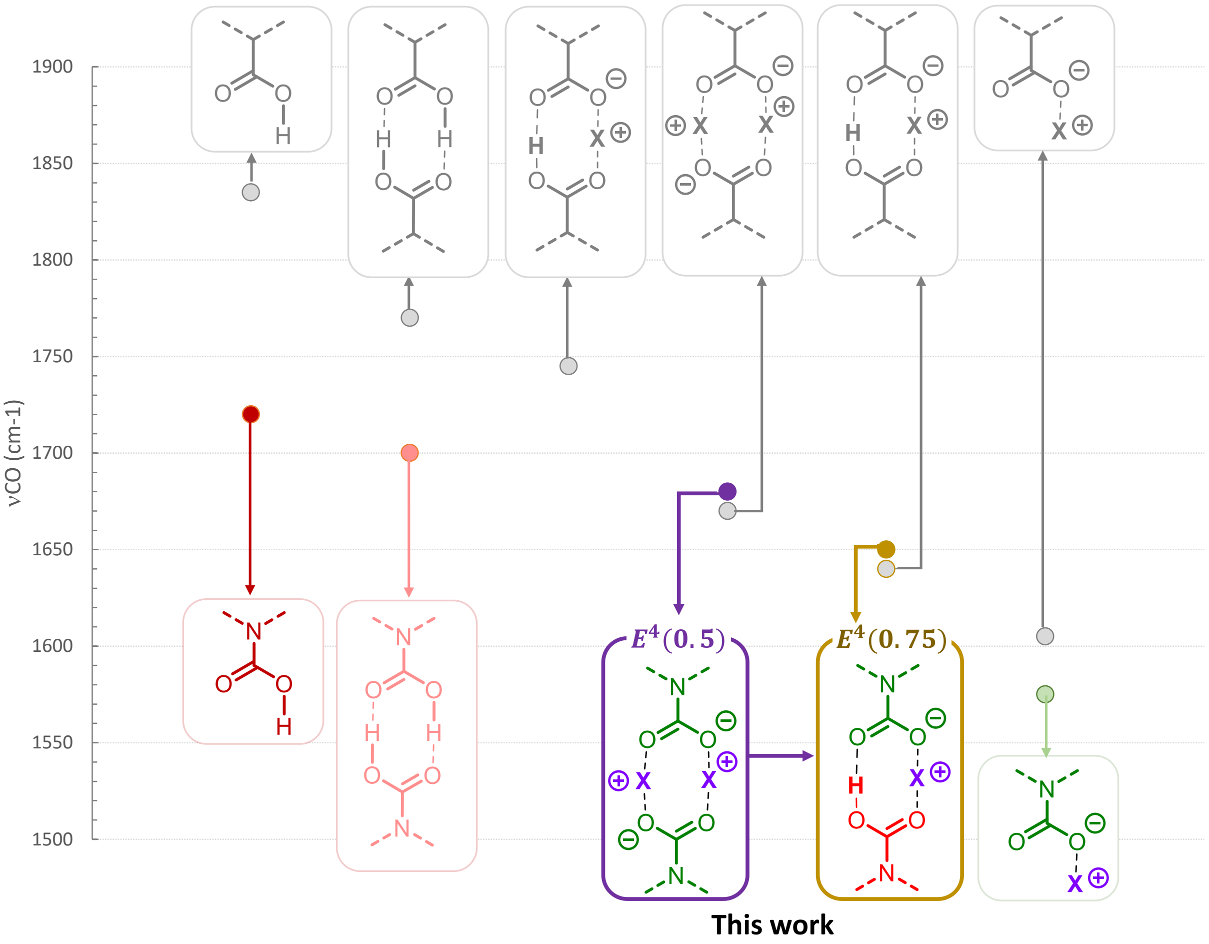

Supplementary Fig. 29. Reported carbonyl stretching frequency in FT-IR spectroscopy for carboxylic species (grey) [54] and carbamates as isolated [53,54] or herein paired species? X+ is the counter-ion which accompanies the carbamate/carbamate. Unusual decrease of the CO stretching frequency for EEMPA between α = 0.3 – 0.9 is attributed to **E[4](0.5)** + CO_2 -> **E[4](0.75)** which corresponds to the conversion of two (ammonium) carbamates into (ammonium) carbamate – carbamic acid pair. Symmetrical and unsymmetrical modes have radically different intensities and only the first one is detected

WAXS data

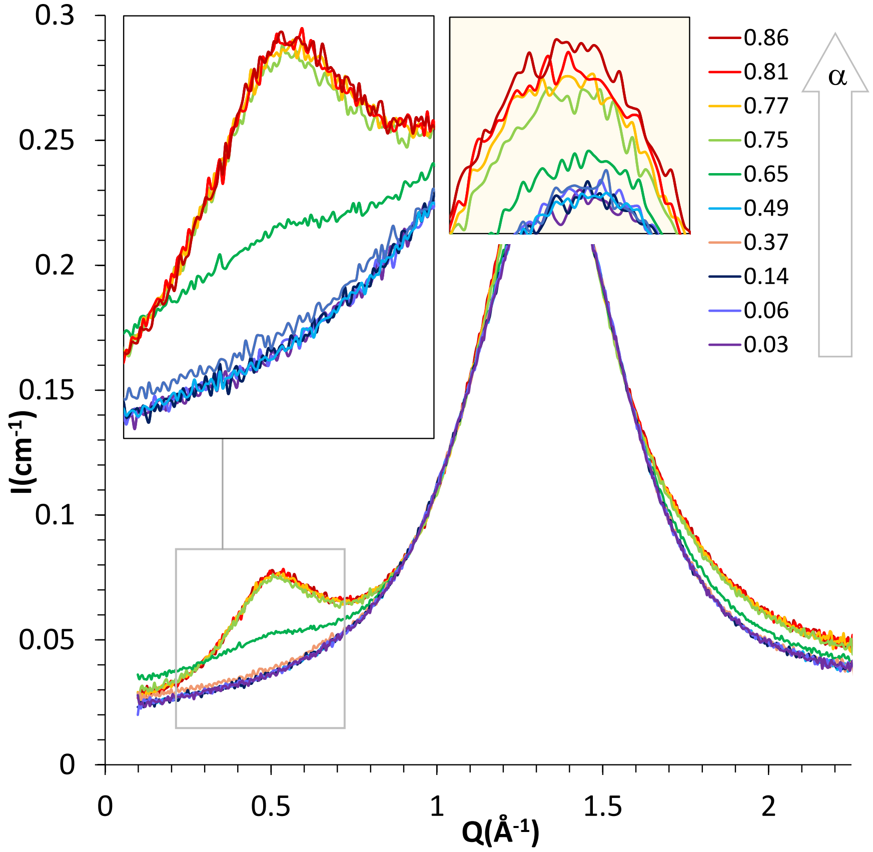

Supplementary Fig. 30. WAXS structural data comparison of EEMPA as a function of pressure-induced CO_2 loading (α). Medium range ordering (MRO) with periodicity of ~12 Å begins to appear at low Q for α from 0.49 – 0.65. The MRO becomes better defined at higher pressures for α from 0.75 – 0.86. The I(Q=0) value, which is proportional to isothermal compressibility, initially increases as unbound CO_2 expands cavities within the solvent, yet drops once the new high-pressure tetramer binds locally available CO_2.

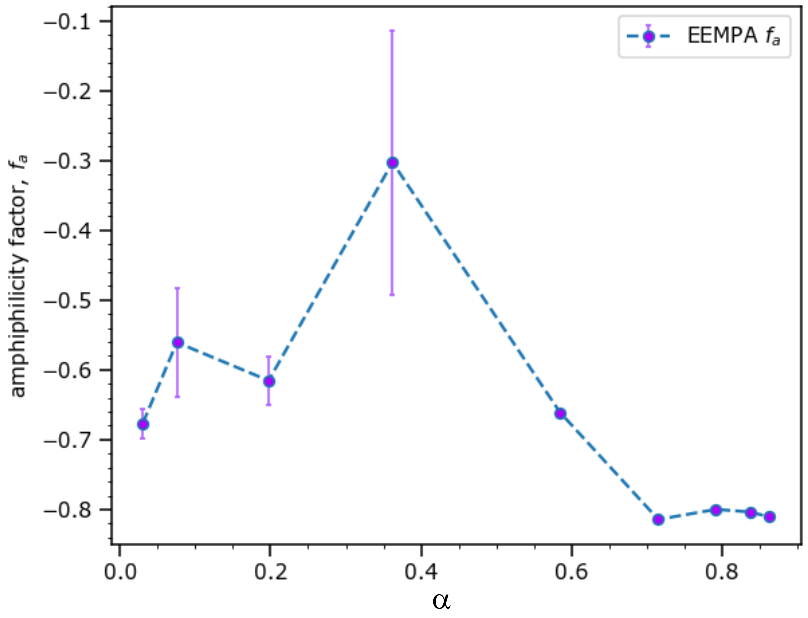

Supplementary Fig. 31. Amphiphilicity factor dependence on CO_2 concentration in EEMPA. During initial CO_2 incorporation, the interface becomes less defined up to α = 0.36, when the structure contains similar quantities of the different CO_2-bound species and as well as gas phase CO_2. Increased pressure drives the system to the primary CO_2-loaded tetramer state and the interfaces demarcating the regions of unique binding chemistry are better defined with f_a ~ -0.8. For error bar and mean value calculation see the method section (**WAXS procedure and data analysis**) of this document.

DFT-computed clustering and absorption energies

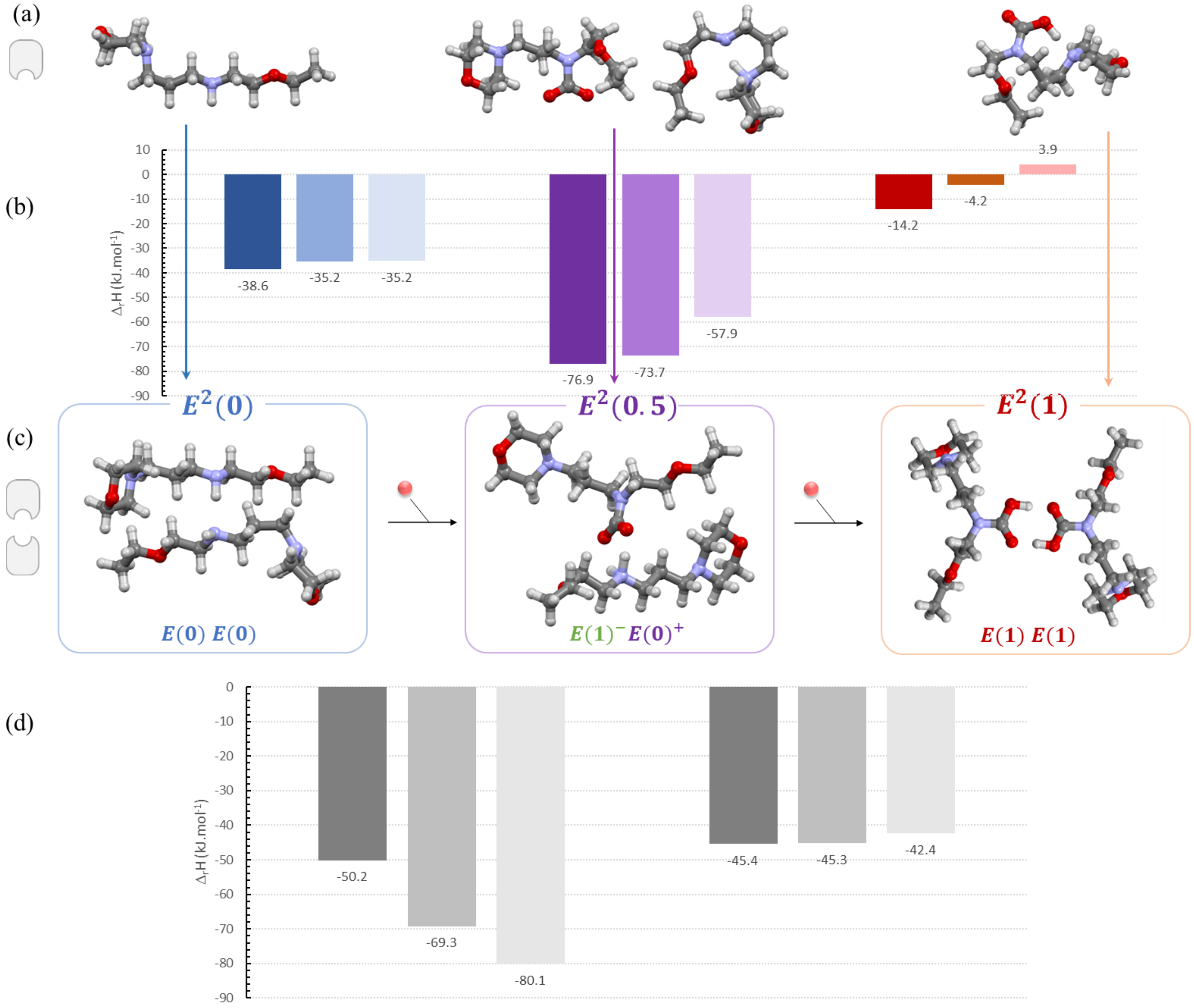

Supplementary Fig. 32. DFT modelling of EEMPA-CO_2 adducts and absorption enthalpies: (a) optimized structures of monomeric constituents (in DMF) and their dimerization enthalpy (b) into corresponding optimized structures (c, in DMF) and successive enthalpies of CO_2 absorption (d) (Atom color code : C (black), H (white), O (red), N (blue); histogram implicit solvent code : DMF (light), t-butanol (medium), propanol (dark). Although all structures were minimized in the three solvents, the choice was made to display minimized structures in the most dissociative one (DMF).

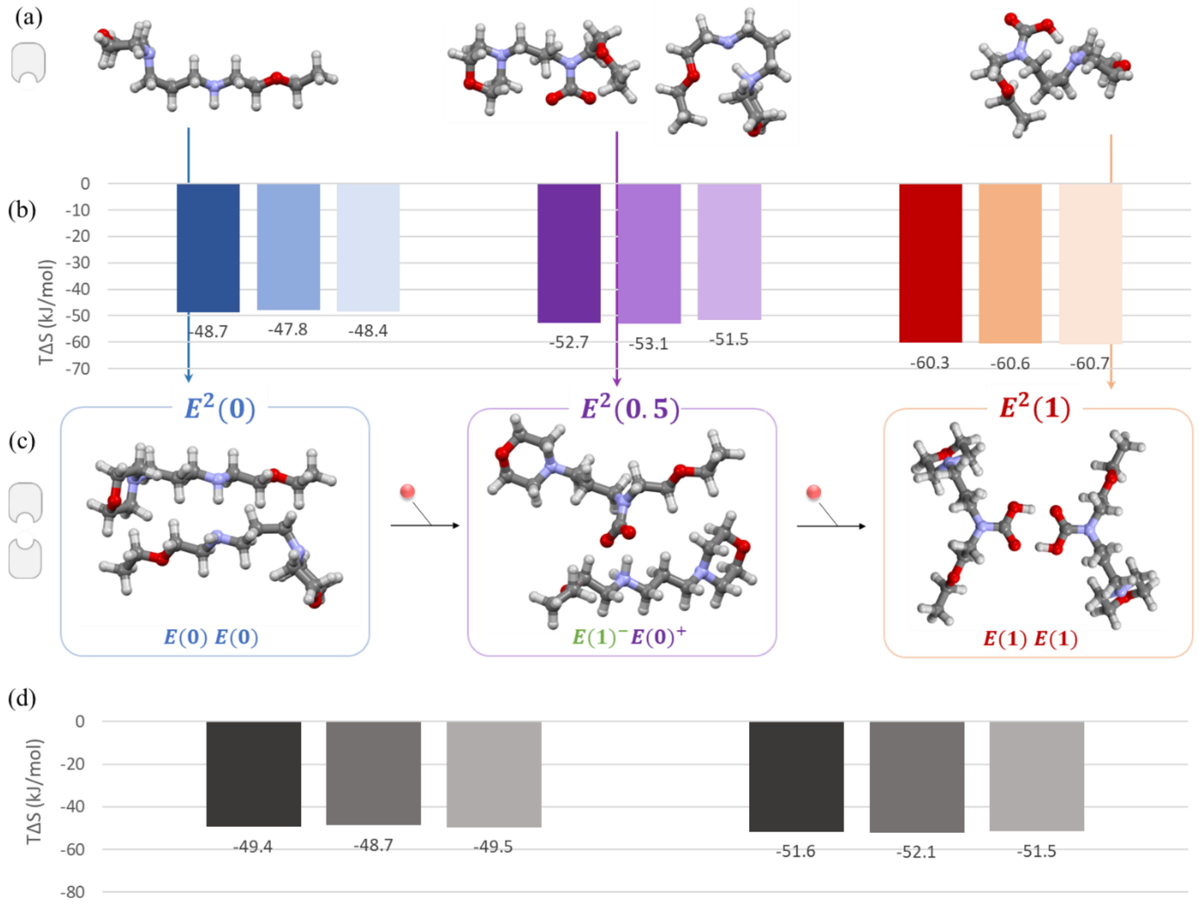

Supplementary Fig. 33. DFT modelling of EEMPA-CO_2 adducts and absorption entropies: (a) optimized structures of monomeric constituents (in DMF) and their dimerization entropy (b) into corresponding optimized structures (c, in DMF) and successive enthalpies of CO_2 absorption (d) (Atom color code : C (black), H (white), O (red), N (blue); histogram implicit solvent code : DMF (light), t-butanol (medium), propanol (dark). Although all structures were minimized in the three solvents, the choice was made to display minimized structures in the most dissociative one (DMF).

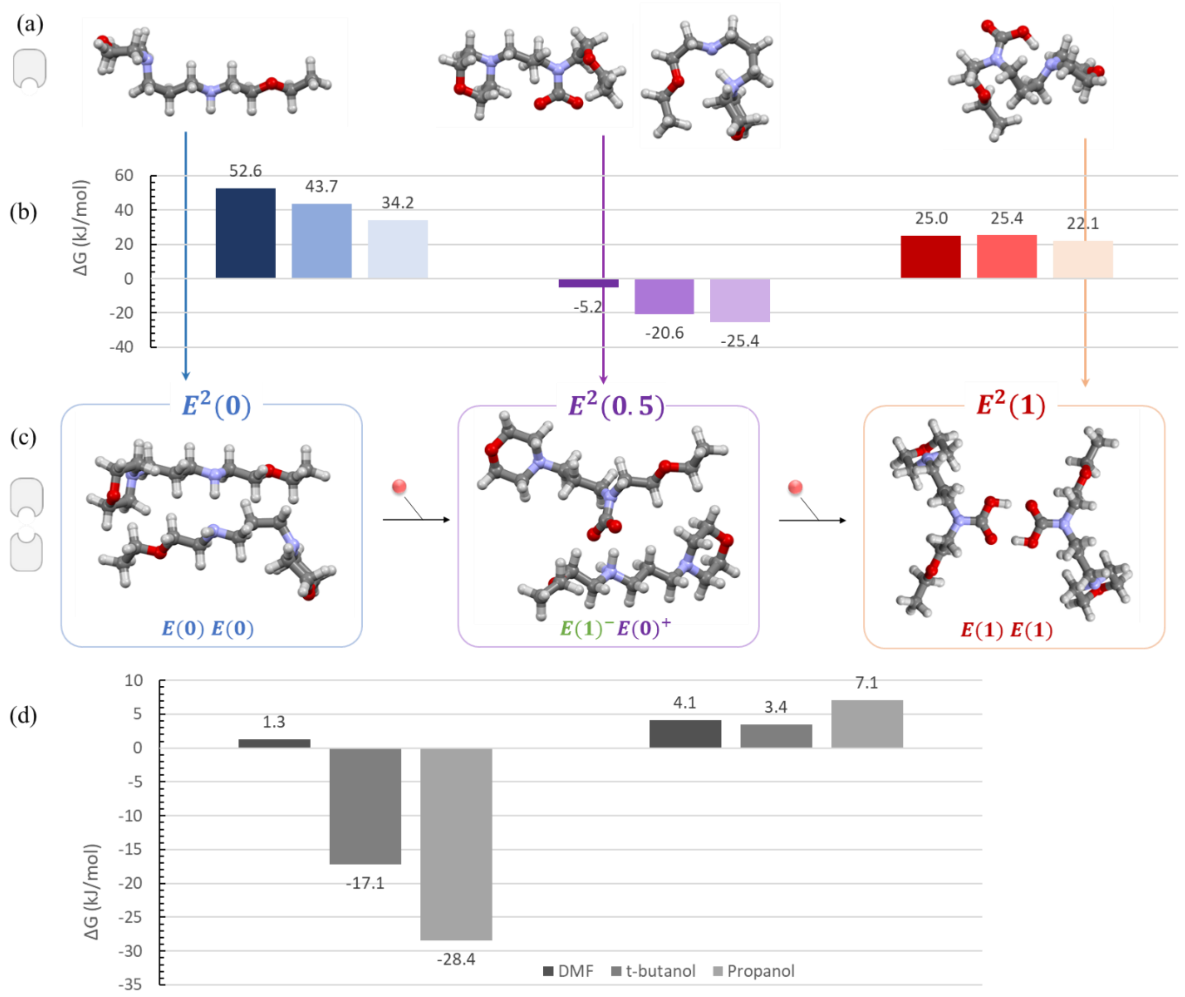

Supplementary Fig. 34. DFT modelling of EEMPA-CO_2 adducts and absorption Gibb's free energies. a) optimized structures of monomeric constituents (in DMF) and their dimerization Gibb's free energies (b) into corresponding optimized structures (c, in DMF) and successive Gibb's free energies of CO_2 absorption (d) (Atom color code : C (black), H (white), O (red), N (blue); histogram implicit solvent code : DMF (light), t-butanol (medium), propanol (dark). Although all structures were minimized in the three solvents, the choice was made to display minimized structures in the most dissociative one (DMF).

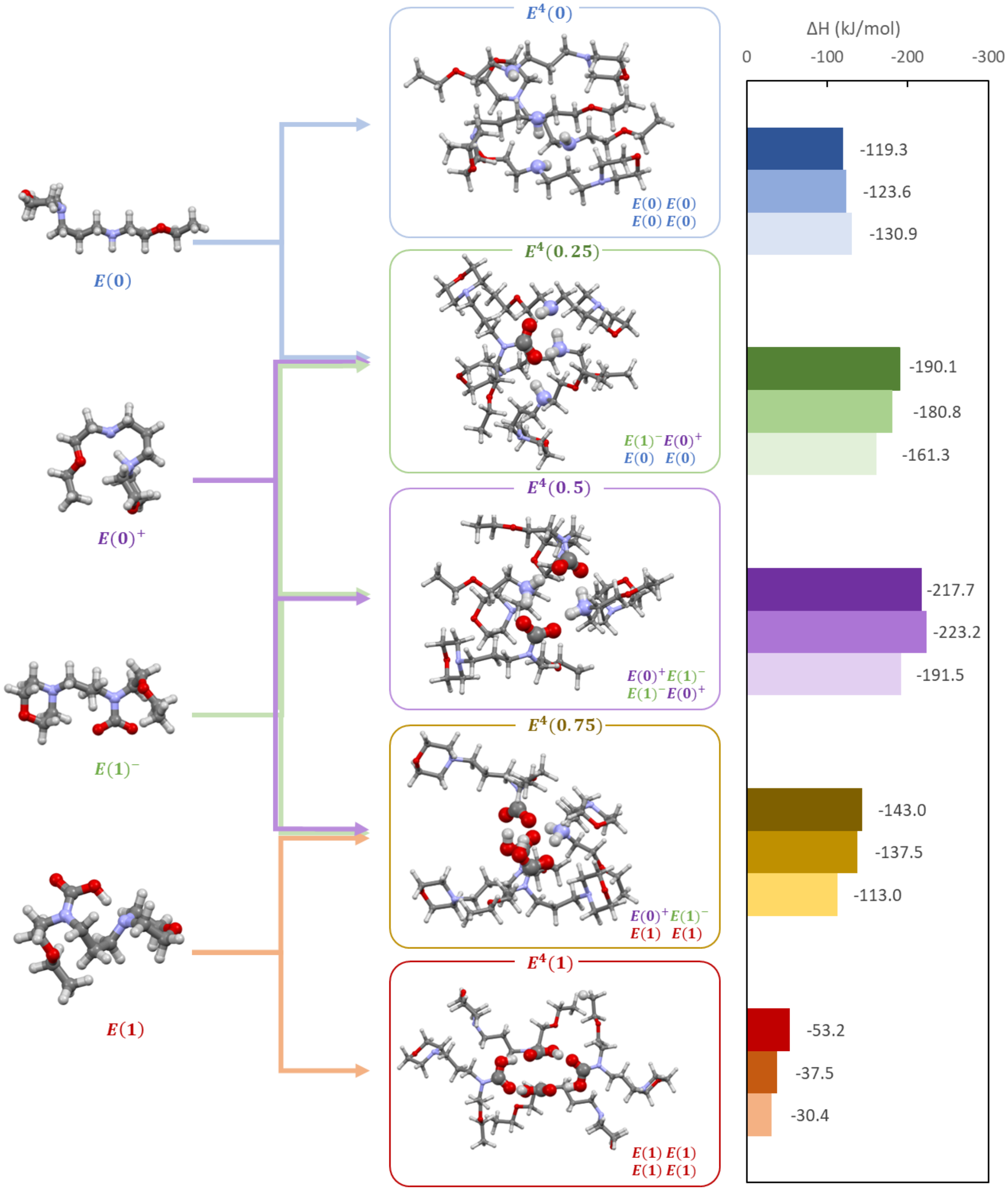

Supplementary Fig. 35. DFT modelling of EEMPA-CO_2 adducts and tetramerization enthalpies. Optimized structures of monomeric constituents (in DMF, left) and their tetramerization enthalpy (right) into corresponding optimized structures (in DMF, middle) (Atom color code : C (black), H (white), O (red), N (blue); histogram implicit solvent code : DMF (light), t-butanol (medium), propanol (dark). Although all structures were minimized in the three solvents, the choice was made to display minimized structures in the most dissociative one (DMF).

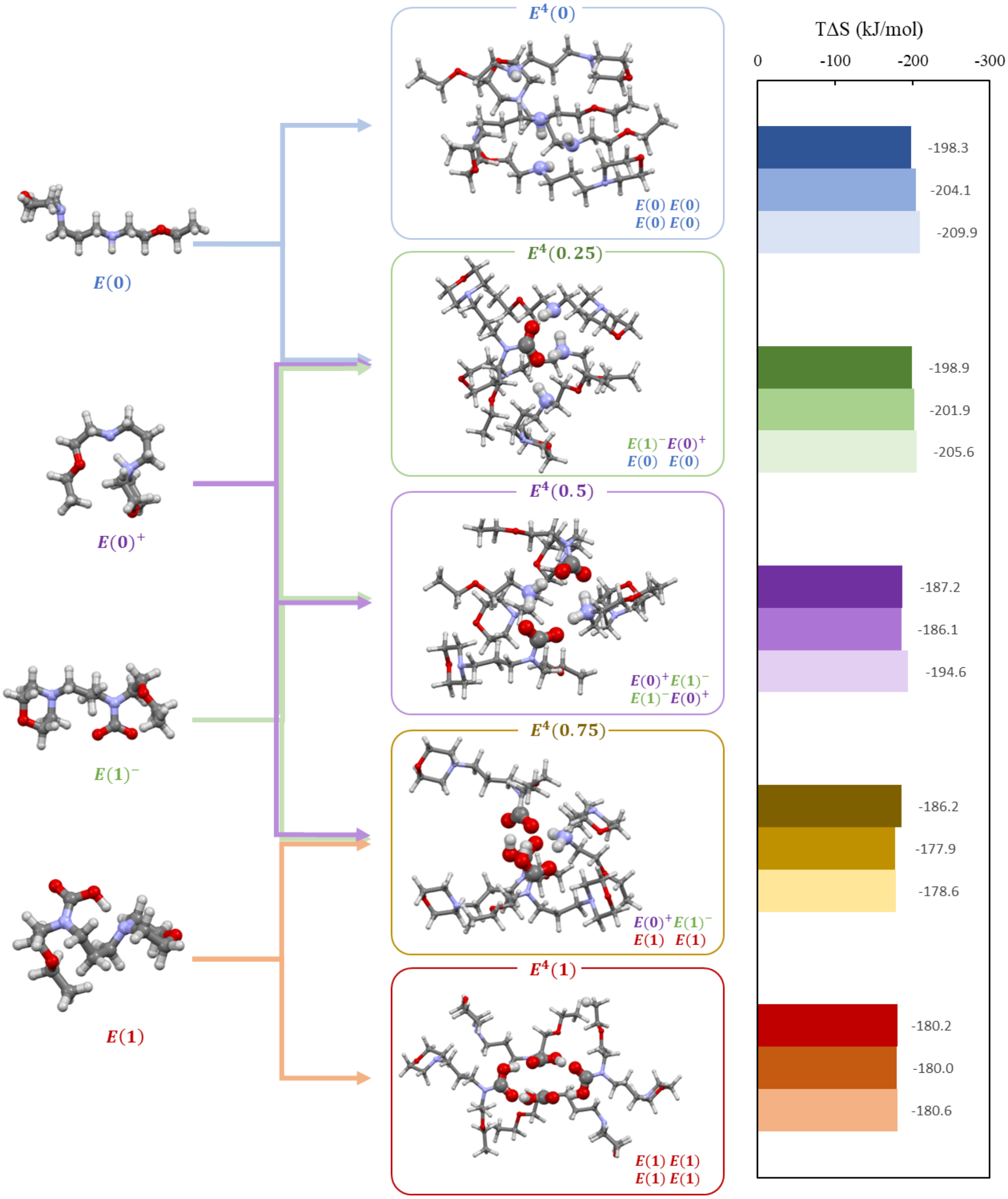

Supplementary Fig. 36. DFT modelling of EEMPA-CO_2 adducts and tetramerization entropies. Optimized structures of monomeric constituents (in DMF, left) and their tetramerization entropy (right) into corresponding optimized structures (in DMF, middle) (Atom color code : C (black), H (white), O (red), N (blue); histogram implicit solvent code : DMF (light), t-butanol (medium), propanol (dark). Although all structures were minimized in the three solvents, the choice was made to display minimized structures in the most dissociative one (DMF).

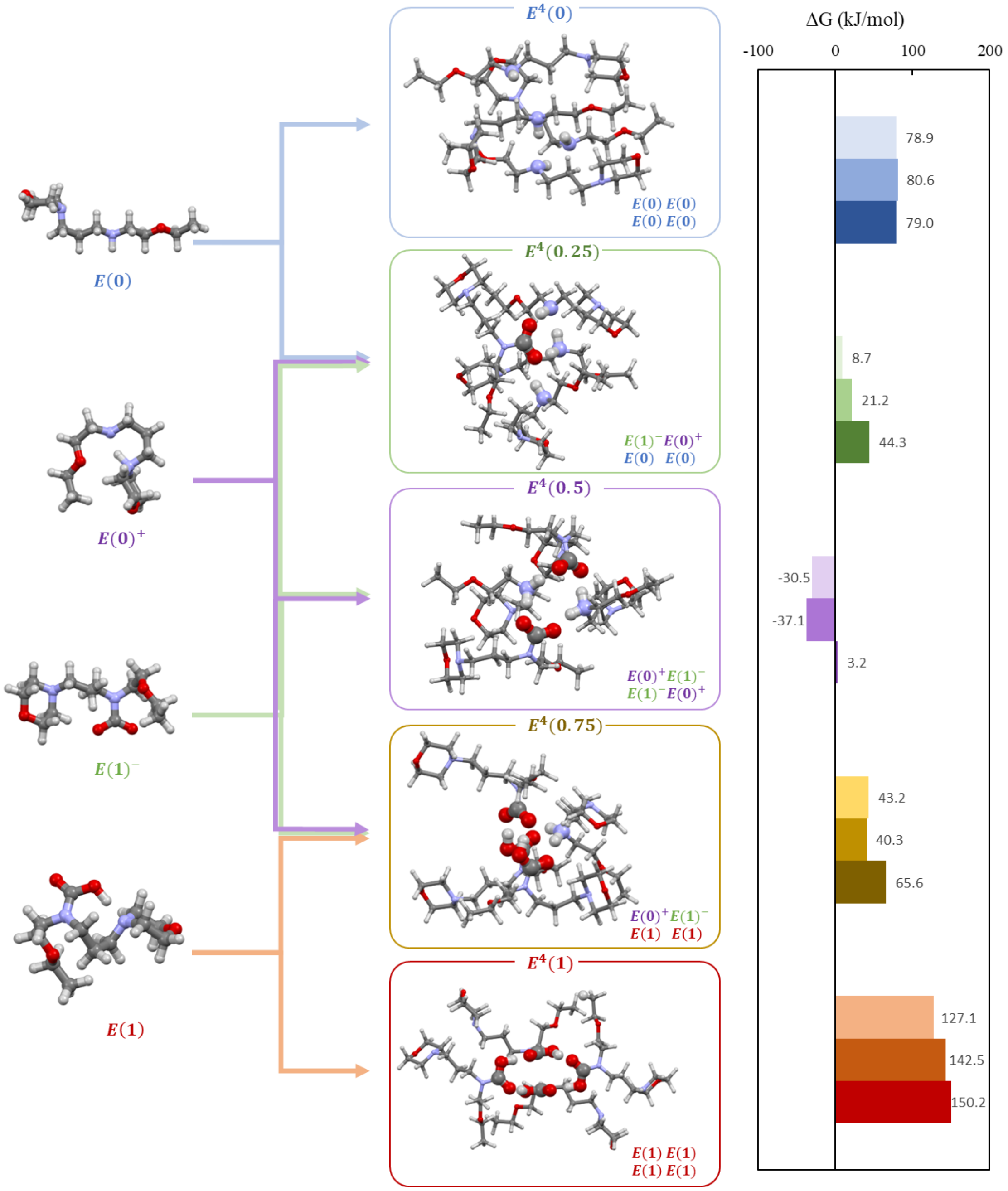

Supplementary Fig. 37. DFT modelling of EEMPA-CO_2 adducts and tetramerization Gibb's free energies. Optimized structures of monomeric constituents (in DMF, left) and their tetramerization Gibb's free energies (right) into corresponding optimized structures (in DMF, middle) (Atom color code : C (black), H (white), O (red), N (blue); histogram implicit solvent code : DMF (light), t-butanol (medium), propanol (dark). Although all structures were minimized in the three solvents, the choice was made to display minimized structures in the most dissociative one (DMF).

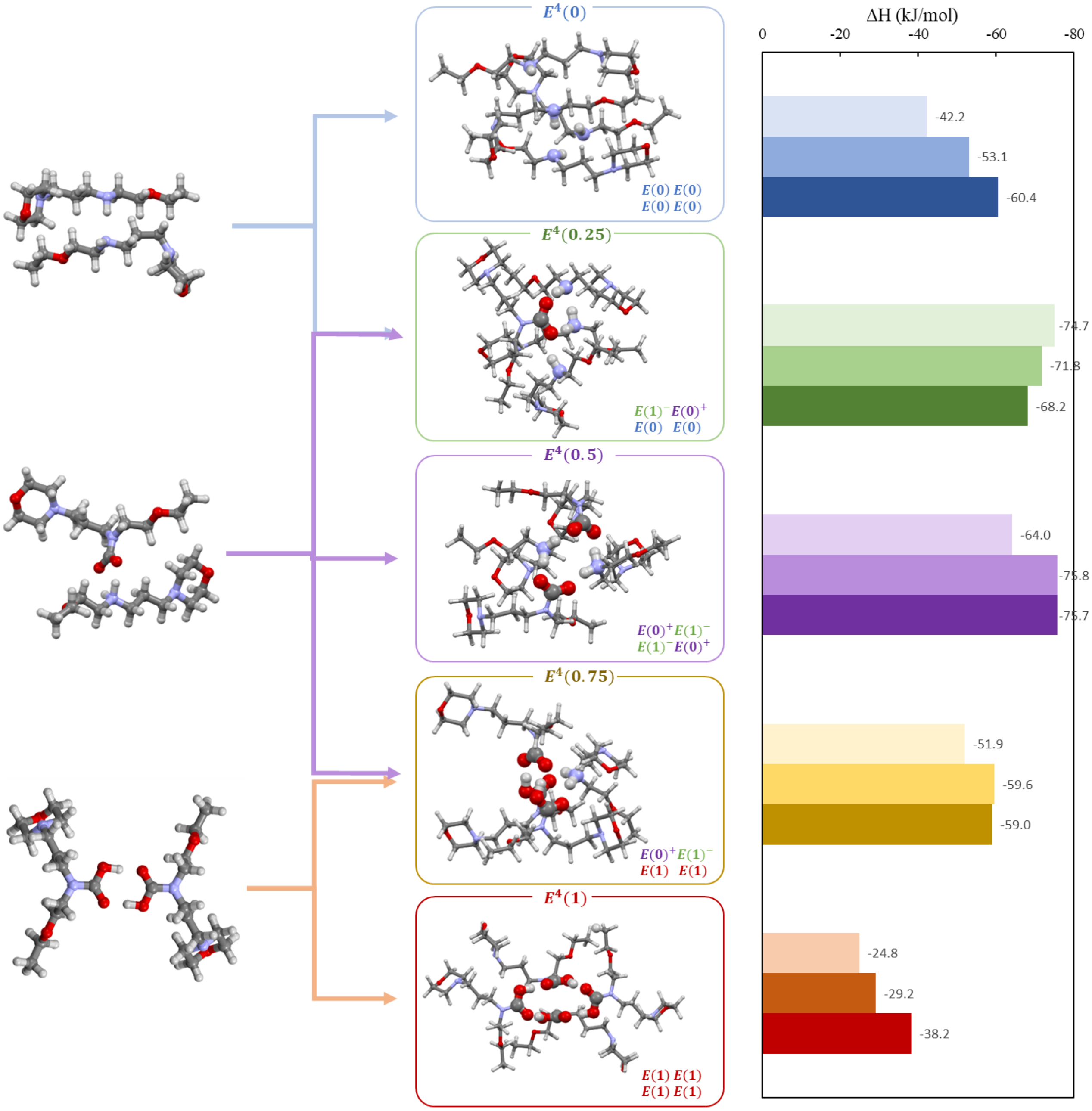

Supplementary Fig. 38. DFT modelling of EEMPA-CO_2 adducts and dimerization enthalpies. Optimized structures of dimeric constituents (in DMF, left) and their dimerization enthalpies (right) into corresponding optimized tetrameric structures (in DMF, middle) (Atom color code : C (black), H (white), O (red), N (blue); histogram implicit solvent code : DMF (light), t-butanol (medium), propanol (dark). Although all structures were minimized in the three solvents, the choice was made to display minimized structures in the most dissociative one (DMF).

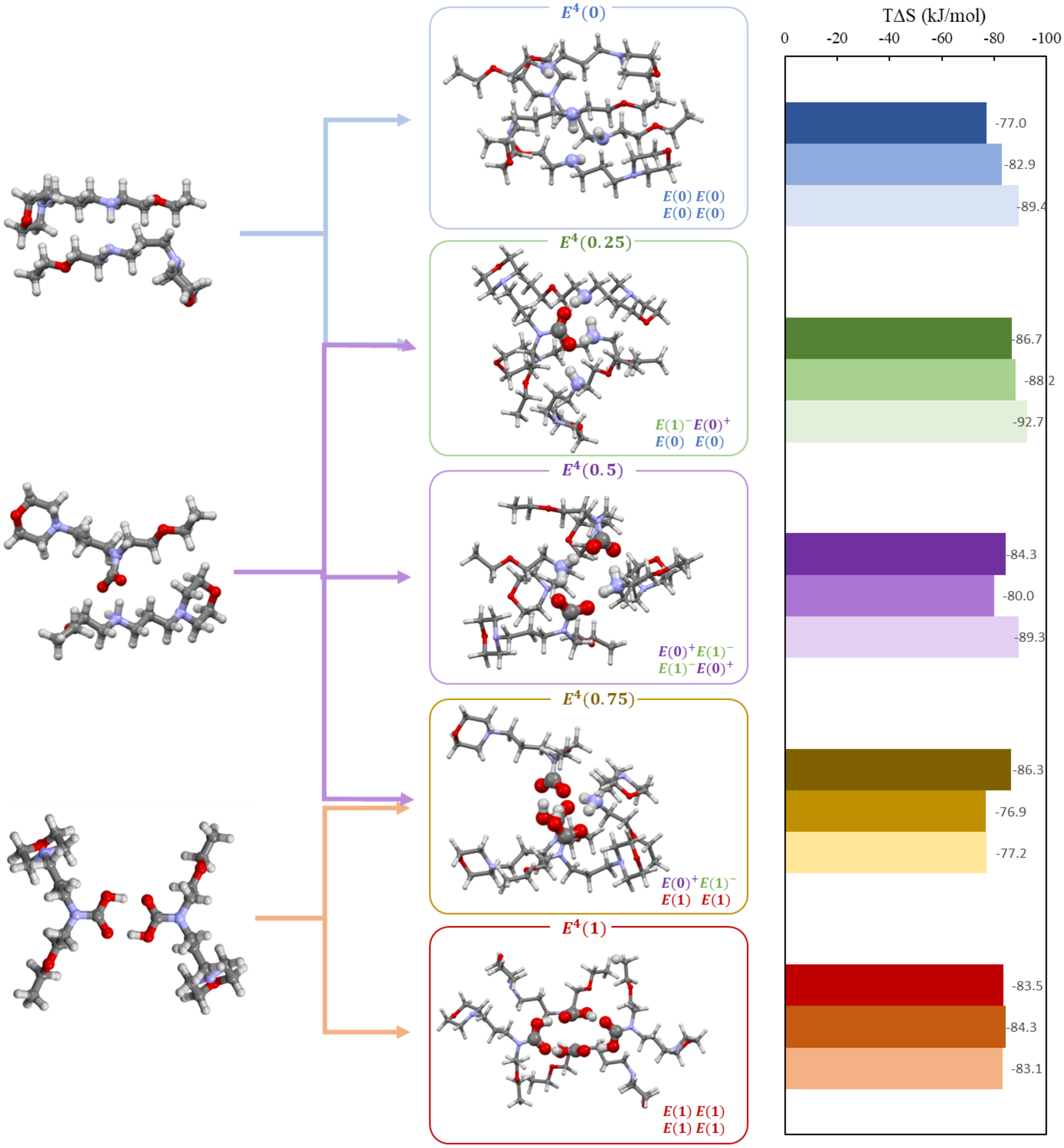

Supplementary Fig. 39. DFT modelling of EEMPA-CO_2 adducts and dimerization entropies. Optimized structures of dimeric constituents (in DMF, left) and their dimerization entropies (right) into corresponding optimized tetrameric structures (in DMF, middle) (Atom color code : C (black), H (white), O (red), N (blue); histogram implicit solvent code : DMF (light), t-butanol (medium), propanol (dark). Although all structures were minimized in the three solvents, the choice was made to display minimized structures in the most dissociative one (DMF).

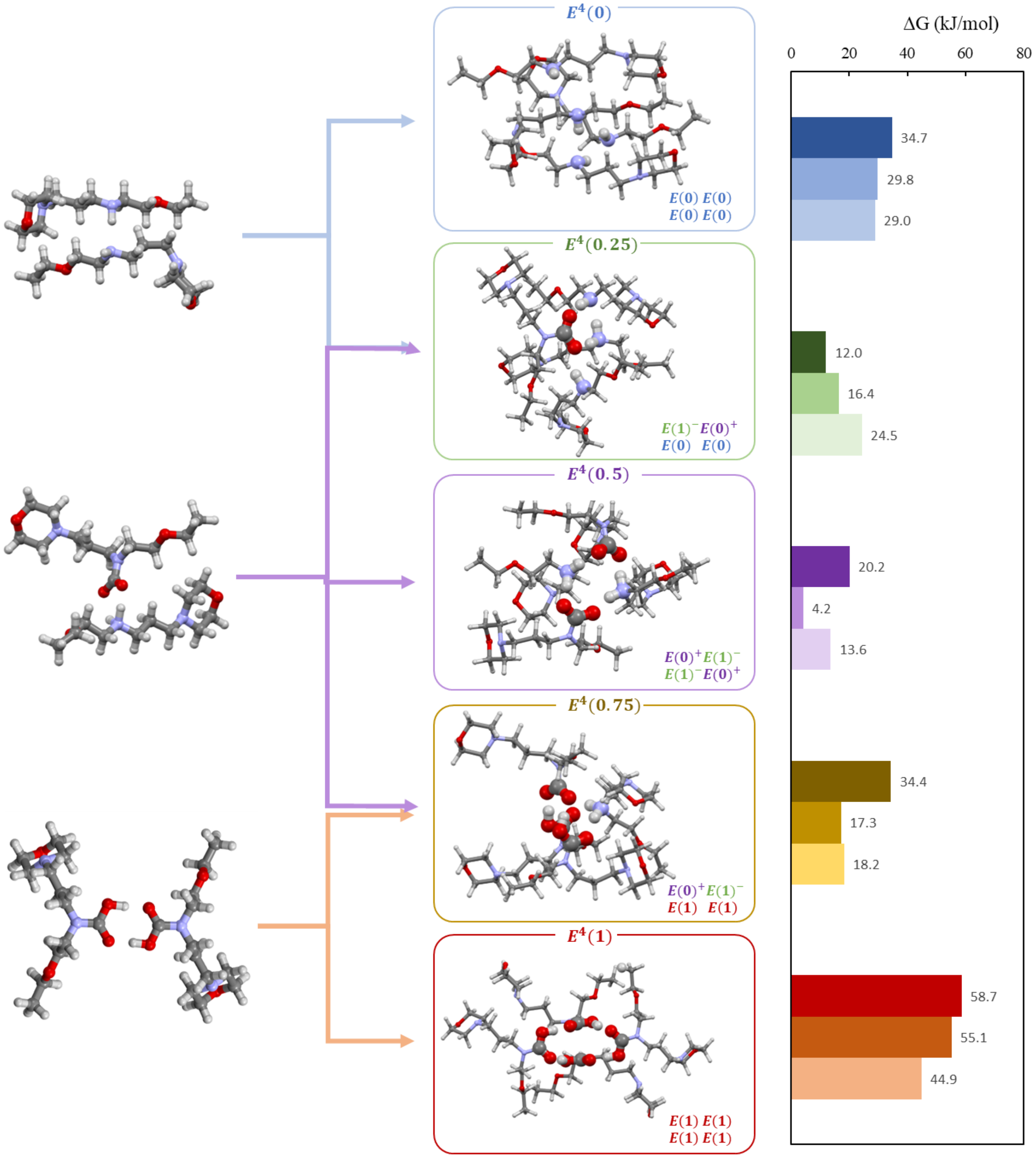

Supplementary Fig. 40. DFT modelling of EEMPA-CO_2 adducts and dimerization Gibb's free energies. Optimized structures of dimeric constituents (in DMF, left) and their dimerization Gibb's free energies (right) into corresponding optimized tetrameric structures (in DMF, middle) (Atom color code : C (black), H (white), O (red), N (blue); histogram implicit solvent code : DMF (light), t-butanol (medium), propanol (dark). Although all structures were minimized in the three solvents, the choice was made to display minimized structures in the most dissociative one (DMF).

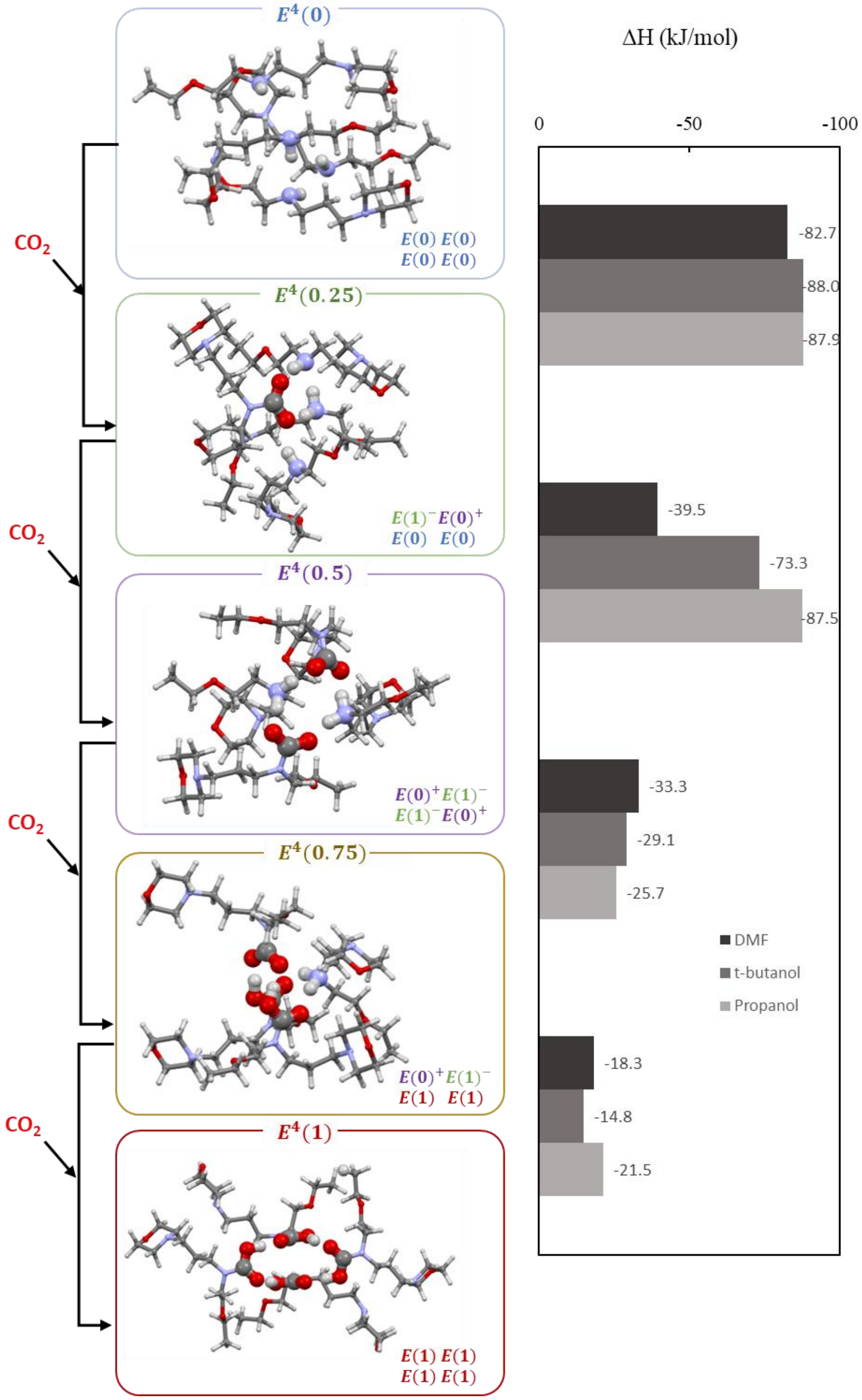

Supplementary Fig. 41. DFT modelling of CO_2 absorption by self-assembled tetramers. Optimized structures of monomeric constituents (in DMF, left) and their CO_2 absorption enthalpy (right) (Atom color code : C (black), H (white), O (red), N (blue); histogram implicit solvent code : DMF (dark), t-butanol (medium), propanol (light). Although all structures were minimized in the three solvents, the choice was made to display minimized structures in the most dissociative one (DMF).

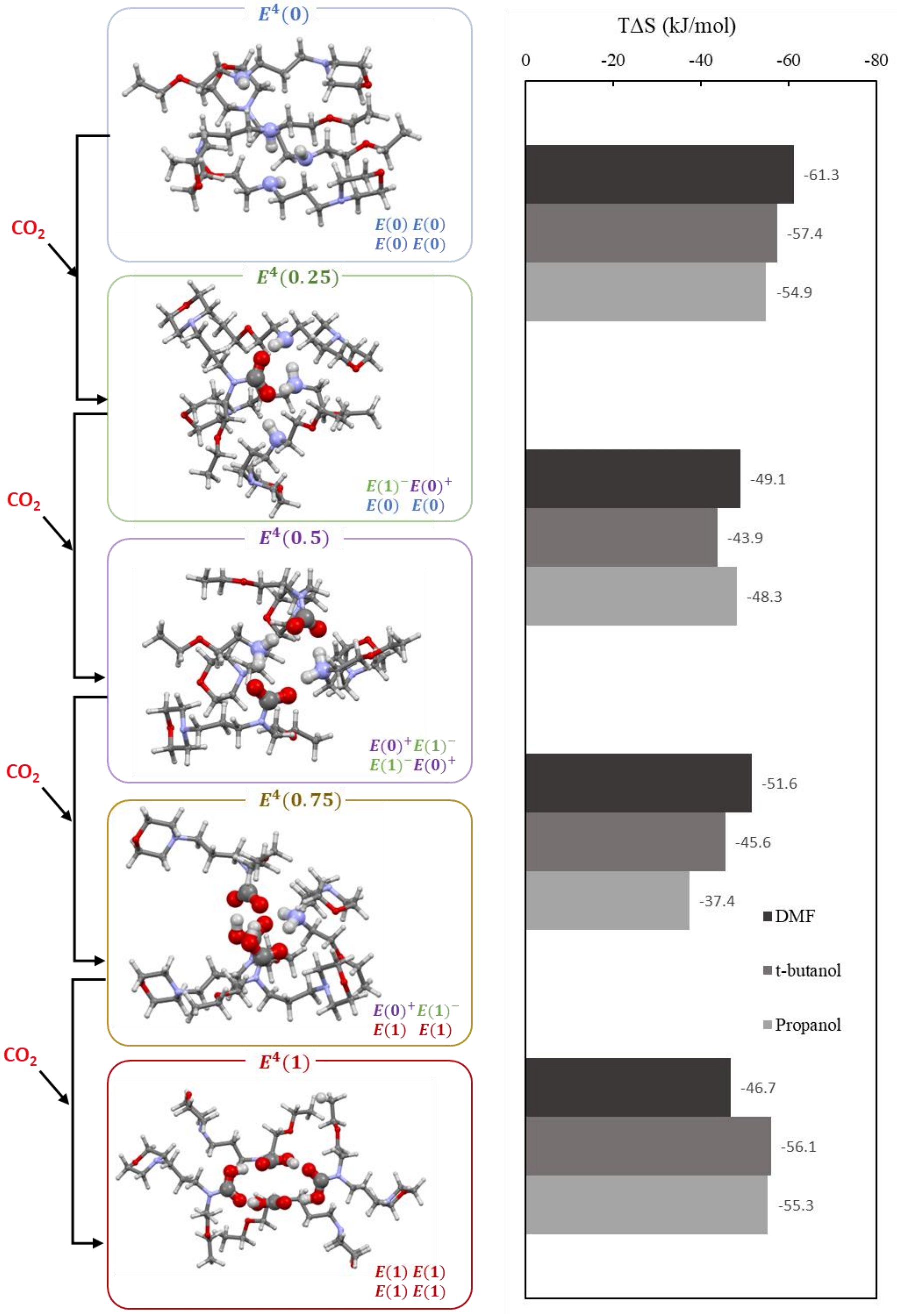

Supplementary Fig. 42. DFT modelling of CO_2 absorption by self-assembled tetramers. Optimized structures of monomeric constituents (in DMF, left) and their CO_2 absorption entropies (right) (Atom color code : C (black), H (white), O (red), N (blue); histogram implicit solvent code : DMF (light), t-butanol (medium), propanol (dark). Although all structures were minimized in the three solvents, the choice was made to display minimized structures in the most dissociative one (DMF).

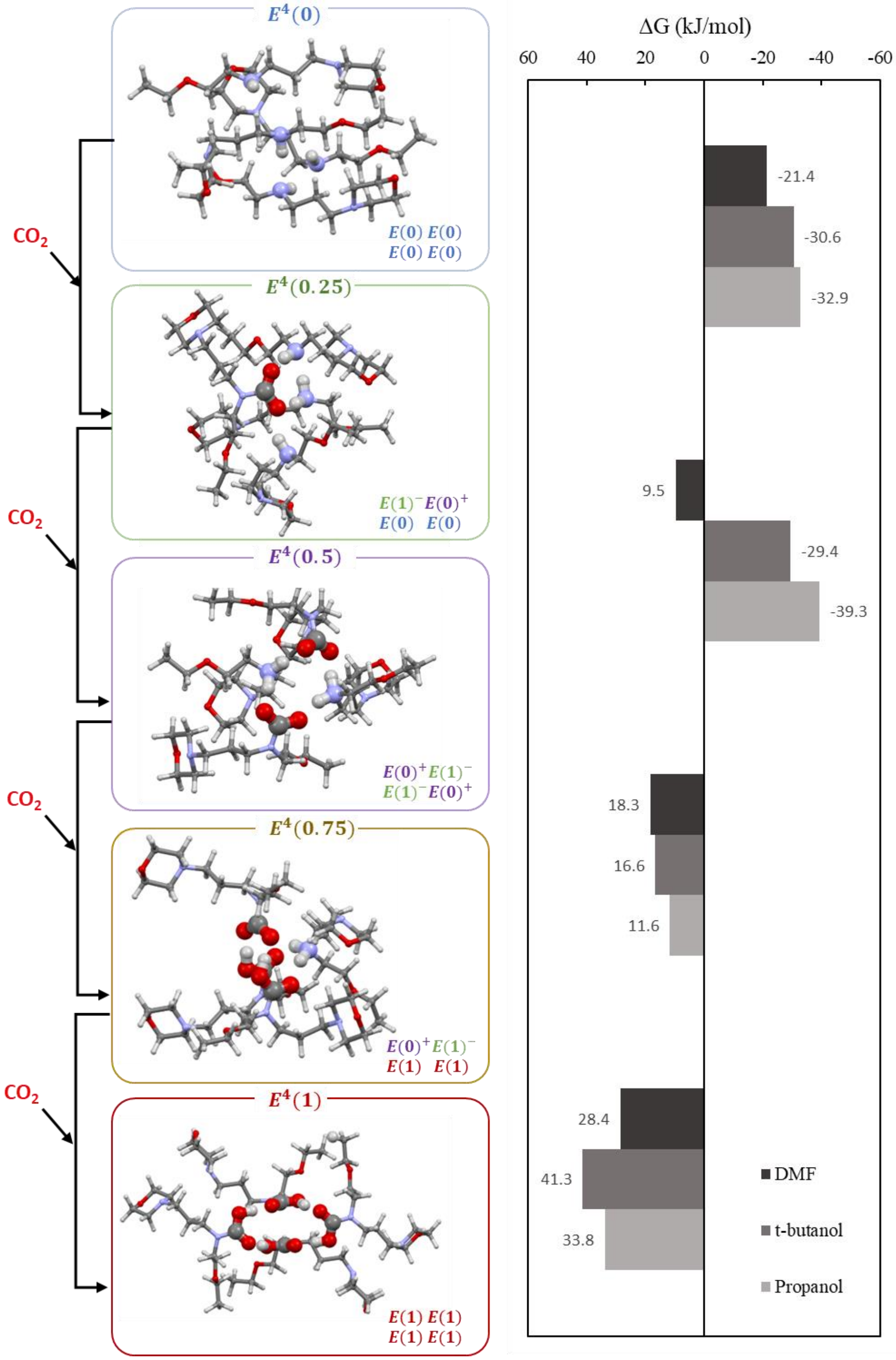

Supplementary Fig. 43. DFT modelling of CO_2 absorption by self-assembled tetramers. Optimized structures of monomeric constituents (in DMF, left) and their CO_2 absorption Gibb's free energy (right) (Atom color code : C (black), H (white), O (red), N (blue); histogram implicit solvent code : DMF (light), t-butanol (medium), propanol (dark). Although all structures were minimized in the three solvents, the choice was made to display minimized structures in the most dissociative one (DMF).

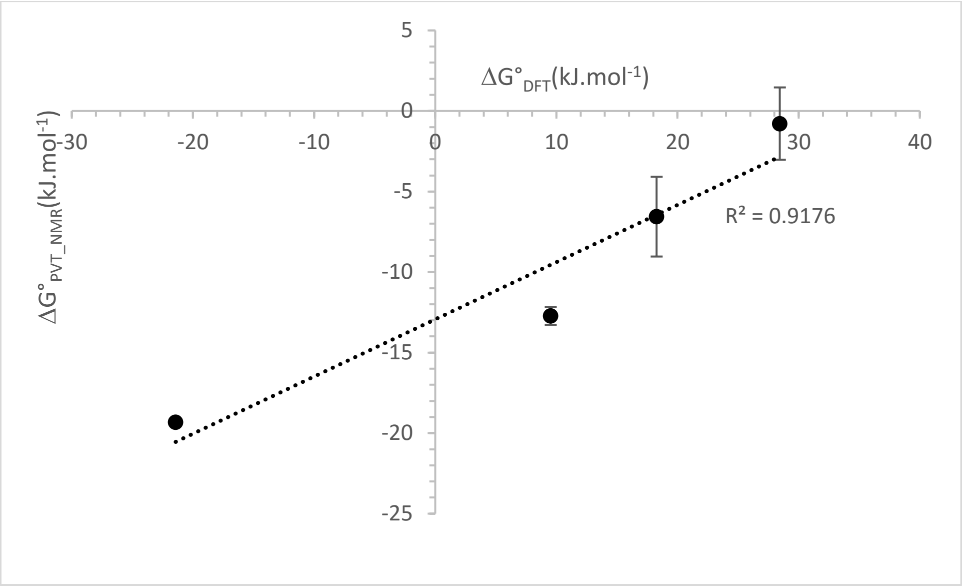

Supplementary Fig. 44. Plot of the Gibb's free energies derived from NMR and PVT measurements vs DFT-computed Gibb's free energies of CO_2 absorption by the four different tetramers **E^4(0), E^4(0.25), E^4(0.5)** and **E^4(0.75).** Error bars correspond to the standard deviation originating from the $q^{13}C$ integrations of 4 peaks of the monomeric components of the tetramers.

Structural data from DFT calculations

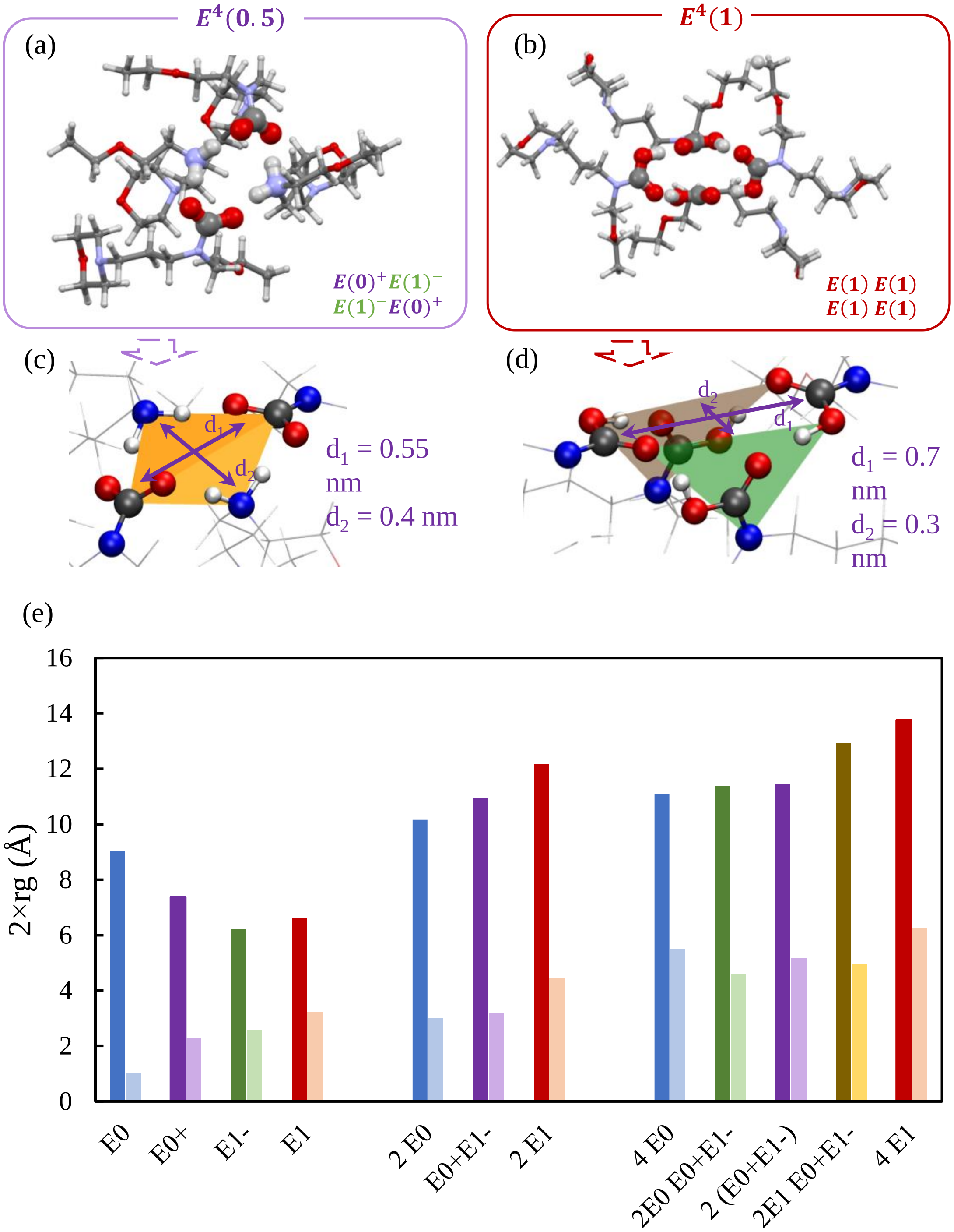

Supplementary Fig. 45. DFT modelling of self-assembled tetramers in DMF and quantification of their morphological features. Minimized structures of $\mathbf{E^4(0.5)}$ (a), $\mathbf{E^4(1)}$ (b) and of their internal reactive sites (c) and (d). These dimension as well as the computed sizes of tetramer clusters (left bars) and their cavities (right bars) quantified by gyradius (e) match experimental values obtained by WAXS (see **Supplementary Fig. 30**). (atom color code : blue : nitrogen, red : oxygen; black : carbon; white : hydrogen).

cMD data for Tetrameric Structures as a function of time at 25% CO_2 loading

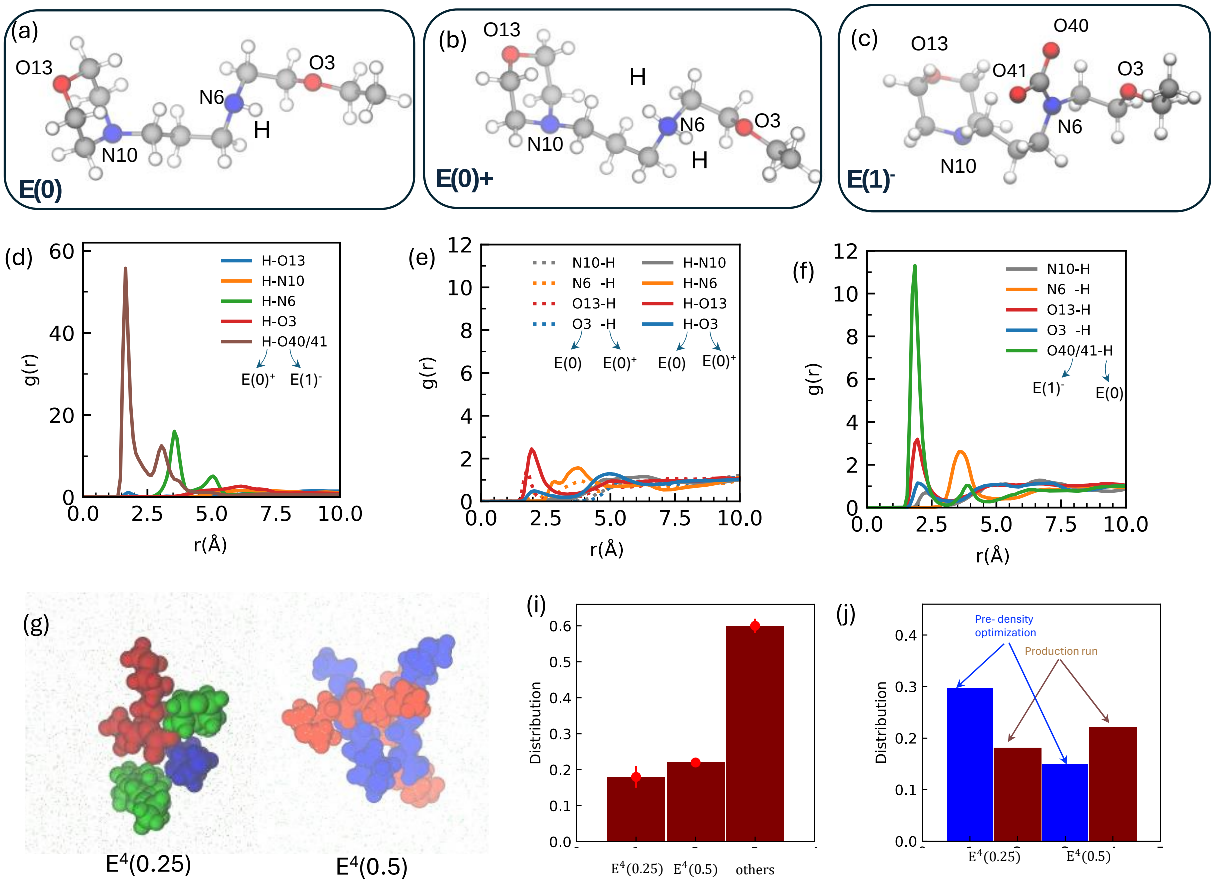

Supplementary Fig. 46. (a-c) Native **E(0)**, **E(0)$^+$** and **E(1)$^-$** monomeric constituents. (d-f): radial distribution functions of atom pairs in these three monomers. (g) example of **E^4(0.25)** and **E^4(0.5)** observed during cMD simulations, with **E(0)** in green, **E(0)$^+$** in blue, and **E(1)$^-$** in red. (i) Distribution of molecules in clusters from production run. (j) Comparison of the distribution of molecules in these clusters in the preparation stage and production run stage (see Method section). Simulations were run at 298K.

Our classical molecular dynamics simulations support the tetramerization during unpressurized CO_2 loading. Data shown in panels (a-f) confirm that the hydrogen bonds between pairs of atoms can be used to define tetrameric clusters. The following condition was used: if the distance between two atoms (for example, H and O40/41 (panel (b-d)) of two H-bonded molecules is within the position of the first minimum of the corresponding radial distribution function, then these two molecules considered to be part of a cluster. For **E^4(0.5)**, very strong hydrogen bonds between (N)H_2^+ and (C)O_2- (panel (d)) lead to well-defined clusters. In contrast, **E^4(0.25)** consists of a pair of **E(0)$^+$** and **E(1)$^-$** molecules and a number of surrounding **E(0),** which could be determined based on hydrogen bonds between the H atom of **E(0)** and the (C)O_2^- moiety of **E(1)$^-$.** Much weaker hydrogen bonds between H of (**E(0)**) and O13 of (**E(1)$^-$, E(0)$^+$**) were also considered. Examples of **E^4(0.25)** and **E^4(0.5)** are shown in panel (g). On average (statistics on the full box and the 1

microsecond timeframe), there are approximately 1.4 **E(0)** molecules in the surrounding of an **E(0)**$^{+}$ **- E(1)**$^{-}$ pair, which correspond to a significant proportion of **E**^{4}**(0.25)** tetramers. By applying the restrictive criteria of a lifetime superior to 1 microsecond, we found that the percentages of **E**^{4}**(0.25)** and **E**^{4}**(0.5)** in the simulation box along the trajectory are ~18 (%) and 22 (%), panel (i). To be considered in the **E**^{4}**(0.5)** statistics, a [2**E(0)**$^{+}$ + **2E(1)**$^{-}$] system had to fulfill the restrictive criteria : to be internally connected by 4 H-O40/O41 bonds, and not to bind any other **E** monomer through these moieties along the full trajectory. To be considered in the **E**^{4}**(0.25)** statistics, the **2E(0)**, **E(0)**$^{+}$, and **E(1)**$^{-}$ components had to fulfill the slightly less restrictive criteria of of forming a **E(0)**$^{+}$ **- E(1)**$^{-}$ core (through H-O40/O41 bonds), and this core dimer had to be bounded to a varying number of **E(0)** molecules. The error bars in the resulting populations displayed at panel (i) are of about +/- 2%. These were calculated as the deviation of the values from ten 100-ns trajectories (making up the full 1-microsecond trajectory) from the average value of the full 1-microsecond trajectory. We also note that these percentages change significantly from a preparation stage to the production run stage, (panel (j)), implying a strong relaxation of the system during the equilibration process (see Method section for different stages of cMD simulations).

^{13}C NMR monitoring of E(2) decay

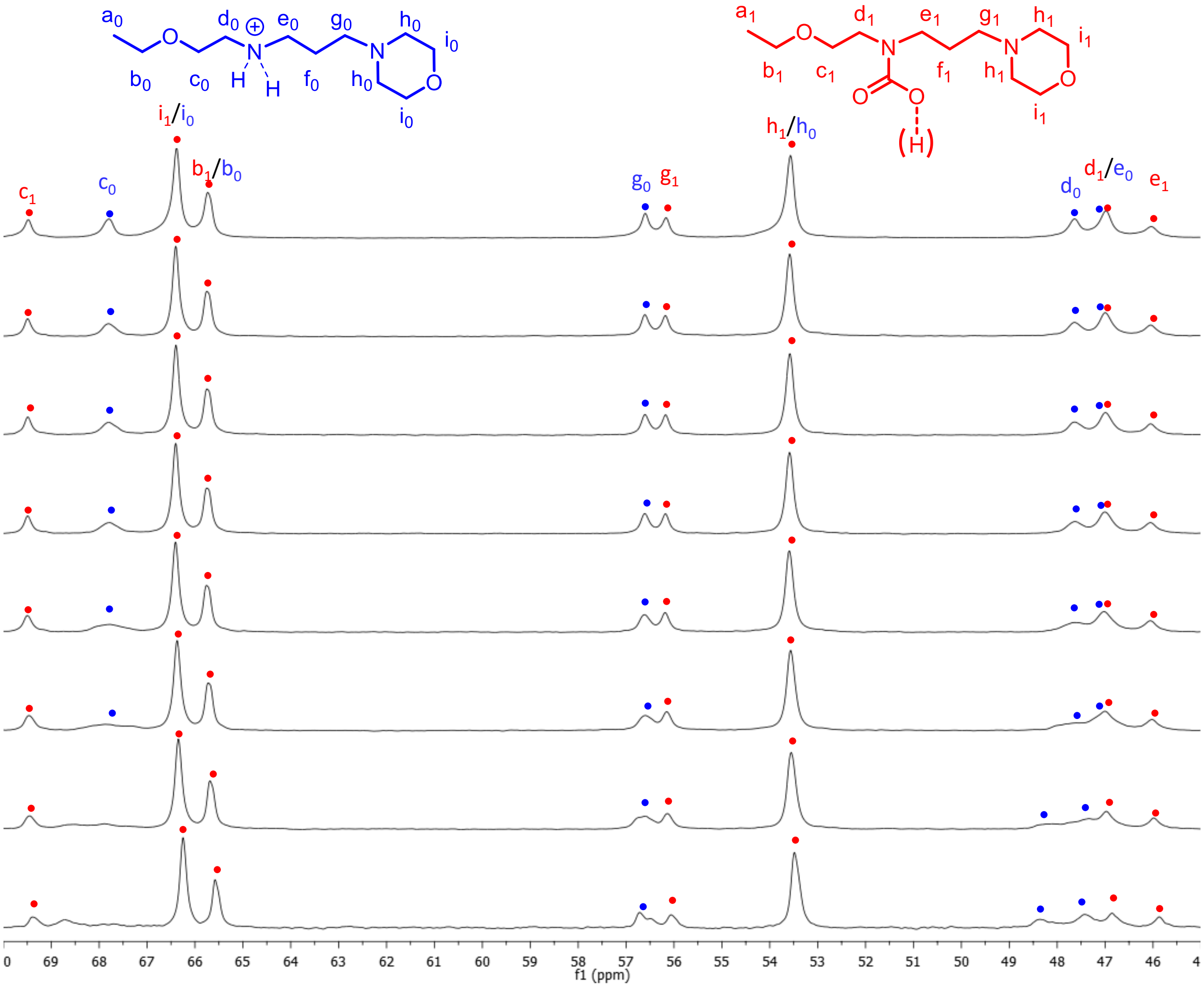

Supplementary Fig. 47. Extension of the 46-72 ppm region of the spectra during decay over time of the free anhydride from $\alpha = 0.96$ (bottom spectrum) to $\alpha = 0.56$ (top spectrum) as measured by relative f1 vs f0 integration. (Bottom spectrum : immediately after transfer, then every 10 min for the next six spectra; the last one was recorded 19h after transfer and correspond to equilibrium state at $\alpha = 0.56$).

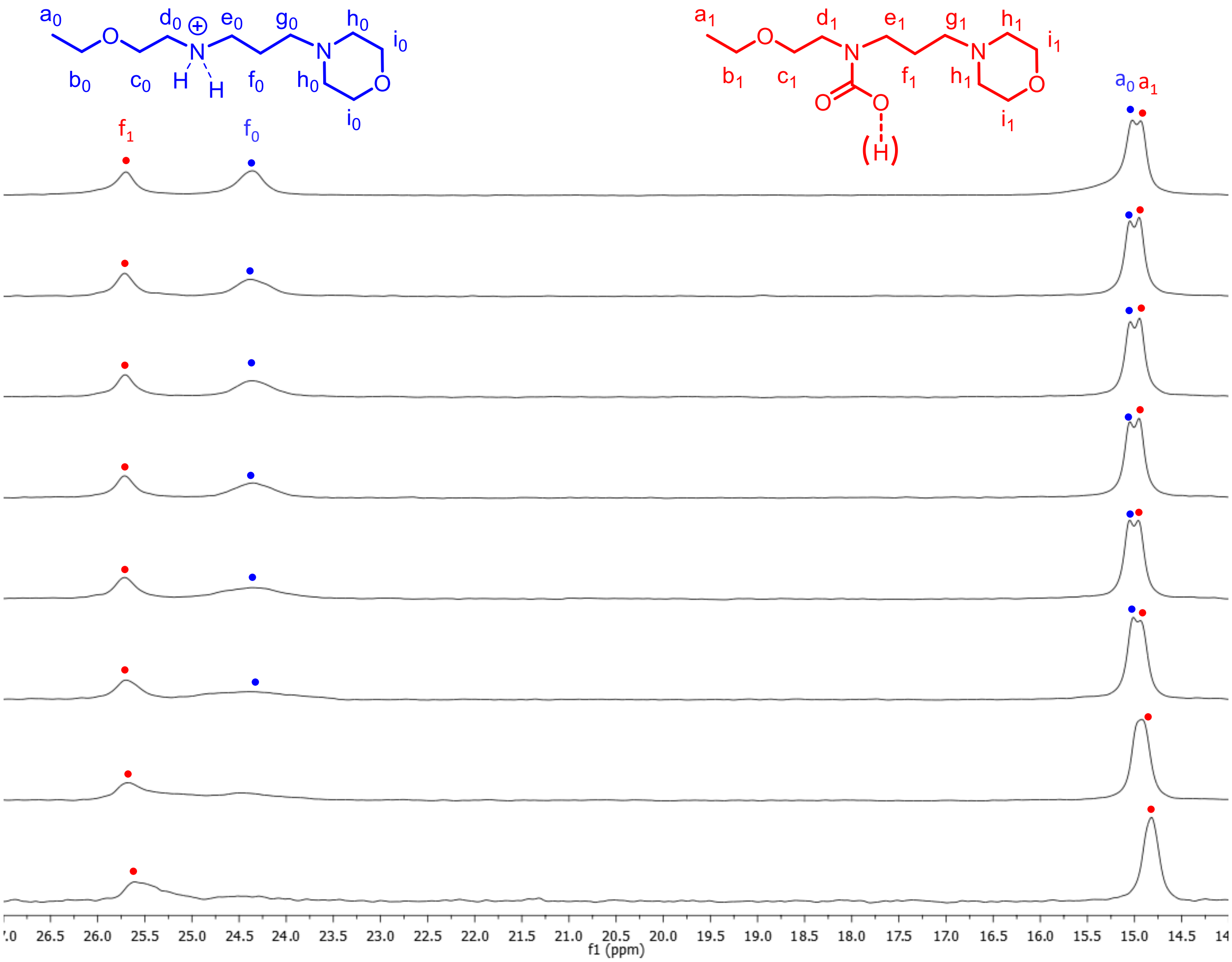

Supplementary Fig. 48.Extension of the 15-28 ppm region of the spectra during decay over time of the free anhydride from α = 0.96 (bottom spectrum) to α = 0.56 (top spectrum) as measured by relative f_1 vs f_0 integration. (Bottom spectrum : immediately after transfer, then every 10 min for the next six spectra; the last one was recorded 19h after transfer and correspond to equilibrium state at α = 0.56).

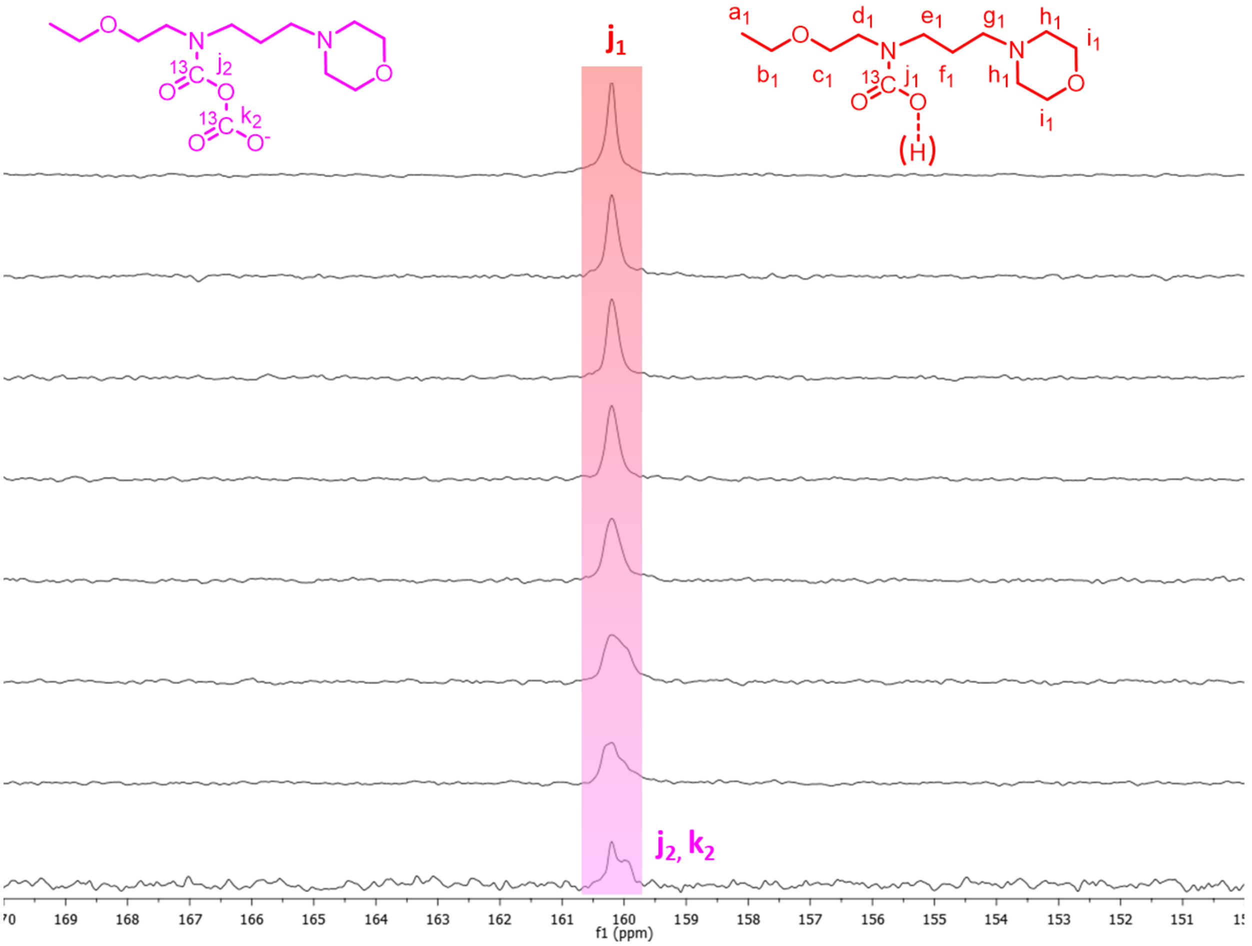

Supplementary Fig. 49. Extension of the 15-28 ppm region of the spectra during decay over time of the free anhydride from α = 0.96 (bottom spectrum) to α = 0.56 (top spectrum) as measured by relative f_1 vs f_0 integration. (Bottom spectrum : immediately after transfer, then every 10 min for the next six spectra; the last one was recorded 19h after transfer and correspond to equilibrium state at α = 0.56).

DFT analysis of CO_2 absorption mechanisms from 50% loading state

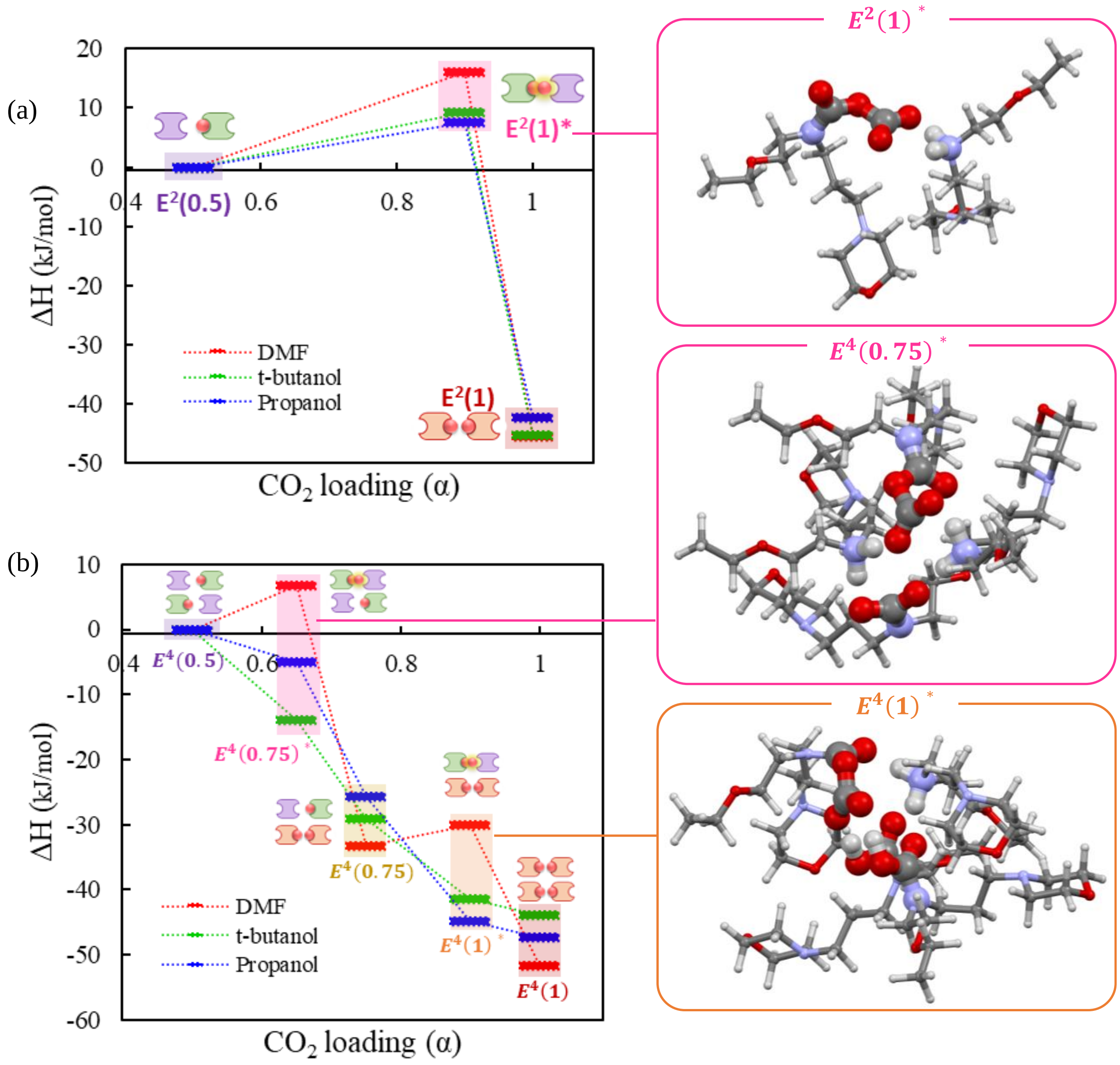

Supplementary Fig. 50. DFT computed anhydride-based CO_2 absorption mechanisms by EEMPA (a) dimeric pathway, (b) tetrameric pathway. Three implicit solvent environments were considered. Structures displayed correspond to DMF as an implicit solvent.

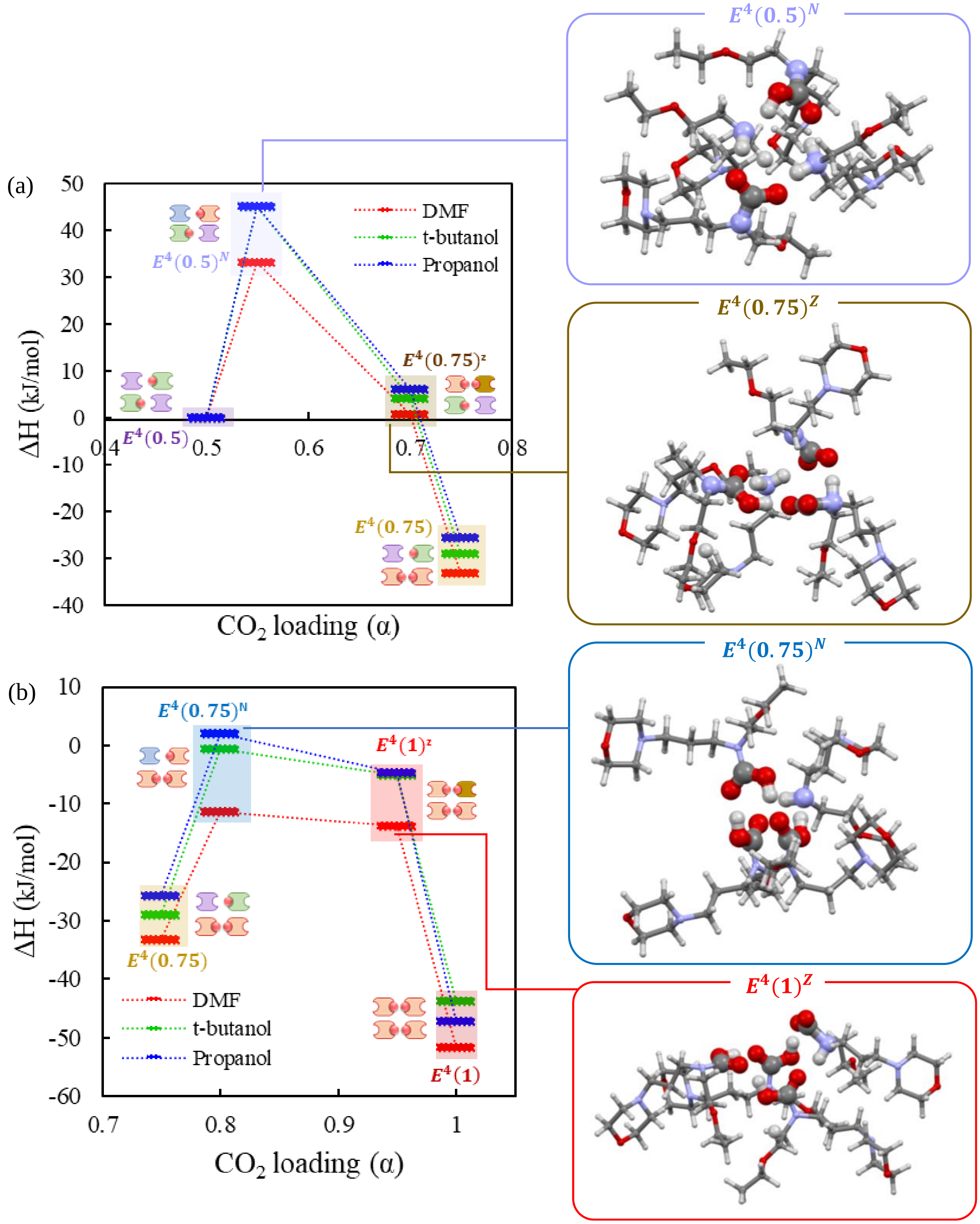

Supplementary Fig. 51. DFT computed zwitterionic mechanisms of E^4(0.5) + CO_2 -> E^4(1) absorption step E^4(0.5) + CO_2 -> **E^4(0.75)** absorption step via the zwitterion intermediate **$E^4(0.75)^z$** (a) and **E^4(0.75)** + CO_2 -> **E^4(1)** absorption step via the zwitterion intermediate **$E^4(1)^z$**. Three implicit solvent environments were considered.

NMR and MS data of in situ and ex situ **E(2)R** formation

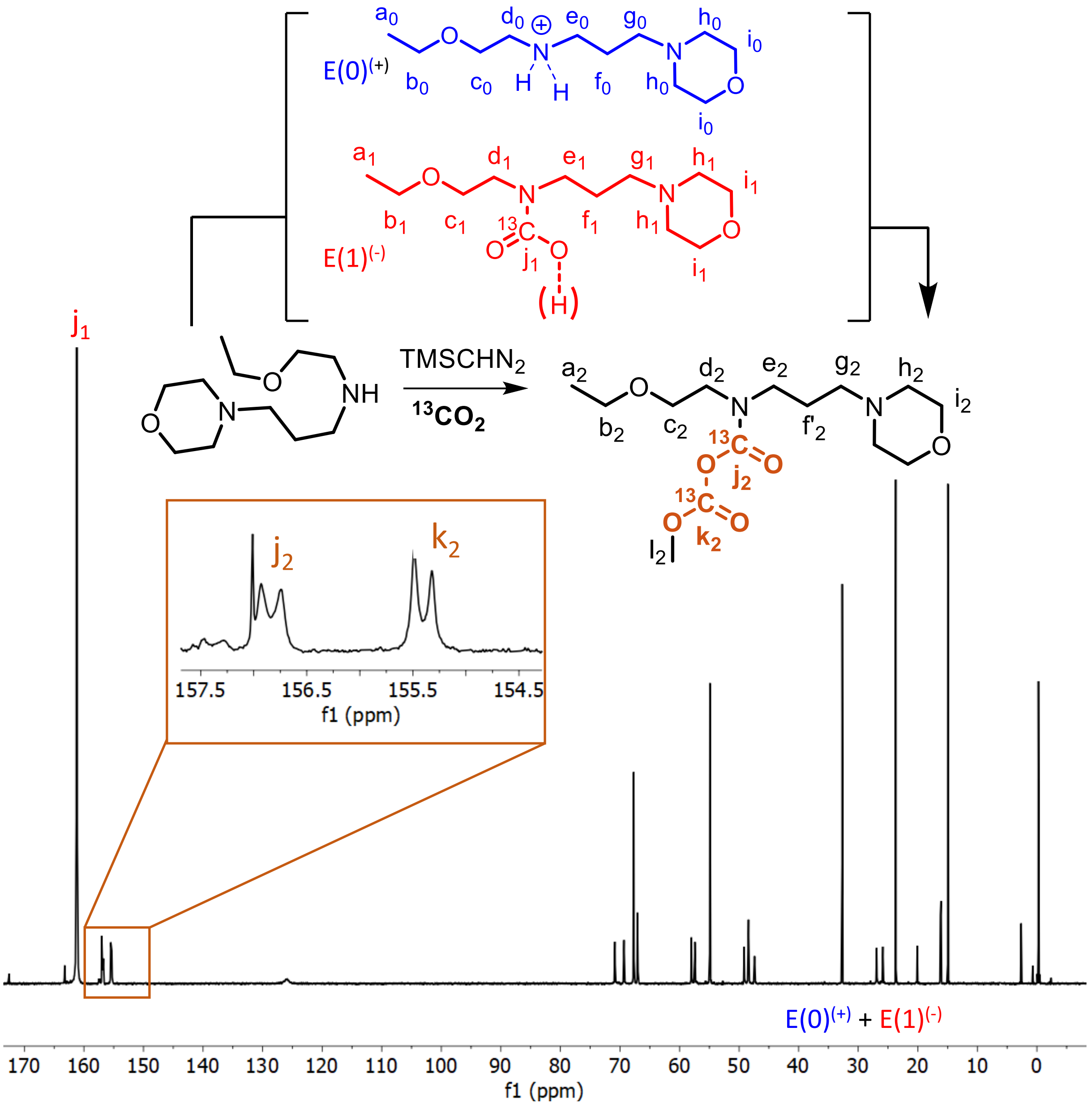

Supplementary Fig. 52. ^{13}C NMR spectra of crude mixture obtained from E, TMSCHN$_2$ (0.5 eq.) and $^{13}CO_2$. See **Supplementary Fig.43** for assignment of the signals below 80 ppm.

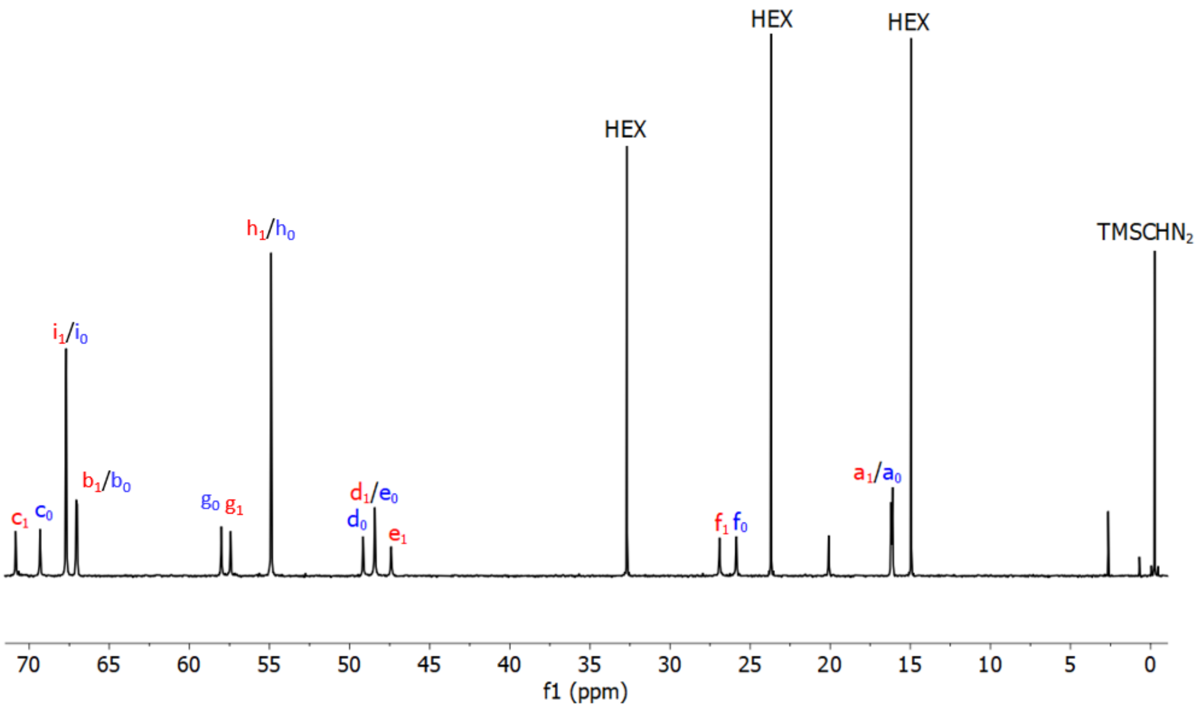

Supplementary Fig. 53. Extension of the aliphatic region with assignments of known signals (**E(1)**$^{(+)}$ and **E(0)**$^{(-)}$ are the major compounds). Signal at 2.6 ppm correspond to the (CH3)$_3$Si of **E-TMS**, main side-product of the reaction when conducted in the absence of MeOH while those at 20 and -1 ppm match those described for $TMSCHN_2$.[55]

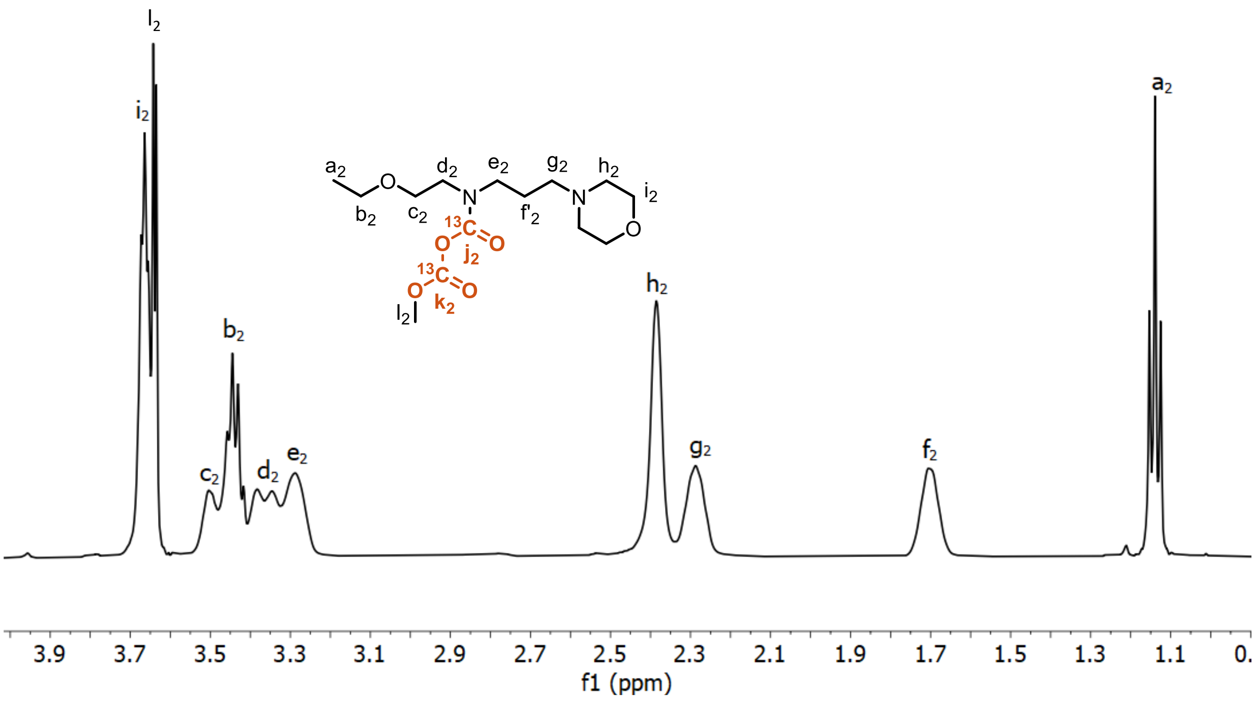

Supplementary Fig. 54.^{1}H NMR of purified **E(2)Me** obtained from $^{13}CO_2$ and $TMSCHN_2$ (1 eq) in the presence of 1 eq. of MeOH. For details about the assignment see **Supplementary Fig. 56.**

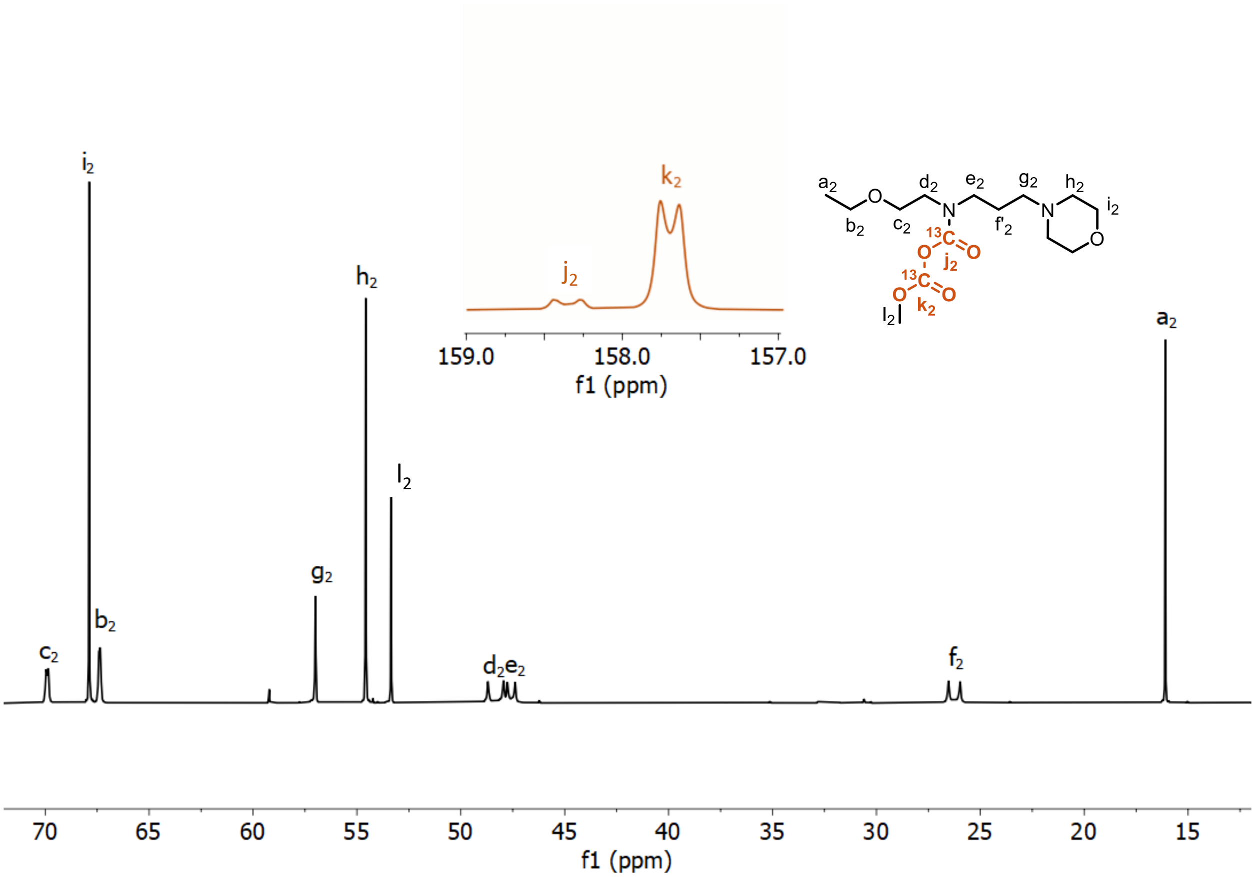

Supplementary Fig. 55. ^{13}C NMR of purified (**E(2)Me**) obtained from $^{13}CO_2$ and TMSCHN$_2$ (1 eq) in the presence of 1 eq. of MeOH, including numbering scheme. Insert is the expansion of the carbonyl region in dark orange.

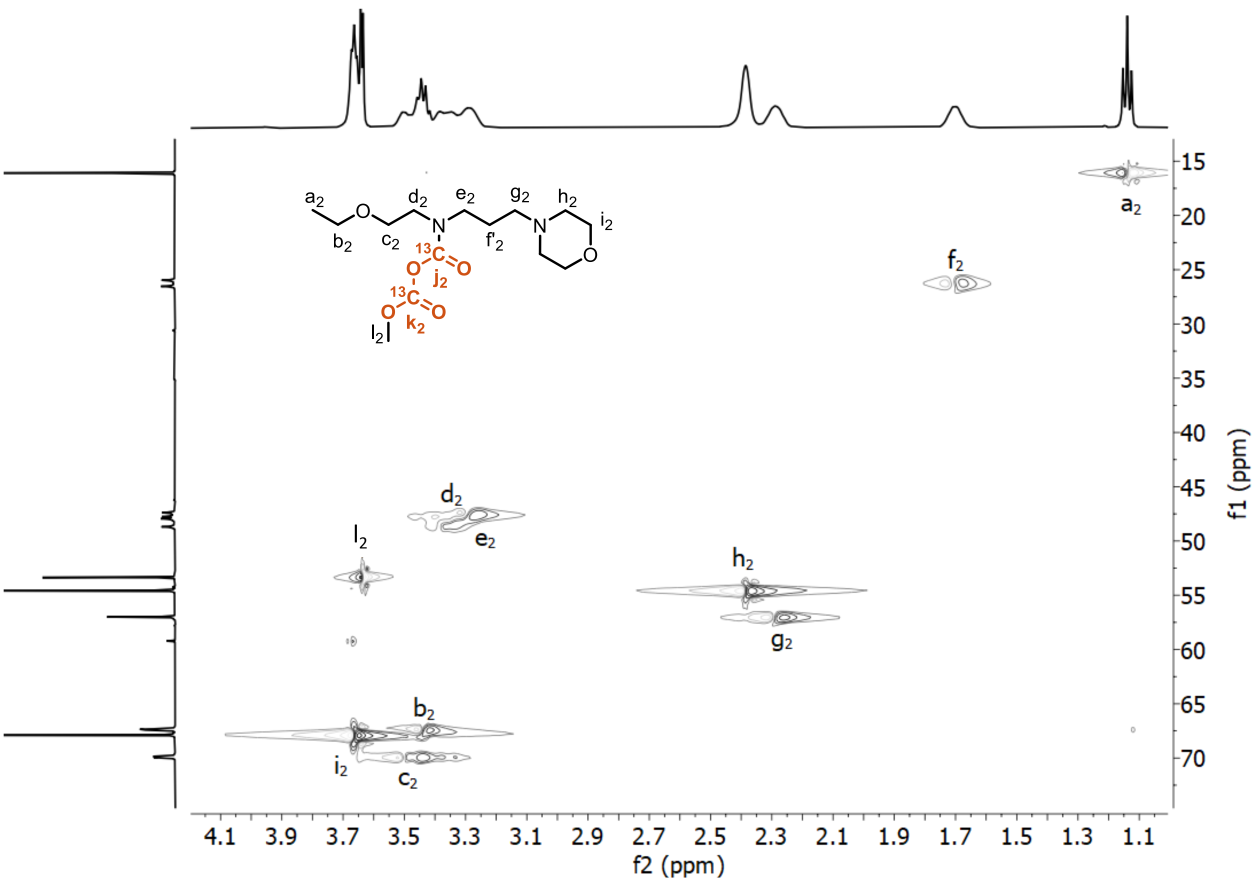

Supplementary Fig. 56. Extension of the relevant region of the HSQC NMR of purified **E(2)Me** obtained from $^{13}CO_2$ and $TMSCHN_2$ (1 eq) in the presence of 1 eq. of MeOH. Numbers relate to protons/carbons as outlined in previous spectra. HSQC was recorded to establish short-range proton-carbon coupling (^1JCH) and assign ^1H signal displayed at **Supplementary Fig. 54.**

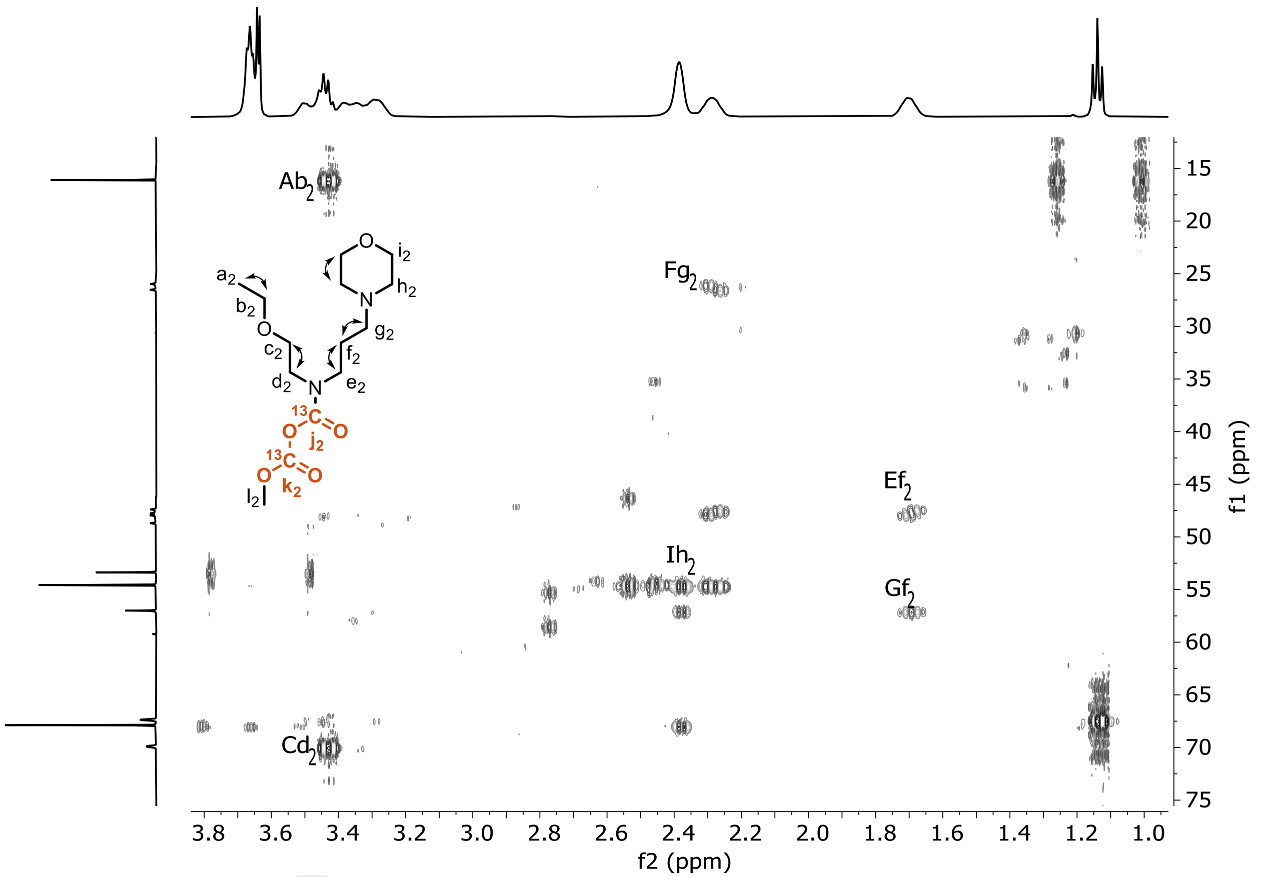

Supplementary Fig. 57. Extension of relevant region of HMBC NMR of purified **E(2)Me** obtained from $^{13}CO_2$ and $TMSCHN_2$ (1 eq) in the presence of 1 eq. of MeOH. Capital letters refer to carbon (C_x) nuclei, while lower case letters relate to protons (H_x). HMBC was recorded to establish long-range proton-carbon coupling (^nJCH) within ethylenic moieties (see double arrows). No conclusive correlation involving carbon j_2 and k_2 could be obtained.

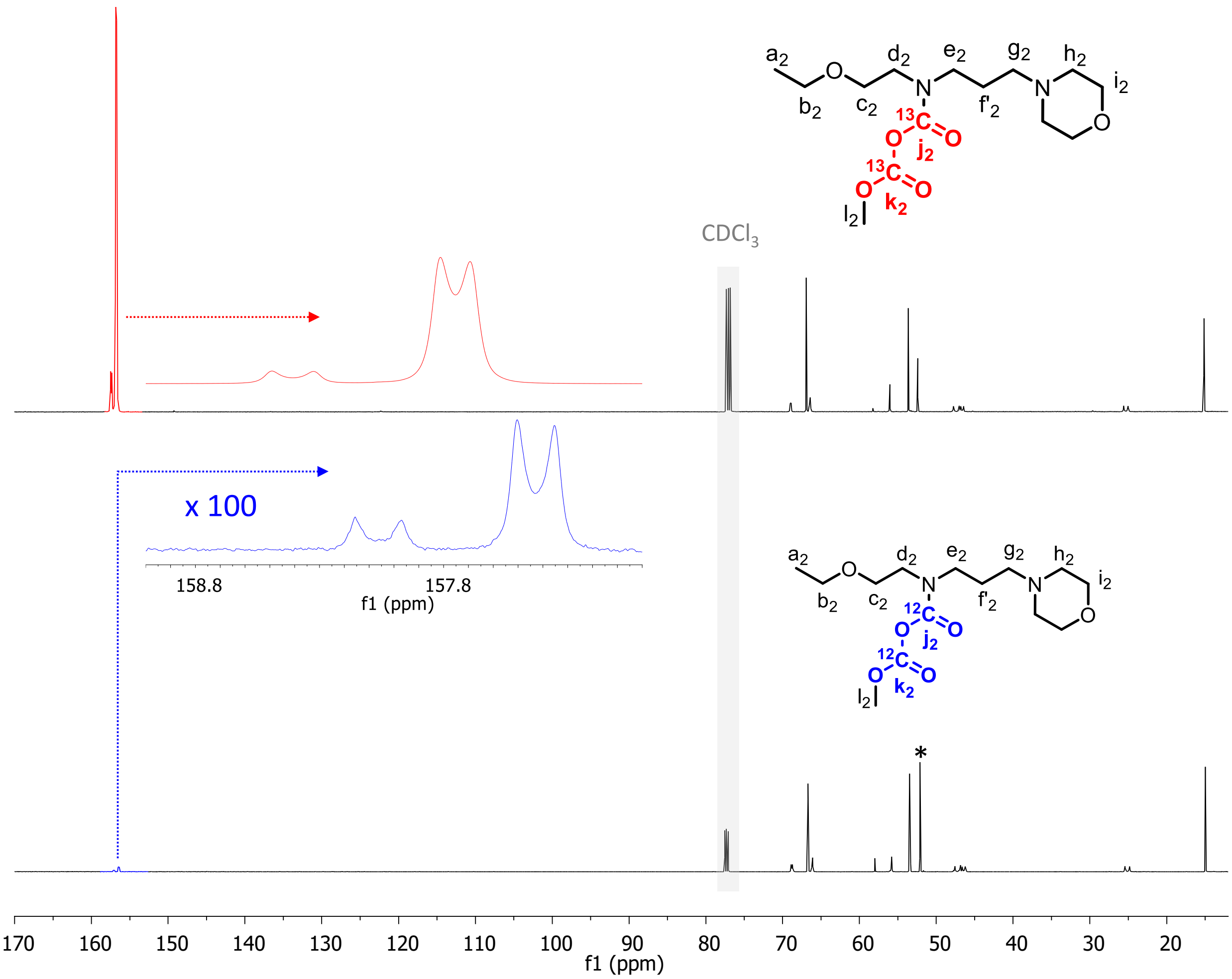

Supplementary Fig. 58. Comparison of the ^{13}C NMR spectra of purified **E(2)Me** obtained from $^{13}CO_2$ (top) and from natural abundance CO_2 (bottom), both using $TMSCHN_2$ (1 eq) with a zoom on the carbonyl region (intensity is magnified by a factor 100 in the case of natural abundance sample). The asterisk indicates a difference in intensity which is investigated in **Supplementary Fig. 60.**

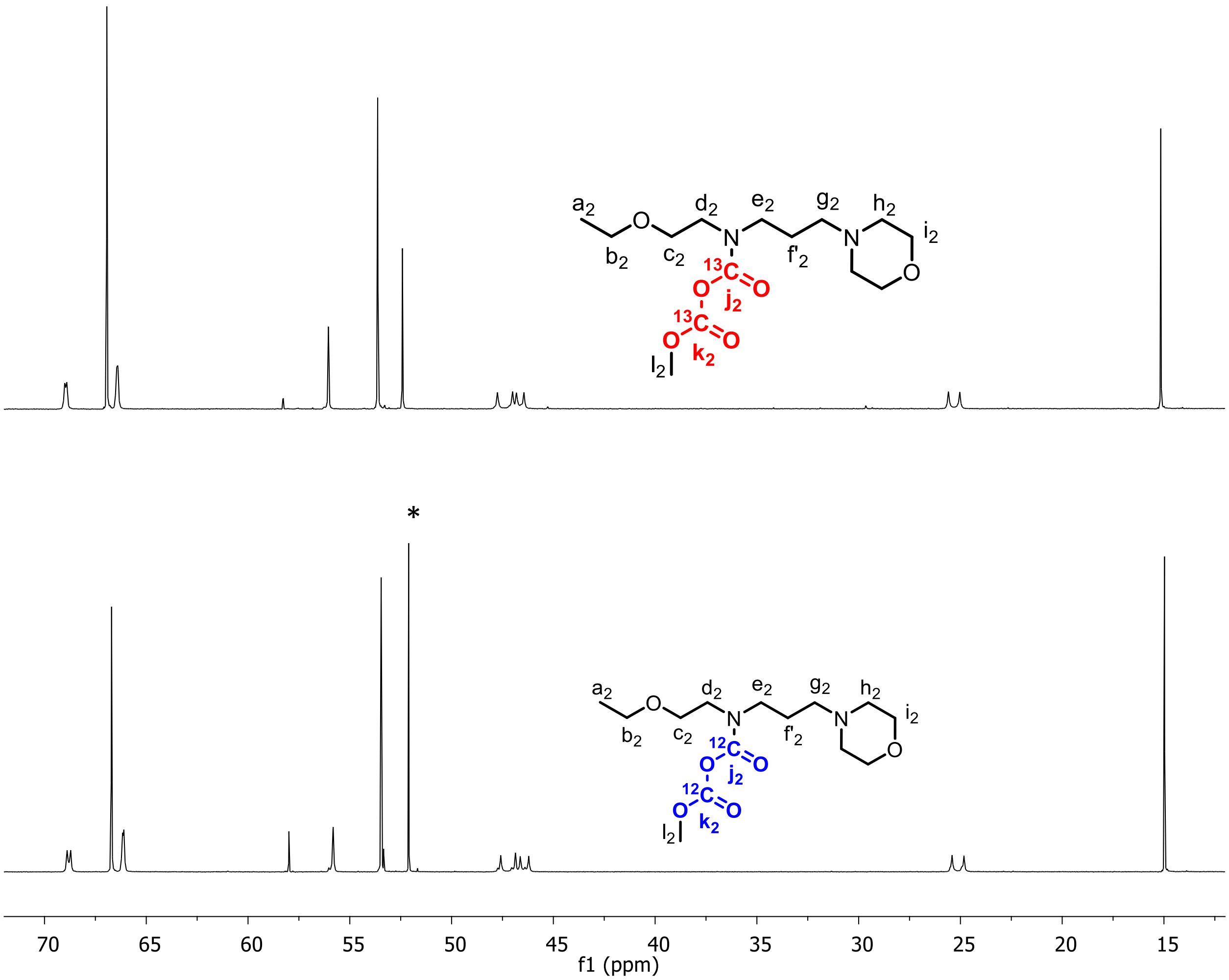

Supplementary Fig. 59. Comparison of the ^{13}C NMR spectra of purified **E(2)Me** obtained from ^{13}C-labelled (top) and natural abundance CO_2 (bottom) in the 12-72 ppm area (1 eq. $TMSCHN_2$). The asterisk indicates a difference in intensity which is investigated in **Supplementary Fig. 60.**

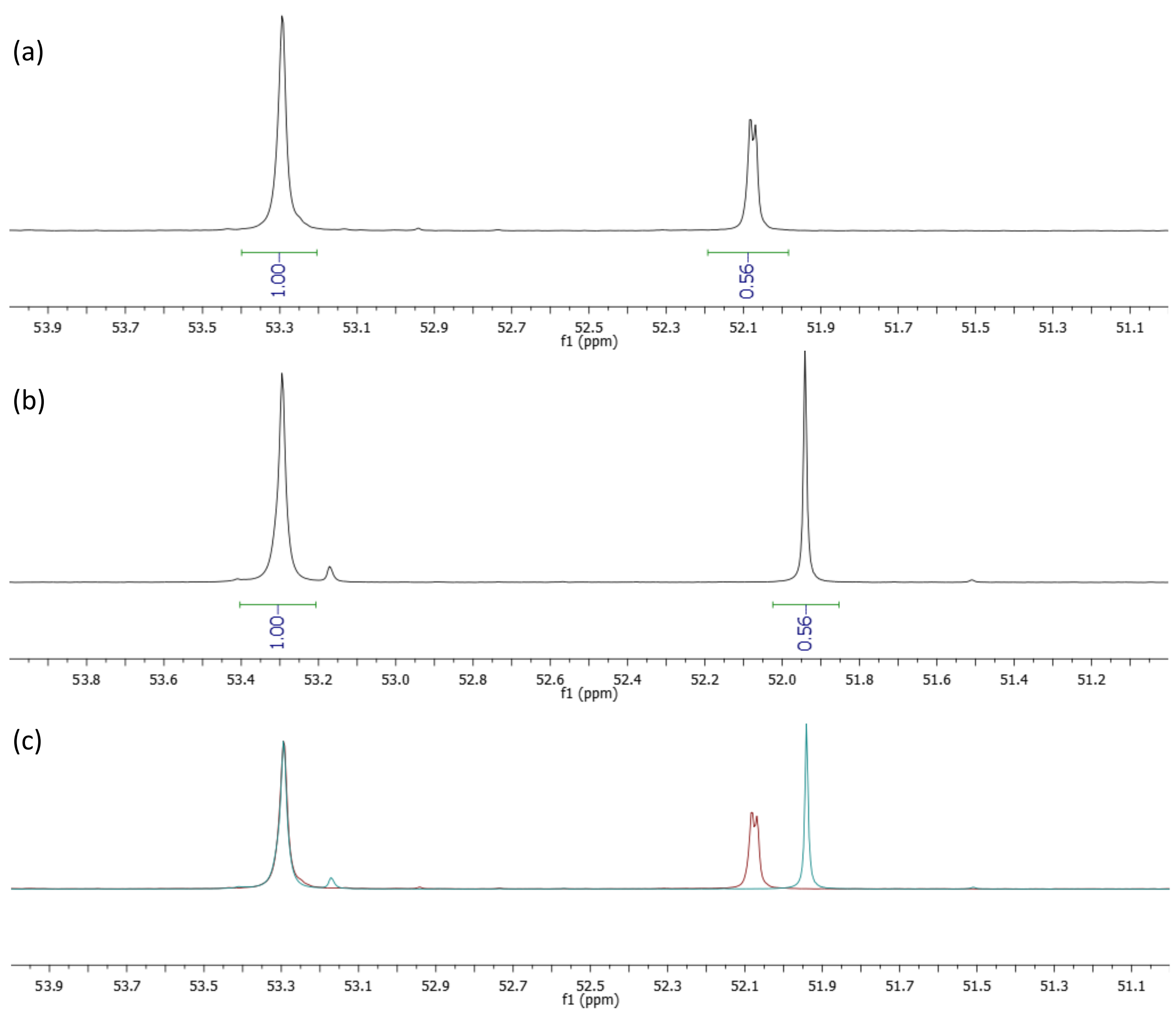

Supplementary Fig. 60. Comparison of the ^{13}C NMR spectra of purified **E(2)Me** obtained from ^{13}C-labelled (a) and natural abundance CO_2 (b) and superimposition of both (c) in the 51-54 ppm area (1 eq. $TMSCHN_2$). The peak marked with an asterisk at **Supplementary Figs. 58** and **59** varies in intensity and chemical shift as a consequence of the isotopic enrichment but its intensity remains constant.

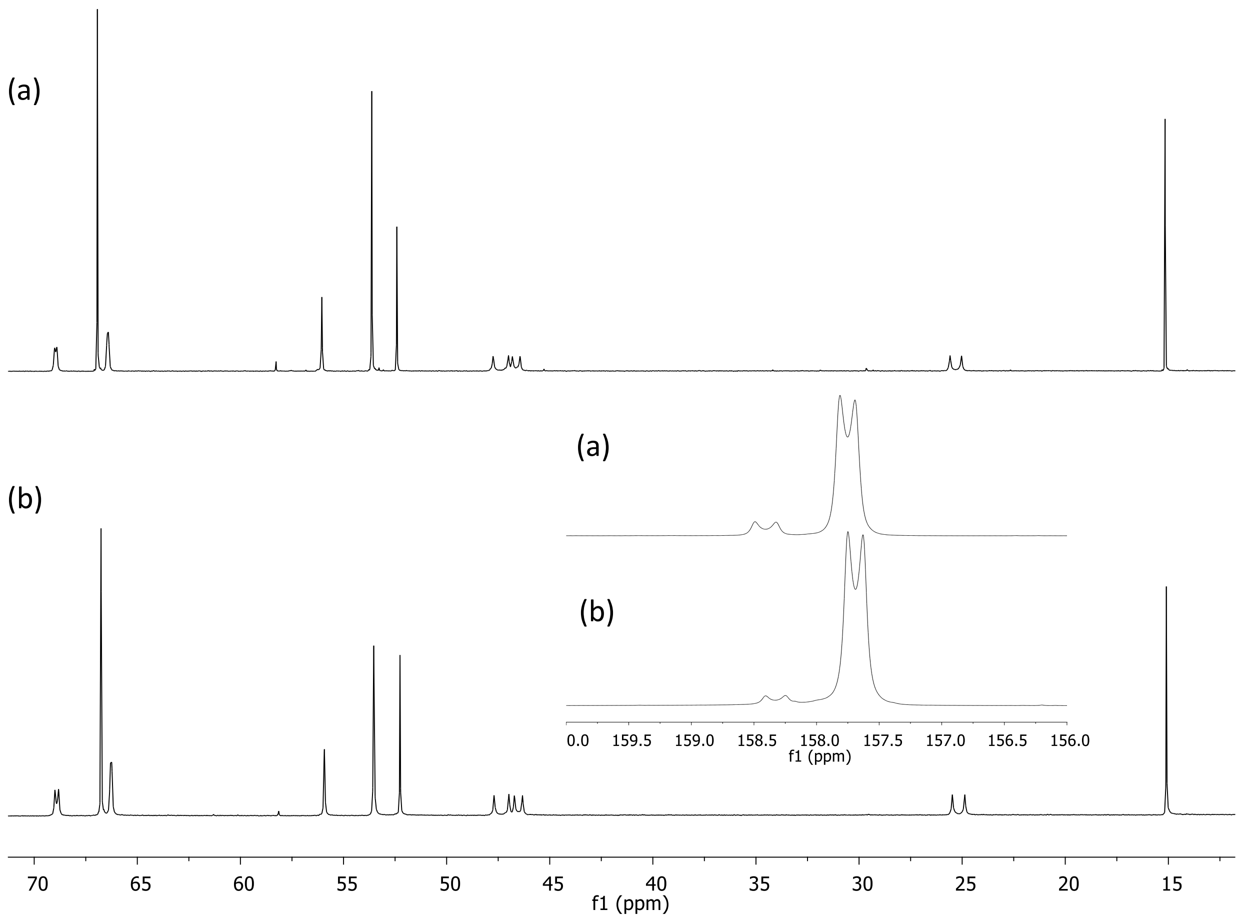

Supplementary Fig. 61. Comparison of ^{13}C NMR spectra of (a) purified **E(2)Me** obtained in the absence and (b) crude **E(2)Me** obtained in the presence of 1 eq. of MeOH.

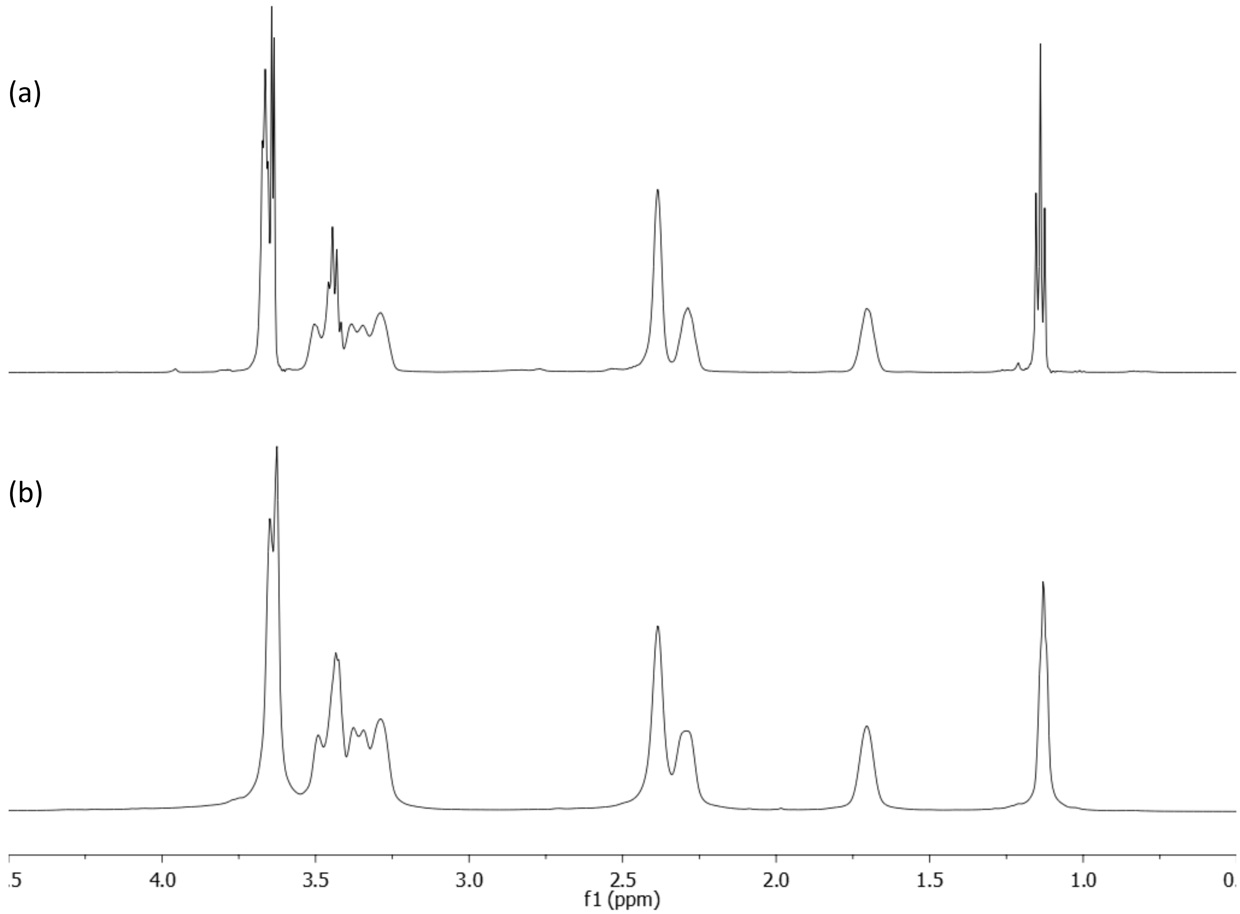

Supplementary Fig. 62. Comparison of ^{1}H NMR spectra of (a) purified **E(2)Me** obtained in the absence and (b) crude **E(2)Me** obtained in the presence of 1 eq. of MeOH. See **Supplementary Fig. 44** for assignment.

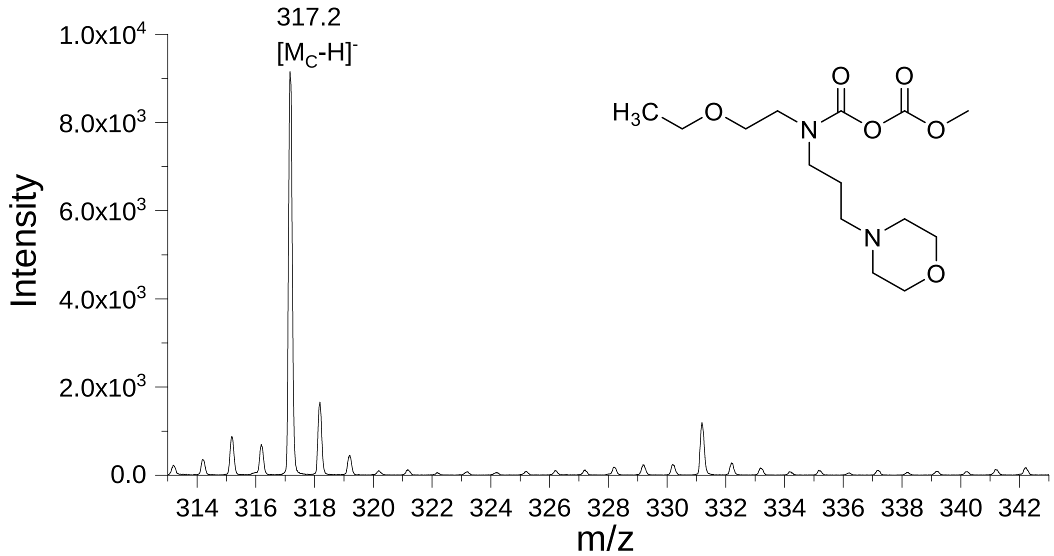

Supplementary Fig. 63. (-)-LR-TOF SIMS spectrum of purified **E(2)Me** obtained from natural abundance **CO_2**. Found for [**E(2)Me-H**] : 317.2 (Calcd 317.2).

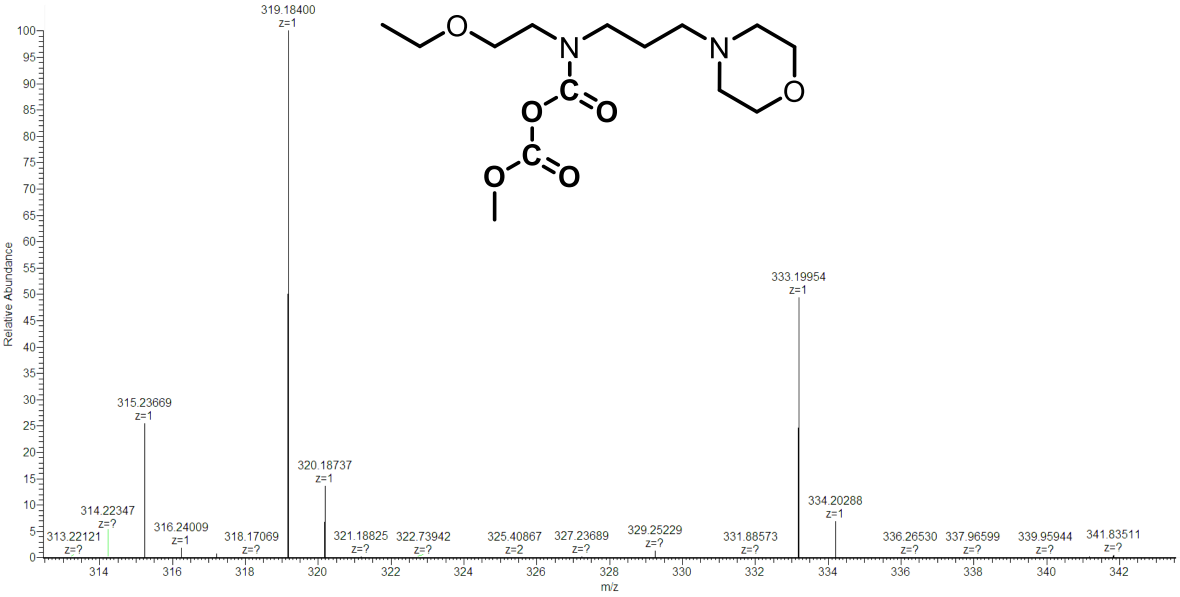

Supplementary Fig. 64. (+)-HR-ESI/MS spectrum of purified **E(2)Me** obtained from natural abundance **CO_2**. Found for **$E(2)MeH^+$** : 319.1840 (Calcd 319.1869) and **$E(2)Me_2^+$** : 333.1996 (calcd : 333.2026).

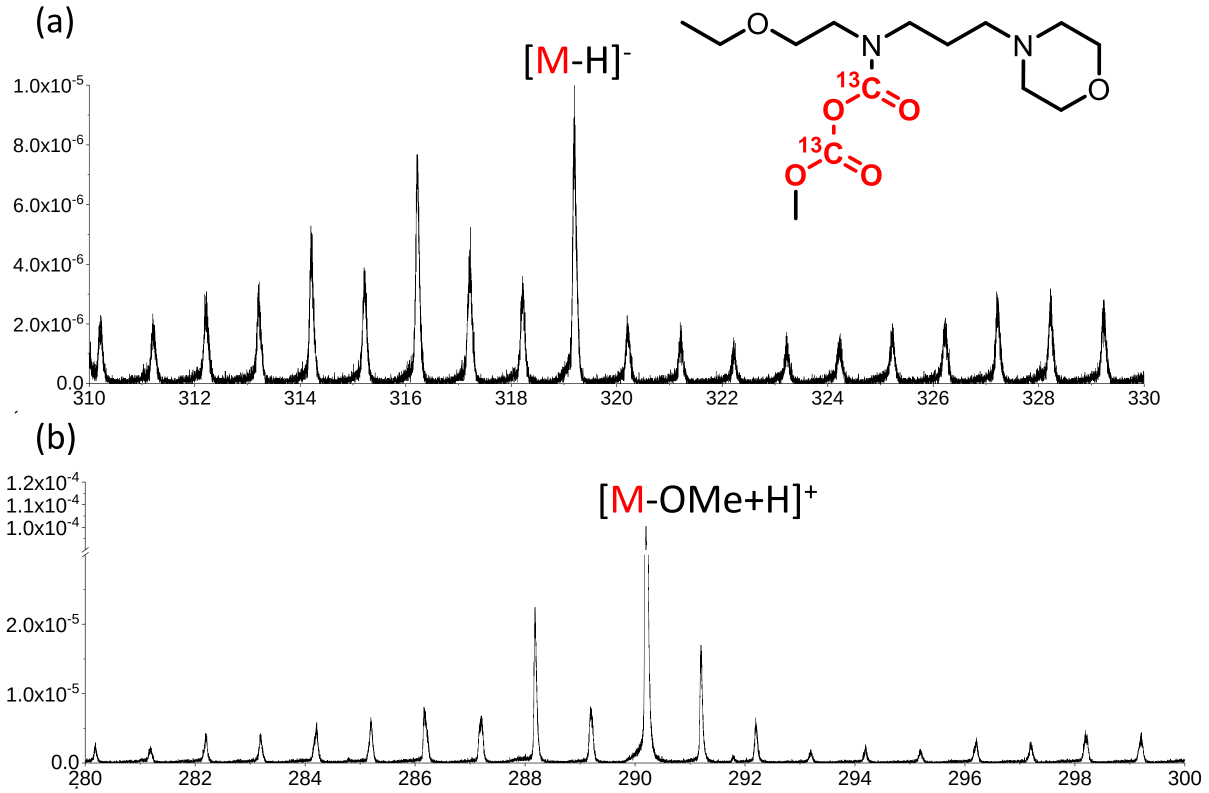

Supplementary Fig. 65. LR-TOF SIMS spectrum of crude **E(2)Me** obtained from ^{13}**CO_2** in (a) negative (calcd for $^{13}C_2C_{12}H_{24}N_2O_6$ [M-H]- 319.18, found 319.18) and (b) positive mode (Calcd for $^{13}C_2C_{11}H_{24}N_2O_5$ [M-OCH_3+H]$^+$ 290.18, found 290.18

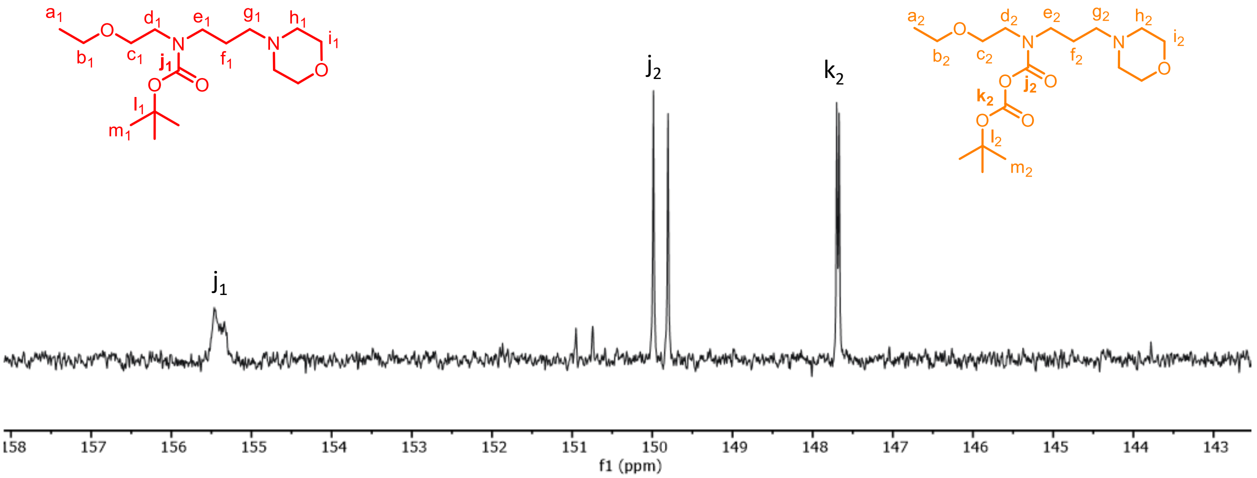

Supplementary Fig. 66. 140-160 ppm region of the ^{13}C NMR of **E(2)^{t}Bu** (orange) containing traces of **E(1)^{t}Bu** (red).

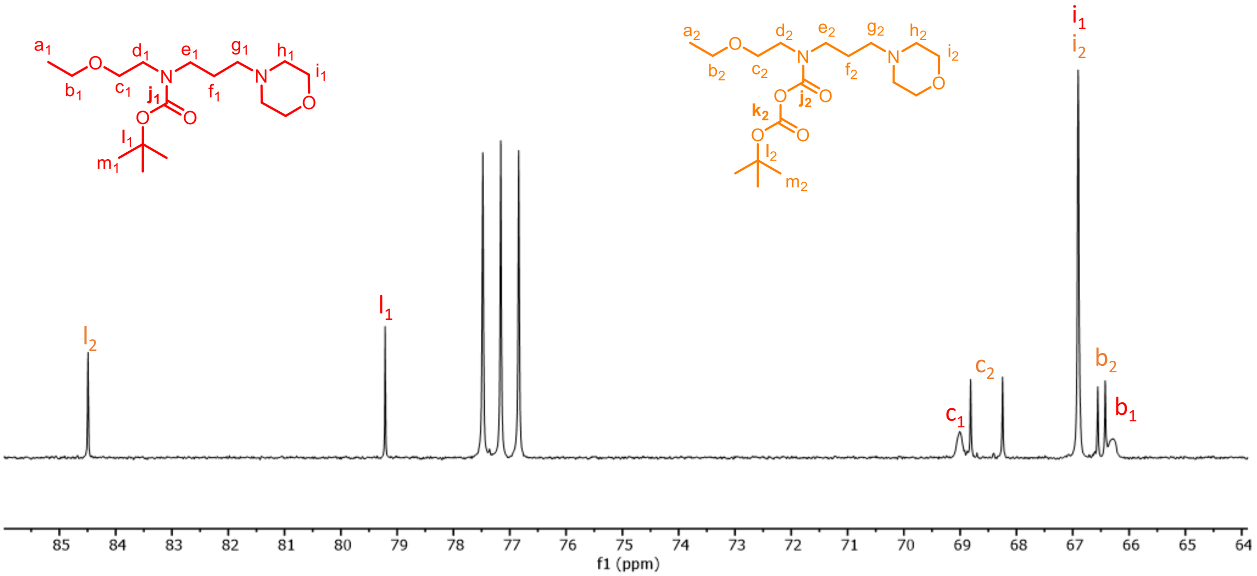

Supplementary Fig. 67. 60-90 ppm region of the ^{13}C NMR of **E(2)^{t}Bu** (orange) containing traces of **E(1)^{t}Bu** (red).

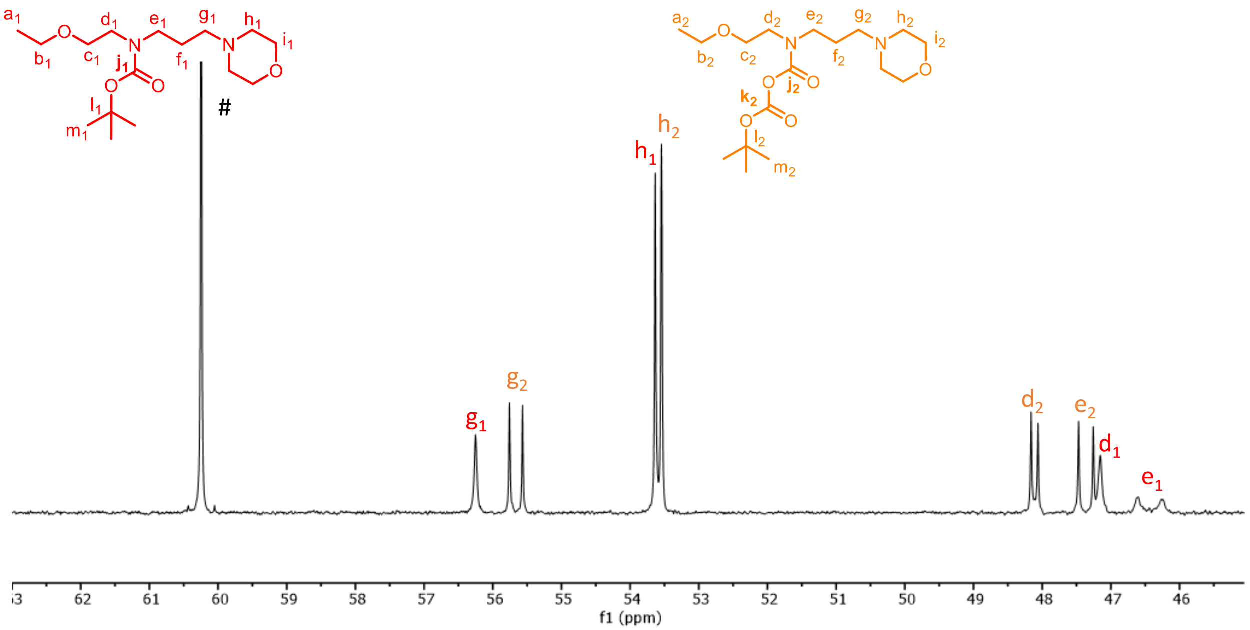

Supplementary Fig. 68. 40-70 ppm region of the ^{13}C NMR of **E(2)^{t}Bu** (orange) containing traces of **E(1)^{t}Bu** (red).

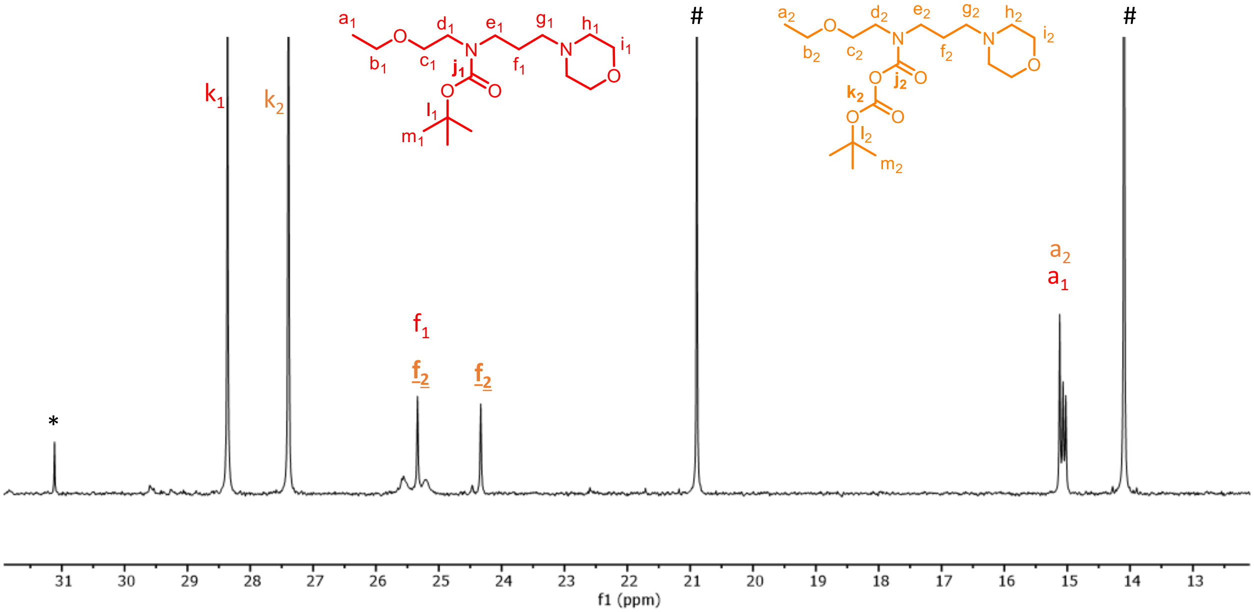

Supplementary Fig. 69. 10-40 ppm region of the ^{13}C NMR of **E(2)^{t}Bu** (orange) containing traces of **E(1)^{t}Bu** (red). * and # respectively mark traces of AcOEt and tBuOH.

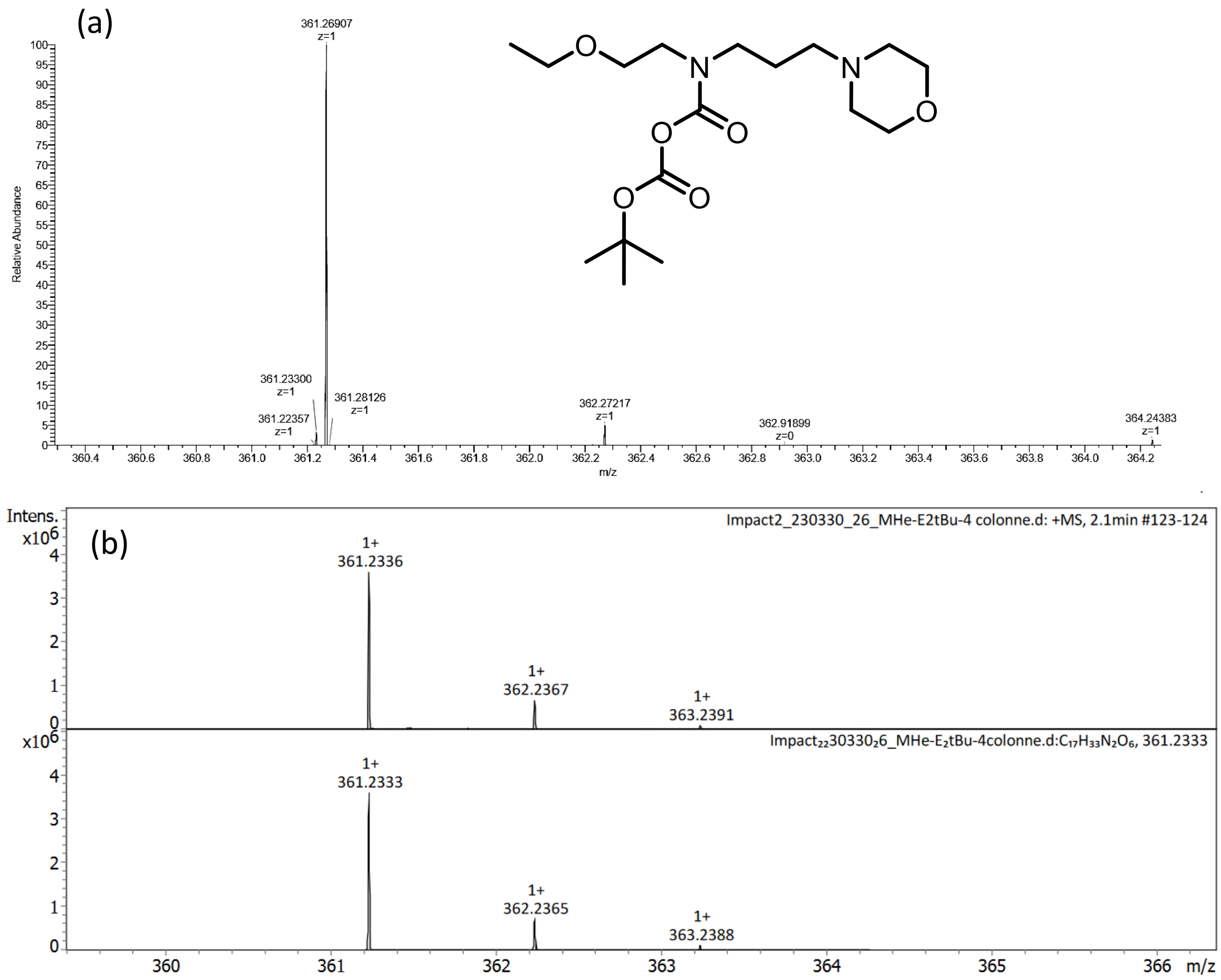

Meas. m/z	Ion Formula	Sum Formula	err [ppm]	mSigma	Adduct	z
361.2336	C17H33N2O6	C17H32N2O6	-0.7	6.1	M+H	1+

Supplementary Fig. 70. (+) HR-ESI MS spectrum of purified **E(2)^{t}Bu** (a) and comparison between experimental and theoretical pattern (b)**.**

DFT-computed conformational energies on **E(2)R** derivatives

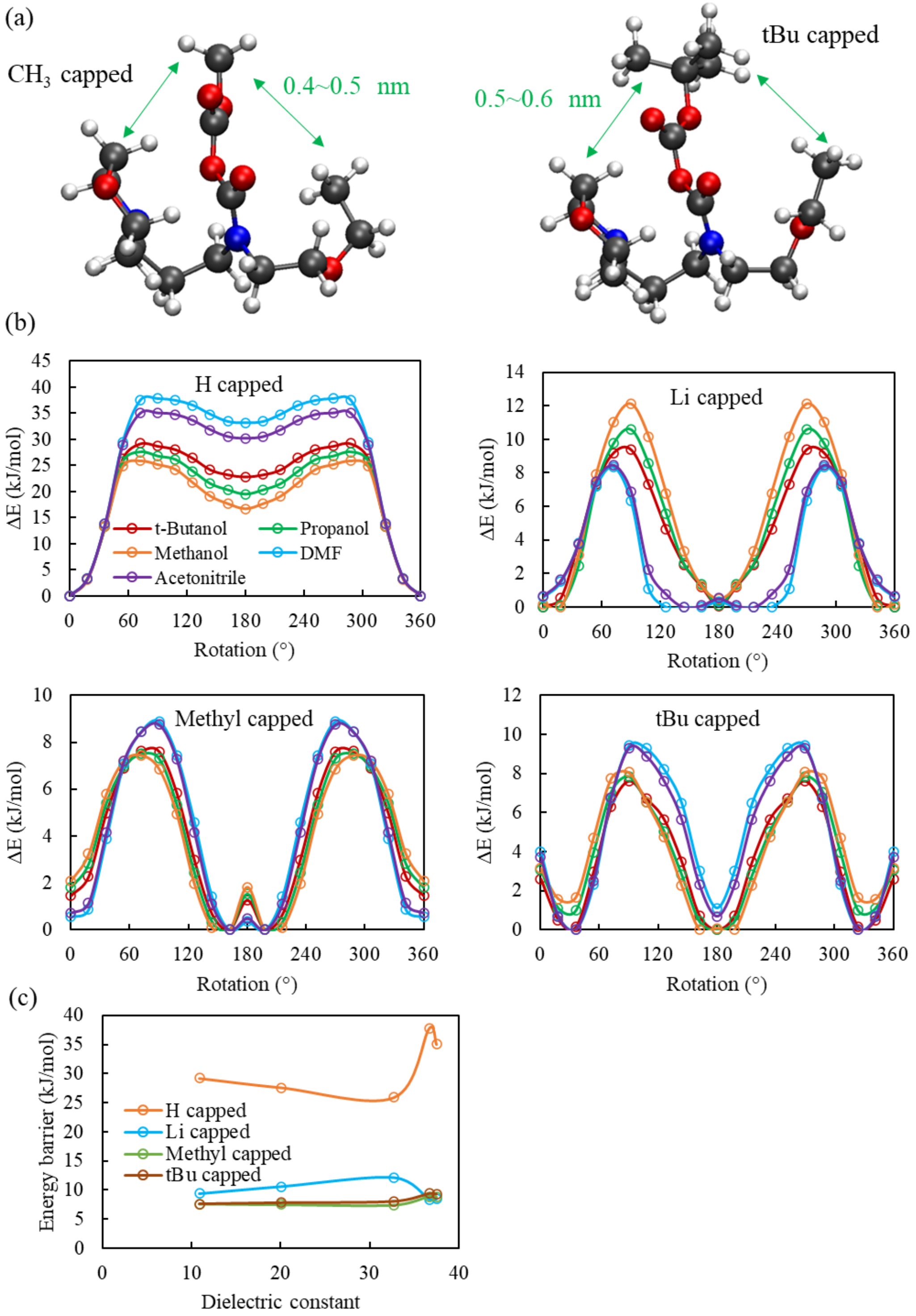

Supplementary Fig. 71. (a) Snapshot of the minimized structure of **E(2)Me** and **E(2)^{t}Bu** obtained by DFT calculations. (b) Rotamer landscape for EEMPA anhydrides **E(2)R** with different capping

agents (R = Li, H, Me, tBu) in different solvents. Capping agent effectively changes the rotational landscape. (c) The energy barriers between rotamers is relatively insensitive to the dielectrics of the medium and is more affected by the nature of the end group

Our calculations above (**Supplementary Fig. 71**) have shown that the dielectrics of the NMR solvent has a limited impact on the conformational landscape of **E(2)R** derivatives. Furthermore, even though the tetra-butyl cap has a significantly larger size than the other groups, the rotational barrier is not affected much by the steric hindrance of this moiety. This is due to the large conformational space available for the rotation of the capping group. On the other hand, the intra-molecular hydrogen bonding in the H capped anhydride results in higher rotational barrier. This is in line with the experimental data displayed at **Supplementary Figs. 55, 58, 60** and **65**.

Supplementary Tables

<u>Abbreviations and schematic representations</u>

Supplementary Table 1.
Building blocks involved in CO_2 capture / Constituents of dynamic covalent system.

**Constituent/building block**	Formula	Symbol
**E**	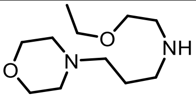	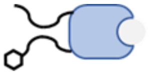
**CO_2**	CO_2	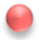

Supplementary Table 2.
Adducts from capture / resulting from reversible covalent linkage between constituents.
Components for non-covalent clustering into dimers/tetramers.

**Component/adduct**		Formula	Symbol
**$E(0)^{(+)}$**	**E(0)**	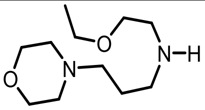	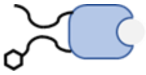
	**$E(0)^+$**	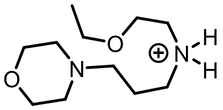	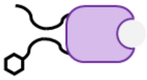
**$E(1)^{(-)}$**	**$E(1)^-$**	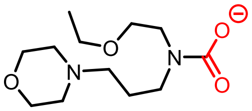	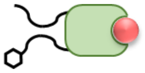
	**E(1)**	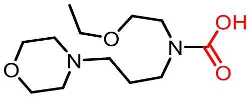	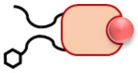

Supplementary Table 3.
Adducts from capture / resulting from reversible covalent linkage between constituents. Components for non-covalent clustering into dimers/tetramers.

**(trapped) Intermediate**	Formula	Symbol
**$E(2)^{-}$**	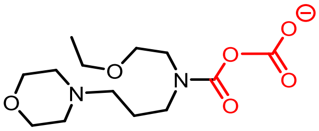	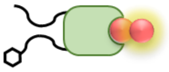
**$E(1)^{Z}$**	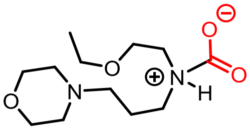	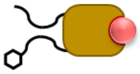
**E(2)Me**	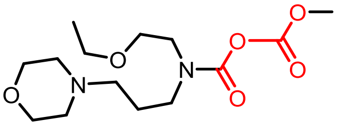	N/A
**$E(2)^{t}Bu$**	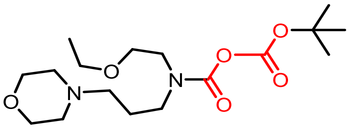	N/A

Supplementary Table 4.
Dimeric clusters resulting from non-covalent assembling of components/capture adducts

**Dimer/cluster**		Formula	Symbol
**E^2**	**$E^2(0)$**	**E(0)E(0)**	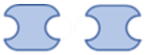
	**$E^2(0.5)$**	**$E(1)^{-}E(0)^{+}$**	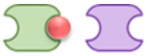
	**$E^2(1)$***	**$E(2)^{-}E(0)^{+}$**	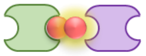
	**$E^2(1)$**	**E(1)E(1)**	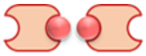

Supplementary Table 5.
Tetrameric clusters resulting from non-covalent assembling of components/capture adducts

Tetramer/cluster		Formula	Symbol
	**$E^4(0)$**	**E(0) E(0) E(0) E(0)**	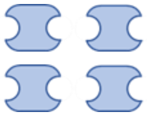
	**$E^4(0.25)$**	**$E(1)^-E(0)^+E(0)$ E(0)**	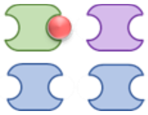
	**$E^4(0.5)$**	**$E(1)^-E(0)^+E(1)^-E(0)^+$**	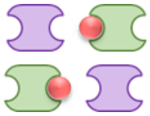
	**$E^4(0.5)^N$**	**$E(1)^-E(0)^+$ E(1)E(0)**	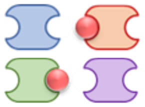
**E^4**	**$E^4(0.75)^N$**	**E(1) E(0) E(1) E(1)**	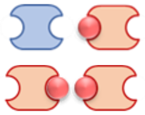
	**$E^4(0.75)^Z$**	**$E(1)^-E(0)^+$ $E(1)E(1)^z$**	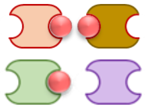
	**$E^4(0.75)$***	**$E(2)^-E(0)^+$ $E(1)^-E(0)^+$**	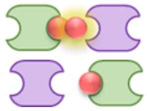
	**$E^4(0.75)$**	**$E(1)^-E(0)^+E(1)E(1)$**	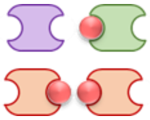
	**$E^4(1)^Z$**	**$E(1)E(1)^ZE(1)E(1)$**	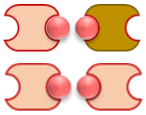
	**$E^4(1)$**	**E(1)E(1)E(1)E(1)**	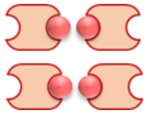

Binding isotherms and speciation data

Supplementary Table 6.
EEMPA/CO_2 samples analyzed by q^{13}C NMR (**Supplementary Figs. 12-15**). Correspondence between initial stoichiometry x_0 and chemical loading α.

x_0	α
0.000	0.000
0.077	0.077
0.143	0.143
0.176	0.176
0.274	0.272
0.359	0.356
0.374	0.371
0.472	0.467
0.534	0.520
0.646	0.597
0.993	0.746
1.616	0.809
2.352	0.842
5.054	0.950

Supplementary Table 7. RMSD values of the fit of experimental ^{13}C chemical shifts and loading values vs x_0 with the dimeric (left) and tetrameric model. (Right) (Averaged RMSD : bold font, RMSD values below an arbitrary 0.95 cutoff : red).

	$R^2(\mathbf{E}^2)$	$R^2(\mathbf{E}^4)$
alpha	0.9931	0.9996
CO	**0.6181**	0.997
c'	0.9745	0.9914
c	0.9809	0.9991
i,i’	0.9626	0.9986
b,b’	0.9118	0.9618
g	0.9618	0.9959
g'	0.9829	0.9895
h	0.9869	0.9895
h'	0.951	0.9825
d	0.9783	0.9979
e	0.9808	0.9979
d'	0.8848	0.9652
e'	0.9727	0.9825
f	0.969	0.9963
f'	0.935	0.9982
a'	0.9368	0.9759
a	0.9427	0.9883
	**0.9370**	**0.993**

WAXS parameters

Supplementary Table 8. WAXS TS+3G model, Teubner Strey Parameters.

α	A_scale (cm^{-1})	A_scale (Err)	A_d (Å)	A_d (Err)	A_xi (Å)	A_xi (Err)
0.03	0.0035	0.0045	21.96	1.16	7.96	4.82
0.08	0.0036	0.0097	20.34	2.24	6.10	6.76
0.20	0.0047	0.0071	20.58	1.31	6.71	4.46
0.36	0.0086	0.0220	20.82	2.72	4.53	3.69
0.58	0.1932	0.0161	11.93	0.08	4.20	0.16
0.72	0.3782	0.0083	11.90	0.02	5.92	0.08
0.79	0.4228	0.0097	11.85	0.02	5.65	0.08
0.84	0.4245	0.0091	11.85	0.02	5.71	0.07
0.86	0.4244	0.0088	11.81	0.02	5.81	0.07

Supplementary Table 9. WAXS TS+3G model, Gauss Peak 1 parameters.

α	B_scale (cm^{-1})	B_scale (Err)	B_peak_pos (Å^{-1})	B_peak_pos (Err)	B_sigma (Å^{-1})	B_sigma (Err)
0.03	0.1319	0.0024	1.326	0.0008	0.216	0.0026
0.08	0.1309	0.0028	1.326	0.0009	0.213	0.0029
0.20	0.1315	0.0024	1.328	0.0008	0.211	0.0026
0.36	0.1354	0.0035	1.328	0.0010	0.212	0.0035
0.58	0.1274	0.0018	1.325	0.0009	0.207	0.0028
0.72	0.1277	0.0021	1.322	0.0006	0.174	0.0019
0.79	0.1239	0.0031	1.321	0.0007	0.177	0.0026
0.84	0.1277	0.0024	1.322	0.0006	0.175	0.0022
0.86	0.1307	0.0020	1.322	0.0006	0.173	0.0018

Supplementary Table 10. WAXS TS+3G model, Gauss Peak 2 parameters.

α	C_scale (cm^{-1})	C_scale (Err)	C_peak_pos ($Å^{-1}$)	C_peak_pos (Err)	C_sigma ($Å^{-1}$)	C_sigma (Err)
0.03	0.0341	0.0020	1.378	0.0018	0.115	0.0026
0.08	0.0332	0.0022	1.377	0.0021	0.116	0.0029
0.20	0.0304	0.0021	1.377	0.0020	0.112	0.0030
0.36	0.0302	0.0023	1.374	0.0020	0.110	0.0031
0.58	0.0342	0.0024	1.356	0.0013	0.109	0.0026
0.72	0.0208	0.0025	1.344	0.0021	0.090	0.0044
0.79	0.0283	0.0037	1.337	0.0018	0.098	0.0044
0.84	0.0242	0.0030	1.336	0.0018	0.092	0.0043
0.86	0.0234	0.0025	1.340	0.0018	0.088	0.0039

Supplementary Table 11. WAXS TS+3G model, Gauss Peak 3 parameters.

α	D_scale (cm^{-1})	D_scale (Err)	D_peak_pos ($Å^{-1}$)	D_peak_pos (Err)	D_sigma ($Å^{-1}$)	D_sigma (Err)
0.03	0.0613	0.0021	1.339	0.0018	0.526	0.0330
0.08	0.0631	0.0029	1.338	0.0021	0.503	0.0373
0.20	0.0656	0.0024	1.333	0.0016	0.501	0.0302
0.36	0.0609	0.0050	1.334	0.0040	0.518	0.0721
0.58	0.0669	0.0019	1.374	0.0042	0.484	0.0107
0.72	0.1032	0.0011	1.394	0.0016	0.497	0.0046
0.79	0.1065	0.0012	1.402	0.0020	0.517	0.0054
0.84	0.1079	0.0011	1.401	0.0018	0.512	0.0050
0.86	0.1087	0.0010	1.402	0.0017	0.512	0.0046

References

1. Stoll, S. & Schweiger, A. EasySpin, a comprehensive software package for spectral simulation and analysis in EPR. *Journal of Magnetic Resonance* **178**, 42–55 (2006).

2. Cantu, D. C. *et al.* Molecular-Level Overhaul of γ-Aminopropyl Aminosilicone/Triethylene Glycol Post-Combustion CO2-Capture Solvents. *ChemSusChem* **13**, 3429–3438 (2020).

3. Subirats, X., Fuguet, E., Rosés, M., Bosch, E. & Ràfols, C. Methods for pKa Determination (I): Potentiometry, Spectrophotometry, and Capillary Electrophoresis. in *Reference Module in Chemistry, Molecular Sciences and Chemical Engineering* 1–10 (Elsevier, 2015). doi:10.1016/B978-0-12-409547-2.11559-8.

4. Basel, Y. & Hassner, A. Di- *tert* -butyl Dicarbonate and 4-(Dimethylamino)pyridine Revisited. Their Reactions with Amines and Alcohols. *J Org Chem* **65**, 6368–6380 (2000).

5. Jiang, Y. *et al.* Techno-economic comparison of various process configurations for post-combustion carbon capture using a single-component water-lean solvent. *International Journal of Greenhouse Gas Control* **106**, 103279 (2021).

6. Zheng, R. F. *et al.* A single-component water-lean post-combustion CO2capture solvent with exceptionally low operational heat and total costs of capture-comprehensive experimental and theoretical evaluation. *Energy Environ Sci* **13**, 4106–4113 (2020).

7. Gibbs, R. E. & Van Ness, H. C. Vapor—Liquid Equilibria from Total-Pressure Measurements. A New Apparatus. *Industrial and Engineering Chemistry Fundamentals* **11**, 410–4130 (1972).

8. Kamlet, M. J., Abboud, J.-L. M., Abraham, M. H. & Taft, R. W. Solvent Effects in Organic Chemistry. *J. J. Am. Chem. Soc* **48**, 23 (1983).

9. Brady, J. E. & Carr, P. W. An Analysis of Dielectric Models of Solvatochromism. *J. Phys. Chem* **89**, 5759–5766 (1985).

10. Teo, W. *et al.* Nile Red fluorescence spectroscopy reports early physicochemical changes in myelin with high sensitivity. *Proc Natl Acad Sci U S A* **118**, e2016897118 (2021).

11. Septavaux, J. *et al.* Simultaneous CO2 capture and metal purification from waste streams using triple-level dynamic combinatorial chemistry. *Nat Chem* **12**, 202–212 (2020).

12. Pugh, S. M. & Forse, A. C. Nuclear magnetic resonance studies of carbon dioxide capture. *Journal of Magnetic Resonance* **346**, 107343 (2023).

13. Walter, E. D. *et al.* Operando MAS NMR Reaction Studies at High Temperatures and Pressures. *Journal of Physical Chemistry C* **122**, 8209–8215 (2018).

14. Mehta, H. S. *et al.* A novel high-temperature MAS probe with optimized temperature gradient across sample rotor for in-situ monitoring of high-temperature high-pressure chemical reactions. *Solid State Nucl Magn Reson* **102**, 31–35 (2019).

15. Neese, F. The ORCA program system. *Wiley Interdiscip Rev Comput Mol Sci* **2**, 73–78 (2012).

16. Neese, F., Wennmohs, F., Becker, U. & Riplinger, C. The ORCA quantum chemistry program package. *Journal of Chemical Physics* **152**, (2020).

17. Becke, A. D. Density-functional exchange-energy approximation with correct asymptotic behavior. *Phys Rev A (Coll Park)* **38**, 3098–3100 (1988).

18. Lee, C., Yang, eitao & Parr, R. G. Development of the Colic-Salvetti correlation-energy formula into a functional of the electron density. *Phys Rev B* **37**, 785–789 (1988).

19. Grimme, S. Semiempirical GGA-type density functional constructed with a long-range dispersion correction. *J Comput Chem* **27**, 1787–1799 (2006).

20. Marenich, A. V., Cramer, C. J. & Truhlar, D. G. Universal solvation model based on solute electron density and on a continuum model of the solvent defined by the bulk dielectric constant and atomic surface tensions. *Journal of Physical Chemistry B* **113**, 6378–6396 (2009).

21. Merrick, J. P., Moran, D. & Radom, L. An evaluation of harmonic vibrational frequency scale factors. *Journal of Physical Chemistry A* **111**, (2007).

22. Scott, A. P. & Radom, L. Harmonic vibrational frequencies: An evaluation of Hartree-Fock, Møller-Plesset, quadratic configuration interaction, density functional theory, and semiempirical scale factors. *Journal of Physical Chemistry* **100**, (1996).

23. Tarrago, M., Ye, S. & Neese, F. Electronic structure analysis of electrochemical CO2 reduction by iron-porphyrins reveals basic requirements for design of catalysts bearing non-innocent ligands. *Chem Sci* **13**, (2022).

24. Laury, M. L., Carlson, M. J. & Wilson, A. K. Vibrational frequency scale factors for density functional theory and the polarization consistent basis sets. *J Comput Chem* **33**, (2012).

25. Decherchi, S., Masetti, M., Vyalov, I. & Rocchia, W. Implicit solvent methods for free energy estimation. *European Journal of Medicinal Chemistry* vol. 91 Preprint at https://doi.org/10.1016/j.ejmech.2014.08.064 (2015).

26. Riquelme, M. *et al.* Hydration Free Energies in the FreeSolv Database Calculated with Polarized Iterative Hirshfeld Charges. *J Chem Inf Model* **58**, (2018).

27. Zhang, J., Zhang, H., Wu, T., Wang, Q. & Van Der Spoel, D. Comparison of Implicit and Explicit Solvent Models for the Calculation of Solvation Free Energy in Organic Solvents. *J Chem Theory Comput* **13**, (2017).

28. Fogolari, F., Corazza, A. & Esposito, G. Free energy, enthalpy and entropy from implicit solvent end-point simulations. *Front Mol Biosci* **5**, (2018).

29. Zhou, R. Free energy landscape of protein folding in water: Explicit vs. implicit solvent. *Proteins: Structure, Function and Genetics* **53**, (2003).

30. Mobley, D. L., Dill, K. A. & Chodera, J. D. Treating entropy and conformational changes in implicit solvent simulations of small molecules. *Journal of Physical Chemistry B* **112**, (2008).

31. Draper, E. R. *et al.* Insight into the self-assembly of water-soluble perylene bisimide derivatives through a combined computational and experimental approach. *Nanoscale* **11**, (2019).

32. Redivo, L. *et al.* Prediction of self-assembly of adenosine analogues in solution: A computational approach validated by isothermal titration calorimetry. *Physical Chemistry Chemical Physics* **21**, (2019).

33. Antony, J., Sure, R. & Grimme, S. Using dispersion-corrected density functional theory to understand supramolecular binding thermodynamics. *Chemical Communications* vol. 51 Preprint at https://doi.org/10.1039/c4cc06722c (2015).

34. Miró, P. *et al.* Self-Assembly of Uranyl–Peroxide Nanocapsules in Basic Peroxidic Environments. *Chemistry - A European Journal* **22**, (2016).

35. Spaeth, J. R., Kevrekidis, I. G. & Panagiotopoulos, A. Z. A comparison of implicit- and explicit-solvent simulations of self-assembly in block copolymer and solute systems. *Journal of Chemical Physics* **134**, (2011).

36. Jorgensen, W. L.; Maxwell, D. S.; Tirado-Rives, J. Development and Testing of the OPLS All-Atom Force Field on Conformational Energetics and Properties of Organic Liquids. *Journal of the American Chemical Society*, **118**, 11225-11236, (1996). DOI: 10.1021/ja9621760

37. Hess, B.; Kutzner, C.; van der Spoel, D.; Lindahl, E. GROMACS 4: Algorithms for Highly Efficient, Load-Balanced, and Scalable Molecular Simulation. *Journal of Chemical Theory and Computation*, **4**, 435-447 (2008). DOI: 10.1021/ct700301q.

38. Dodda, L. S.; Cabeza de Vaca, I.; Tirado-Rives, J.; Jorgensen, W. L. LigParGen web server: an automatic OPLS-AA parameter generator for organic ligands. *Nucleic Acids Res.*, **45**, W331-W336, (2017). DOI: 10.1093/nar/gkx312.

39. Dodda, L. S.; Vilseck, J. Z.; Tirado-Rives, J.; Jorgensen, W. L. 1.14*CM1A-LBCC: Localized Bond-Charge Corrected CM1A Charges for Condensed-Phase Simulations. *J. Phys. Chem. B*, **121**, 3864-3870, (2017). DOI: 10.1021/acs.jpcb.7b00272.

40. Aprà, E.; Bylaska, E. J.; de Jong, W. A.; Govind, N.; Kowalski, K.; Straatsma, T. P.; Valiev, M.; van Dam, H. J. J.; Alexeev, Y.; Anchell, J.; et al. NWChem: Past, present, and future. *The Journal of Chemical Physics*, **152**, (2020). DOI: 10.1063/5.0004997.

41. Bernetti, M., Bussi, G., Pressure control using stochastic cell rescaling. *The Journal of Chemical Physics* **153**, 114107, (2020).

42. Evans, D. J., Holian, B. L., The Nose-Hoover thermostat. *J. Chem. Phys.*, **83**, 4069–4074, (1985).

43. Teubner, M. & Strey, R. Origin of the scattering peak in microemulsions. *J Chem Phys* **87**, 3195 (1987).

44. Schubert, K. V., Strey, R., Kline, S. R. & Kaler, E. W. Small angle neutron scattering near Lifshitz lines: Transition from weakly structured mixtures to microemulsions. *J Chem Phys* **101**, 5343–5355 (1994).

45. Morkved, T. L., Stepanek, P., Krishnan, K., Bates, F. S. & Lodge, T. P. Static and dynamic scattering from ternary polymer blends: Bicontinuous microemulsions, Lifshitz lines, and amphiphilicity. *Journal of Chemical Physics* **114**, 7247 (2001).

46. Bañuelos, J. L. *et al.* Subtle changes in hydrogen bond orientation result in glassification of carbon capture solvents. *Physical Chemistry Chemical Physics* **22**, 19009–19021 (2020).

47. Ottaviani, M. F., Martini, G. & Nuti, L. Nitrogen Hyperfine Splitting Constant of Nitroxide Solutions: Differently Structured and Charged Nitroxides as Probes of Environmental Properties. *Magnetic Resonance in Chemistry* **25**, 897–904 (1987).

48. Forse, A. C. *et al.* Elucidating CO 2 Chemisorption in Diamine-Appended Metal− Organic Frameworks. *J Am Chem Soc* **140**, 18016–18031 (2018).

49. Kortunov, P. V., Siskin, M., Baugh, L. S. & Calabro, D. C. In Situ Nuclear Magnetic Resonance Mechanistic Studies of Carbon Dioxide Reactions with Liquid Amines in Non-aqueous Systems: Evidence for the Formation of Carbamic Acids and Zwitterionic Species. *Energy and Fuels* **29**, 5940–5966 (2015).

50. Masuda, K., Ito, Y., Horiguchi, M. & Fujita, H. Studies on the solvent dependence of the carbamic acid formation from ω-(1-naphthyl)alkylamines and carbon dioxide. *Tetrahedron* **61**, 213–229 (2005).

51. Hunter, C. A. & Anderson, H. L. What is cooperativity? *Angewandte Chemie - International Edition* **48**, 7488–7499 (2009).

52. Masuda, K., Ito, Y., Horiguchi, M. & Fujita, H. Studies on the solvent dependence of the carbamic acid formation from u-(1-naphthyl)alkylamines and carbon dioxide. *Tetrahedron* **61**, 213–229 (2004).

53. Switzer, J. R. *et al.* Reversible Ionic Liquid Stabilized Carbamic Acids: A Pathway Toward Enhanced CO_2 Capture. *Ind Eng Chem Res* **52**, 13159–13163 (2013).

54. Didas, S. A., Sakwa-Novak, M. A., Foo, G. S., Sievers, C. & Jones, C. W. Effect of Amine Surface Coverage on the Co-Adsorption of CO_2 and Water: Spectral Deconvolution of Adsorbed Species. *J Phys Chem Lett* **5**, 4194–4200 (2014).

55. Nottingham, C. Trimethylsilyldiazo[13C]methane: A Versatile 13C-Labelling Reagent. *Organic Syntheses* **95**, (2018).
