# Supplementary material for: Tetrameric self-assembling of water-lean solvents enables carbamate anhydride-based CO2 capture chemistry
Source: Nat Chem. 2024 Apr 8;16(7):1160–8. doi: 10.1038/s41557-024-01495-z (PMC11230897; doi:10.1038/s41557-024-01495-z)
Supplement: Supplementary file 2 — MATLAB code for dimeric model. [file 41557_2024_1495_MOESM2_ESM.docx]

**%% General script for the concomitant fitting of association constants for three species, as**

**%%in "Nano-Clustering in Water-Lean Solvents Establishes Novel CO2 Chemistry"**

**%%from L. Leclaire et al.**

%% Let's get some data

EMPA0 = 1 ;% Molar fraction

Data = DATA ;%first column is CO2 charge, others are C13 chemical shifts

Data(:,1)=DATA(:,1)*10; % weighting of CO2 charge, arbitrary

%X0 is the total amount of CO2 in the system with respect to EEMPA

%% Parameter Fitting

fun = @(K,CO2i) Avancement(X0,K,EMPA0,0,Data);

Nb_Iter =30; % Number of fits with different starting points

LB = [100;0]; %Lower boundaries of parameters

UB = [100000;100]; %Upper boundaries of parameters

Nb_Par = size(LB,1); %Number of parameters

MC_Result = zeros(Nb_Iter,Nb_Par+1);

options = optimoptions('lsqcurvefit','Algorithm','levenberg-marquardt');

for i=1:Nb_Iter

Constants0 = LB + rand(Nb_Par,1).*(UB-LB);

%Constants0 = [1000;100;10;1]; %Initiation of parameters. Uncomment for fixed starting point

[Kres, resnorm] = lsqcurvefit(fun,Constants0,X0,Data, LB, UB); % Kres contains the fitting parameters after fitting

MC_Result(i,1:Nb_Par)=Kres.';

MC_Result(i,Nb_Par+1)=resnorm;

end

[resnorm, MC_Nb] = min(MC_Result(:,Nb_Par+1));

Kres = MC_Result(MC_Nb,1:Nb_Par).'%fitting result for the best fit

[Dataresult, Species, Shifts] = Avancement(X0,Kres,EMPA0,0,Data);

%%Constants

K1= Kres(1) %2 EMPA for 1 CO2.

K2= Kres(2) %2 EMPA for 2 CO2.

%% Coefficient of determination for each observable

Coeff_Deter = zeros(size(Data,2),1);

for j =1:size(Data,2)

Somme = 0;

e = 0;

Mean = mean(Data(:,j));

for i = 1:size(Data,1)

e = e + (Dataresult(i,j)-Data(i,j))^2;

Somme = Somme + (Data(i,j)-Mean)^2;

end

Coeff_Deter(j)=1-e/Somme;

end

Coeff_Deter

%% Global coefficient of determination

Moyenne = mean(mean(Data));

Somme = 0;

for i = 1:size(Data,2)

for j=1:size(Data,1)

Somme = Somme + (Data(j,i)-Moyenne)^2;

end

end

R2 = 1- resnorm/Somme

%% Plotting

%% Plotting

X0_Scale =power(10,[-1.3*20:0.8*20].'/20);

[Dataresult2, Species2] = Avancement_Graph(X0_Scale,Kres,EMPA0,Shifts);

figure

plot(X0_Scale,Dataresult2)

%set(gca,'ColorOrderIndex',1,'XScale','log')

figure

plot(X0,Data,'o')

hold on

set(gca,'ColorOrderIndex',1,'XScale','log')

%plot(Co2scale,TCresult,'-')

plot(X0,Dataresult,'-')

title('EMPA Predicted and observed')

%figure

%plot(Dataresult2, Species2)

%title('Speciation = f(TC)')

%figure

%plot(X0_Scale, Species2)

%title('Speciation = f(X0)')

%%set(gca,'ColorOrderIndex',1,'XScale','log')

**function [Result,Spe,Shifts] = Avancement(X0,K,EMPA0,Trig,DATA)**

%% For a set of parameters, calculate the expected 1H NMR signal displacement for each species.

Nb_Pts = size(X0,1);

Species = zeros(Nb_Pts,3); %Compounds

Result = zeros(Nb_Pts,1); %CO2 Loading signals

Constants = K; % Constants

Shifts = zeros(4, size(DATA,2)-1);

for i = 1:Nb_Pts

X = Function (EMPA0,Constants,X0(i));

Species(i,1:5)= X(1:5); % Concentration of species.

A(i)= Species(i,1); % EMPA

B(i)= Species(i,2); % 1 CO2

C(i)= Species(i,3); % 2 CO2

Result(i,1) = 0.5*B(i) + C(i) ;

end

LB = [-10;-10]; %Lower boundaries of parameters

UB = [10;10]; %Upper boundaries of parameters

Shift0=[0;0];

for i =2:size(DATA,2)

fun2 = @(K,X) Shift(X,K,Species);

[K, resnorm] = lsqcurvefit(fun2,Shift0,X0,DATA(:,i), LB, UB);

Shifts(:,i-1)=K;

Result(:,i)= Shift(X0,K,Species);

end

Spe = Species;

Result(:,1)=Result(:,1)*10;

end

**function [Result,Spe] = Avancement_Graph(X0,K,EMPA0,Shifts)**

%% For a set of parameters, calculate the expected 1H NMR signal displacement for each species.

Nb_Pts = size(X0,1);

Species = zeros(Nb_Pts,3); %Compounds

Result = zeros(Nb_Pts,1); %TC signals

Constants = K; % Constants

for i = 1:Nb_Pts

X = Function (EMPA0,Constants,X0(i));

Species(i,1:5)= X(1:5); % Concentration of species.

A(i)= Species(i,1); % EMPA

B(i)= Species(i,2); % 1 CO2

C(i)= Species(i,3); % 2 CO2

Result(i,1) = 0.5*B(i) + C(i) ;

end

for i =1:size(Shifts,2)

K=Shifts(:,i);

Result(:,i+1)= Shift(X0,K,Species);

end

Spe = Species;

Result(:,1)=Result(:,1)*10;

end

**function [Shift] = Shift(X0,K,Species)**

Shift = zeros(size(X0,1),1);

Shift(:,1) = K(1).*Species(:,2)+K(2).*Species(:,3);

end

**function [Species] = Function(EMPAi,Constants,CO2i)**

%% Calculate the concentration of unbound species as a function of association constant values.

options = optimoptions('fsolve','Display','off');

x0 = ones(1,4)/100;

x0(1)=log(EMPAi);

x = fsolve(@(Spec) Solver(Spec, Constants, EMPAi,CO2i),x0,options);

Species = real(x);

End

**function [X] = Solver(Species, Constants, EMPAi,CO2tot)**

%% Association constants

K1= Constants(1); %2 EMPA for 1 CO2.

K2= Constants(2); %2 EMPA for 2 CO2.

%% Species

A= Species(1); % EMPA2

B= Species(2); % EMPA2 CO2

C= Species(3); % EMPA2 2CO2

CO2 = Species(4);

%% Mass Balances on EMPA

X(1) = EMPAi - A - B – C;

%% Mass Balances on CO2

X(6) = CO2tot - CO2 - 0.5*B - C;

%% CO2 loading tiers

X(2) = B - K1*CO2*A;

X(3) = C - K2*CO2*B;

end
