# Supplementary material for: Tetrameric self-assembling of water-lean solvents enables carbamate anhydride-based CO2 capture chemistry
Source: Nat Chem. 2024 Apr 8;16(7):1160–8. doi: 10.1038/s41557-024-01495-z (PMC11230897; doi:10.1038/s41557-024-01495-z)
Supplement: Supplementary file 7 — Animation showing the 1 μs classical molecular dynamics trajectory of E4(0.25) and E4(0.5) clusters in a 25 mol% CO2 loading system. [file 41557_2024_1495_MOESM7_ESM.pptx]

## Slide 1
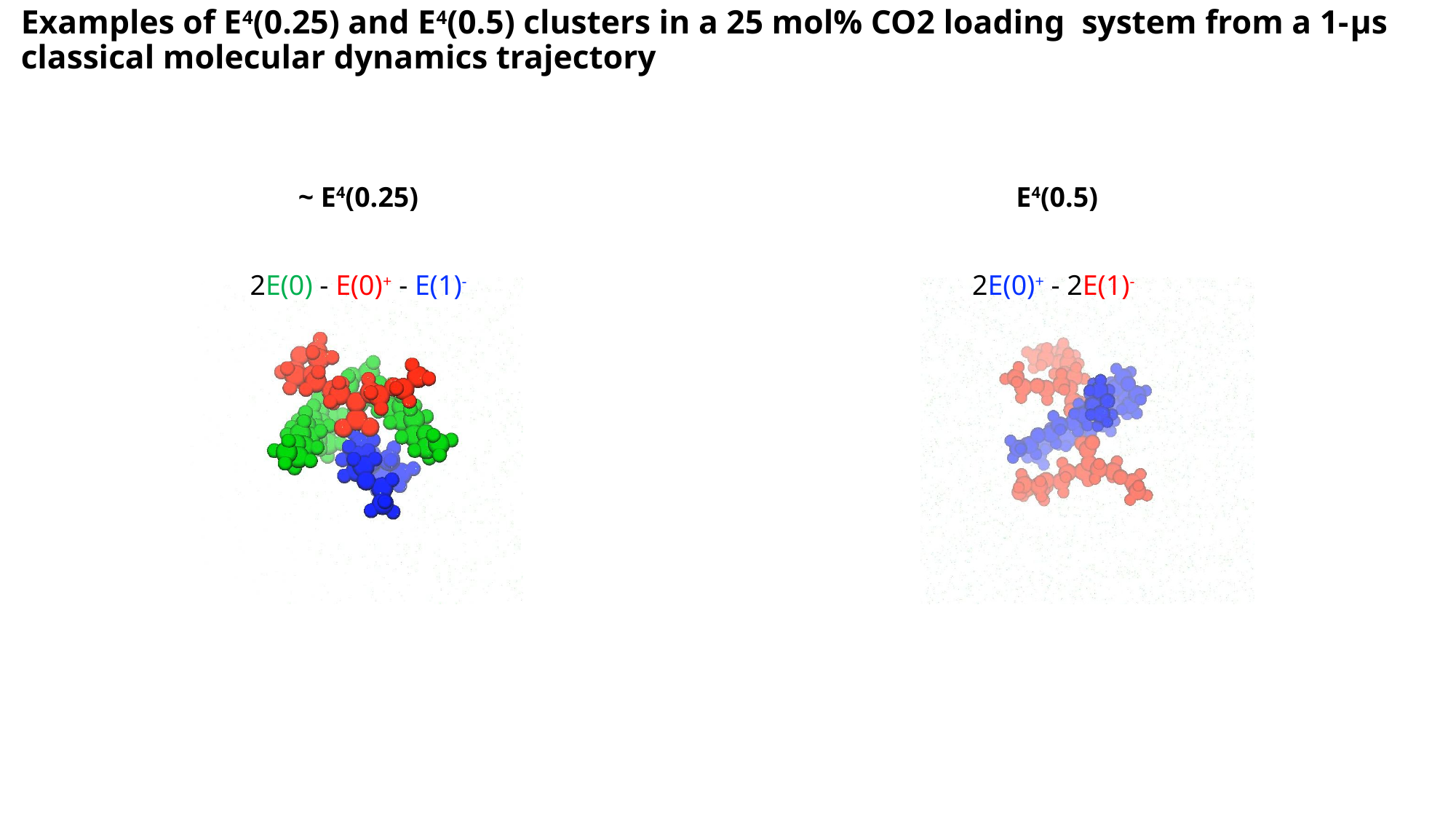

# Examples of E4(0.25) and E4(0.5) clusters in a 25 mol% CO2 loading system from a 1-μs classical molecular dynamics trajectory
~ E4(0.25)
 E4(0.5)
2E(0) - E(0)+ - E(1)-
2E(0)+ - 2E(1)-
